# Herbal medicine for asymptomatic hyperuricemia: a systematic review and network meta-analysis

**DOI:** 10.3389/fphar.2025.1627714

**Published:** 2025-09-29

**Authors:** Ye Min, Fengqin Xiao, Ling Zhou, Meng Fan, Jinli Luo, Linhua Zhao

**Affiliations:** ^1^ School of Basic Medicine, Gansu University of Chinese Medicine, Lanzhou, Gansu, China; ^2^ Guangdong Yifang Pharmaceutical Co., Ltd., Foshan, China; ^3^ Graduate College, Beijing University of Chinese Medicine, Beijing, China; ^4^ Chengdu University of Chinese Medicine, Chengdu, Sichuan, China; ^5^ Institute of Metabolic Diseases, Guang’ anmen Hospital, China Academy of Chinese Medical Sciences, Beijing, China

**Keywords:** asymptomatic hyperuricemia, herbal medicine, randomized controlled trial, network meta-analysis, systematic review

## Abstract

**Background:**

There is still controversy in the medical community about whether asymptomatic hyperuricemia (AH) requires drug treatment. Herbal medicines (HM) are considered a potential intervention for the treatment of hyperuricemia.

**Objectives:**

This study aimed to investigate the efficacy and safety of HM for asymptomatic HUA.

**Materials and Methods:**

A Bayesian network meta-analysis was conducted for patients with asymptomatic HUA in randomized controlled trials identified in PubMed, Web of Science, Cochrane Library, Embase, China National Knowledge Infrastructure (CNKI), Wanfang database, China Biomedical Database (CBM), and China Science and Technology Journal Database (VIP) were searched from their inception to 1 Jan 2025. Outcomes included the serum uric acid (SUA), secondary outcomes (TC, TG, HDL or HDL-C, LDL or LDL-C, and traditional Chinese medicine symptom scores), and adverse events. This study was registered in PROSPERO (CRD42024459357).

**Results:**

All evaluated HM formulations except Lily Plantago Seed tea demonstrated significant SUA reduction versus non drug therapy (NDT), achieving effect sizes spanning from -197.48 (95% CI −249.88 to −145.56) to −14.6 (95% CI −62.09 to 33.31). Probabilistic ranking identified Xuezhikang capsule as the most effective agent for SUA reduction (98.7%), with concurrent improvements in lipid profiles including TC, TG, and HDL levels. Therapeutic benefits extended to TCM symptom scores across all interventions (OR range 3.15–28.44), suggesting broader treatment potential for HUA management. No serious adverse events were documented throughout the trials.

**Conclusion:**

HM interventions demonstrate potential efficacy in managing AH, showing significant reductions in SUA levels alongside beneficial effects on lipid profile modulation and TCM symptom alleviation. While these findings suggest therapeutic promise, the preliminary nature of the evidence necessitates rigorous validation through methodologically robust clinical trials.

**Systematic Review Registration:**

identifier CRD42024459357.

## 1 Introduction

Hyperuricemia (HUA), defined as extracellular fluid urate supersaturation that exceeds its solubility limit in serum, which is approximately 408 mmol/L (6.8 mg/dL) (Dalbeth et al., 2016; FitzGerald et al., 2020), is a metabolic disorder caused by purine metabolism disorders and/or abnormal uric acid excretion. Elevated serum uric acid levels exceeding its saturation in blood and tissue fluids can lead to the formation and deposition of monosodium urate crystals in the joint area, inducing gout. Studies have reported a HUA prevalence of 20.7% among adults in the United States (Kim et al., 2024). In Europe, a cohort study in Ireland noted an increase in HUA prevalence from 20.1% to 24.5% between 2006 and 2014 (Kumar et al., 2018). In Africa, a study in Cameroon showed a prevalence rate of 28.0% for HUA. In Asia, the accurate prevalence of HUA in the Japanese population was 26.8% in male subjects and,9% in female subjects (Tuono et al., 2024). In mainland China, the prevalence of HUA has increased from 11.1% in 2015-16% to 14.0% in 2018-19 (Zhang et al., 2022). Elevated blood uric acid levels are not only associated with gout, but also with the occurrence and development of systemic diseases such as kidney, endocrine metabolism, and cardiovascular and cerebrovascular diseases (Feig et al., 2008; Kawano et al., 2024; Vareldzis et al., 2024). Thus, HUA constitutes a growing threat to the public health.

Although the incidence of hyperuricemia is increasing year by year in the population, AH accounts for the majority of patients, with a prevalence rate of 10.6%–25.8% in the general population (Sapankaew et al., 2022). However, there is still controversy in the medical community regarding whether medication is needed for AH. Some experts believe that although epidemiological studies suggest that hyperuricemia may be associated with cardiovascular, metabolic, and renal complications, the risk/benefit ratio of uric acid lowering therapy is still unclear. Therefore, it is recommended to prioritize non pharmacological interventions such as therapeutic lifestyle changes and adequate exercise (Chalès, 2019; Eleftheriadis et al., 2017; Paul et al., 2017). A contingent of experts maintains a stance of equanimity regarding the pharmacological management of asymptomatic hyperuricemia, positing that the adverse effects associated with urate-lowering therapies are generally minimal. They emphasize the critical importance of delineating the optimal timing for intervention with urate-lowering agents to mitigate the risk of their misuse (Levy and Cheetham, 2015; Valsaraj et al., 2020).In the regions of Japan and China, a cohort of experts advocates for pharmacological intervention in the management of AH (Yamanaka, 2011; Jiang et al., 2021).

There are three types of uric urate-lowering drugs used in clinics: drugs that inhibit urate production, drugs that normalise renal urate excretion, and drugs that catalyse the conversion of urate to the more water soluble and readily excretable allantoin (Dalbeth et al., 2016). Nevertheless, the efficacy of pharmacological treatments for urate-lowering is often deemed inadequate due to the associated adverse reactions. For instance, allopurinol, a widely utilized xanthine oxidase inhibitor, has been reported with the potential for allopurinol hypersensitivity syndrome (Yaseen et al., 2023) Furthermore, benzbromarone, a medication that facilitates uric acid excretion, has faced regulatory restrictions in several countries due to its association with severe hepatotoxicity (Lee et al., 2008). Consequently, the precise therapeutic approaches for the underlying etiology of HUA, the optimal timing for pharmacological intervention, and the mitigation of adverse drug reactions remain subjects of ongoing investigation.

Herbal medicine (HM) has a long history in treating chronic metabolic diseases. Recent empirical studies have shown that Chinese herbal medicines and formulas have the potential to regulate serum uric acid concentration (Chen et al., 2022; Ke et al., 2024; Qian and Shen, 2024), indicating their potential as herbal supplements and alternative therapies for HUA. These findings suggest that that medicinal herbs could serve as viable adjuncts or complementary in the treatment of HUA, exhibiting a promising risk-benefit ratio in comparison to existing therapeutic approaches. Although herbal medicines (HMs) are recognized as potential therapeutic options, they are not without limitations. This systematic review aims to elucidate the potential role of HMs in the treatment of HUA by conducting a network meta-analysis (NMA) to comprehensively synthesize existing evidence and to assess the comparative efficacy of various HMs against placebo or follow-up observation (FO) in randomized clinical trials (RCTs).

## 2 Methods

This study was performed according to the Preferred Reporting Program for Systematic Reviews and Meta-analyses (PRISMAP). Moreover, the protocol of this evaluation is registered with PROSPERO (CRD42024459357).

### 2.1 Search strategies

A comprehensive search was conducted on PubMed, Web of Science, Cochrane Library, Embase, China National Knowledge Infrastructure (CNKI), Wanfang database, China Biomedical Database (CBM), and China Science and Technology Journal Database (VIP). Additionally, the China Clinical Trials Registry (CHiCTR) (www.chictr.org.cn/index.aspx) and ClinicalTrials.gov were searched for ongoing or completed but unpublished RCTs. We included all retrieved RCTs of HM for the treatment of HUA. The search covered the period from the establishment of the databases to 1 January2,025. The search terms used a combination of subject headings and free-text terms, including “hyperuricemia,” “Traditional Chinese Medicine,” “Chinese Herbal Drugs,” ect. No restrictions were applied on language.

### 2.2 Inclusion and exclusion criteria

#### 2.2.1 Participants

The participants have to be prior diagnosed as having HUA according to the American College of Rheumatology guidelines.

#### 2.2.2 Inclusion criteria

The criteria for study inclusion were as follows: 1) patients with HUA as defined by standardized diagnostic manuals or the international classification of the disease, with no restrictions on age, sex, and course of disease; 2) the study population was patients of primary hyperuricemia; and 3) RCTs that compared orally administered herbal medicine versus non-drug treatment (NDT), including placebo and/or FO.

#### 2.2.3 Exclusion criteria

The exclusion criteria were as follows: 1) enrolled participants those administered multi-herbal combinations, except for herbal formulae; 2) Patients with clinical symptoms (such as acute gouty arthritis, gouty stones, uric acid nephropathy) or secondary hyperuricemia (such as drug-induced hyperuricemia); 3) non-random or uncontrolled trials; 4) case reports, expert experiences, and reviews; 5) non-clinical studies, such as animal experiments or pharmacokinetic studies; 6) trials using acupuncture and external Chinese medicines/methods; and 7) studies for which it was not possible to contact the authors for the full text of the article or those with primary outcome data still unavailable after contacting the respective author.

#### 2.2.4 Outcomes

Primary outcome measure was serum uric acid (SUA), Secondary outcome measurements were TC, TG, HDL or HDL-C, LDL or LDL-C, traditional Chinese medicine symptom score (TCMSS). And the outcome of safety was defined as any adverse events during the period of the trial (such as abnormal liver function, renal impairment, gastroin-testinal disorders).

### 2.3 Data extraction and quality assessment

Two scholars (Min and Luo) independently evaluated and validated the quality of the literature using the Cochrane Handbook Randomized Controlled Trial Bias Risk Assessment Scale (Sterne et al., 2019). The evaluation content includes Overall Bias, Selection of the reported result, Measurement of the outcome, Mising outcome data, Deviations from intended interventions and Randomization process. For each field of bias source, the study is classified into three categories: high, low, and some concerns. Discrepancies were resolved by a third reviewer (Zhou et al., 2024).

### 2.4 Data synthesis and analysis

Bayesian network meta-analysis was implemented with the GeMTC package (R v4.5.1), employing a Markov-chain Monte Carlo algorithm across four parallel chains initialized at 4. After 20,000 burn-in iterations, 50,000 additional samples were drawn; convergence was considered adequate when the potential scale reduction factor neared 1, otherwise sampling was extended. Evidence network diagrams were constructed using the network package of STATA 18.0 to visualize the interconnections among interventions. When there was a closed loop, inconsistency between the direct and indirect evidence was detected using node splitting. Employing normal likelihood specifications for continuous biomarkers (SUA, TC, TG, HDL, LDL) (van Valkenhoef et al., 2012) and binomial models for dichotomous TCMSS outcomes (Béliveau et al., 2019). Treatment effects were quantified as mean differences (MD) with 95% confidence intervals (CI) for metabolic parameters and odds ratios (OR) with 95% CI. To assess the degree of heterogeneity, we compared the posterior distribution of the estimated heterogeneity variance with its predictive distribution. Sensitivity analyses were conducted to assess the robustness of the results (Higgins, 2003). Additionally, treatment hierarchies were established using surface under cumulative ranking curve (SUCRA) (Mbuagbaw et al., 2017).

## 3 Results

### 3.1 Study characteristics

**FIGURE 1:**
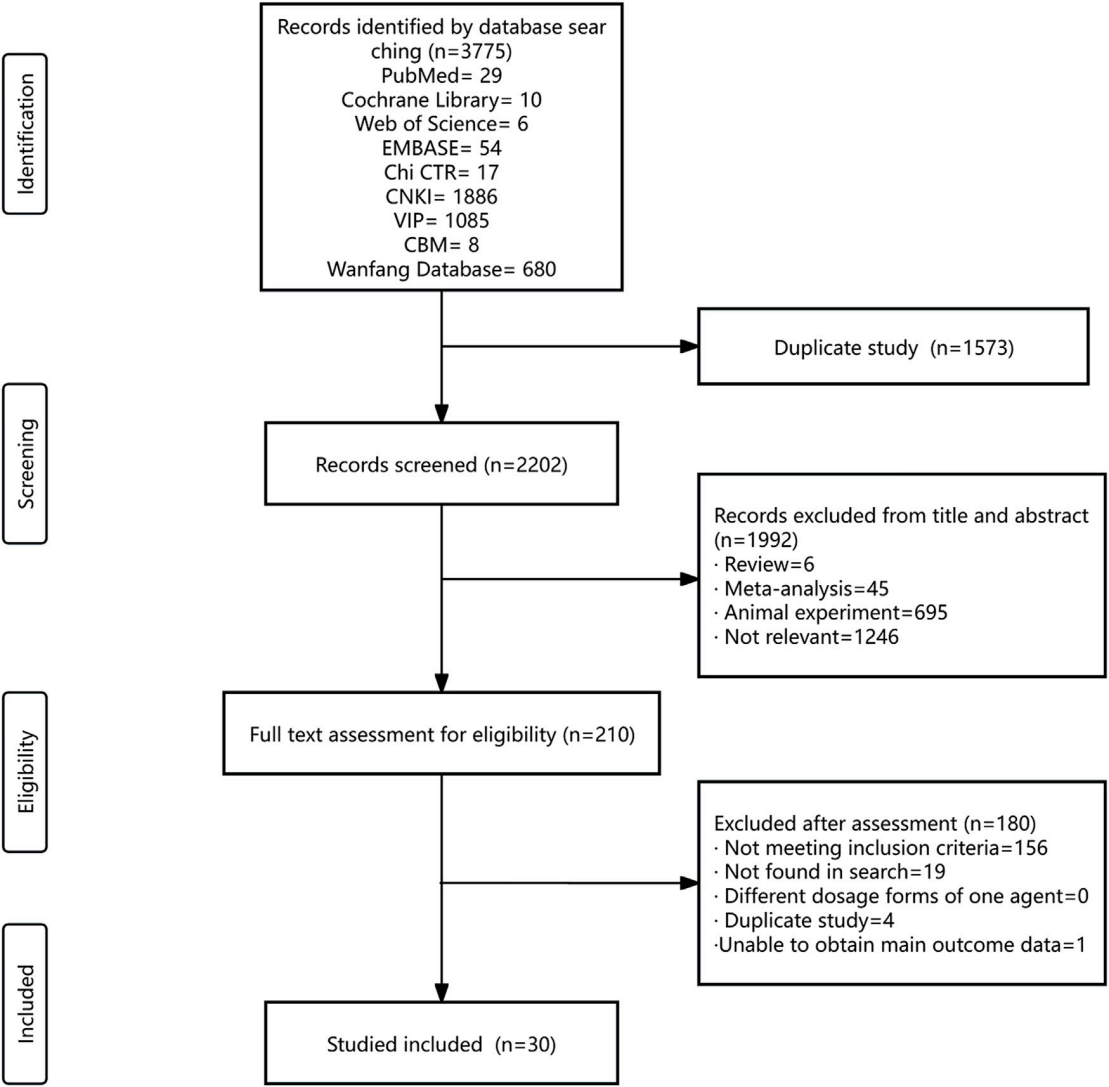
Summary of trial identification and selection.

The systematic literature search encompassed nine databases, yielding a total of 3,775 citations. The databases included PubMed, Cochrane Library, Web of Science, EMBASE, CNKI, VIP, CBM, Chi CTR, and the Wanfang Database. After eliminating duplicate records, 2,202 titles and abstracts were initially screened, leading to the selection of 210 articles for a thorough eligibility assessment. Finally, 30 RCTs were included. ALL RCTs were uniformly conducted within China, spanning the period from 2006 to 2022 (Figure 1).

The 30 trials collectively enrolled 2,532 participants, with 2,443 completing the study from start to finish. The average sample size across the trials was 81 (range, 35-200). The age distribution of the participants spanned from 13 to 94 years. Two studies did not provide gender composition data, while 76.7% of the remaining studies were male. Patients in all studies were diagnosed with AH. The participants in thirteen trials were diagnosed according to the Chinese diagnosis and treatment guidelines or expert consensus for hyperuricemia (Dong, 2022; Qiao and Li, 2016; Sun et al., 2016; Huang et al., 2018; Yu and Zhang, 2018; Lu, 2019; Hao, 2020; Zhang, 2020; Li, 2021; Guo, 2021; Liang, 2021; Zhang et al., 2021; Shao, 2022). The participants in ten trials were diagnosed based on internal medicine or practical medicine and Chinese rheumatology (Liang, 2014; Sun et al., 2006; Chen and Xia, 2010; Zhang et al., 2011; Peng et al., 2012; Zhang et al., 2016a; Zhang et al., 2016b; Huang et al., 2017; Zhang et al., 2017; Liang et al., 2018), while one trial was diagnosed based on the American Rheumatology Society’s classification criteria for gout (Liu, 2017). Six studies satisfied the diagnostic criteria for conditions of indeterminate origin (Chen et al., 2006; Lu and Peng, 2010; Xu et al., 2013; Han, 2015; Liu and Xu, 2017; Shen et al., 2021). Of the 30 RCTs reviewed, 21 studies employed TCM diagnostic patterns as part of their inclusion criteria, and twelve unique TCM diagnostic patterns were included: phlegm dampness, damp heat, spleen deficiency, internal stagnation of dampness and turbidity due to spleen deficiency, dampness heat with blood stasis, qi deficiency with phlegm dampness, dampness toxin heat resistance, spleen and kidney deficiency, dampness and turbidity internal accumulation, dampness heat stagnation in the spleen, spleen and kidney deficiency with dampness and turbidity, and turbid toxin internal accumulation (Supplementary Appendix S1).

A total of 22 Chinese herbal formulas, one Chinese patent medicine, and two single herbal extracts were investigated in 30 trials, and the therapeutic functions of the Chinese Herbal Medicine (HM) formulas aligned with the twelve diagnostic patterns utilized in RCTs, as per TCM theoretical principles. In 30 RCTs, TCM treatment was compared to non-drug therapy. Five of the trials employed a placebo as the control group, while the remaining 25 utilized either a lifestyle intervention or follow-up observation as their control measures. The duration of the trials varied: 8 trials lasted for 4 weeks, 3 trials each were conducted for 30 days, 1 month, and 8 weeks, 2 trials had a 2-month duration, and single trials were conducted for 20 days, 60 days, 90 days, 12 weeks, 16 weeks, 6 months, and 12 months (Supplementary Appendix S2). Plant names were checked utilizing the Medicinal Plant Names Services (http://mpns.kew.org), and the Chinese Pharmacopoeia (https://ydz.chp.org.cn) was used to verify the nomenclature of medicinal fungi, minerals, and animal-derived substances.

### 3.2 Risk-of-bias assessment

**FIGURE 2:**
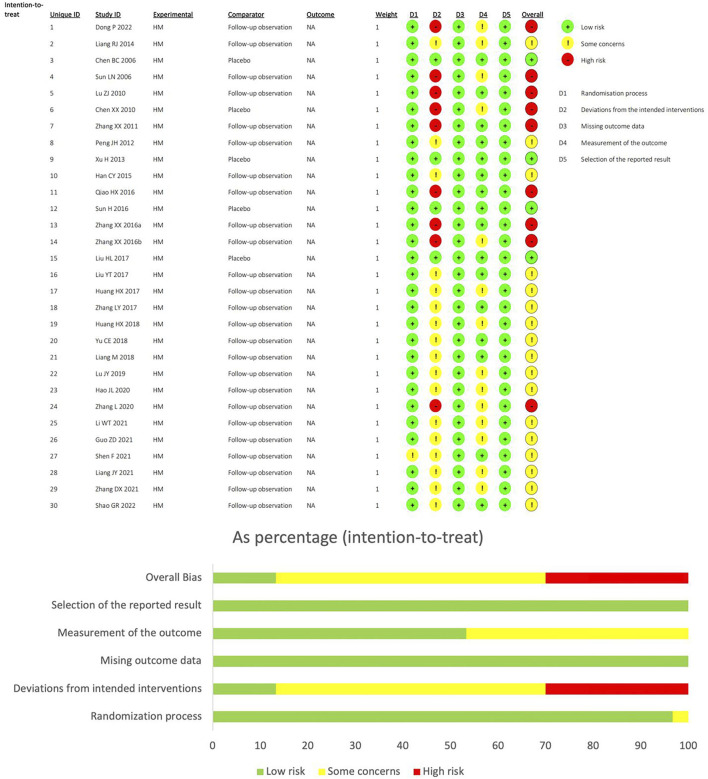
Risk of bias summary and risk of bias graph.

Using the RoB2 tool to assess bias risk, 30.0% of studies were classified as “high-risk”, 56.6% as “somewhat problematic”, and 13.3% as “low-risk”. All study designs were randomized controlled trials, and no high bias risk was observed in any study design. Four studies were double-blind, so they were classified as“low-risk” in the deviations from intended interventions, while the rest were classified as “somewhat problematic” or “high-risk” because the control group was FO. The outcome measures of the 14 studies were influenced by subjective factors, and bias evaluation in outcome measurement was classified as possible risk. In bias evaluation with missing outcome data and Selection of the reported result, all studies were classified as low risk (Figure 2).

### 3.3 Network meta-analysis

**FIGURE 3:**
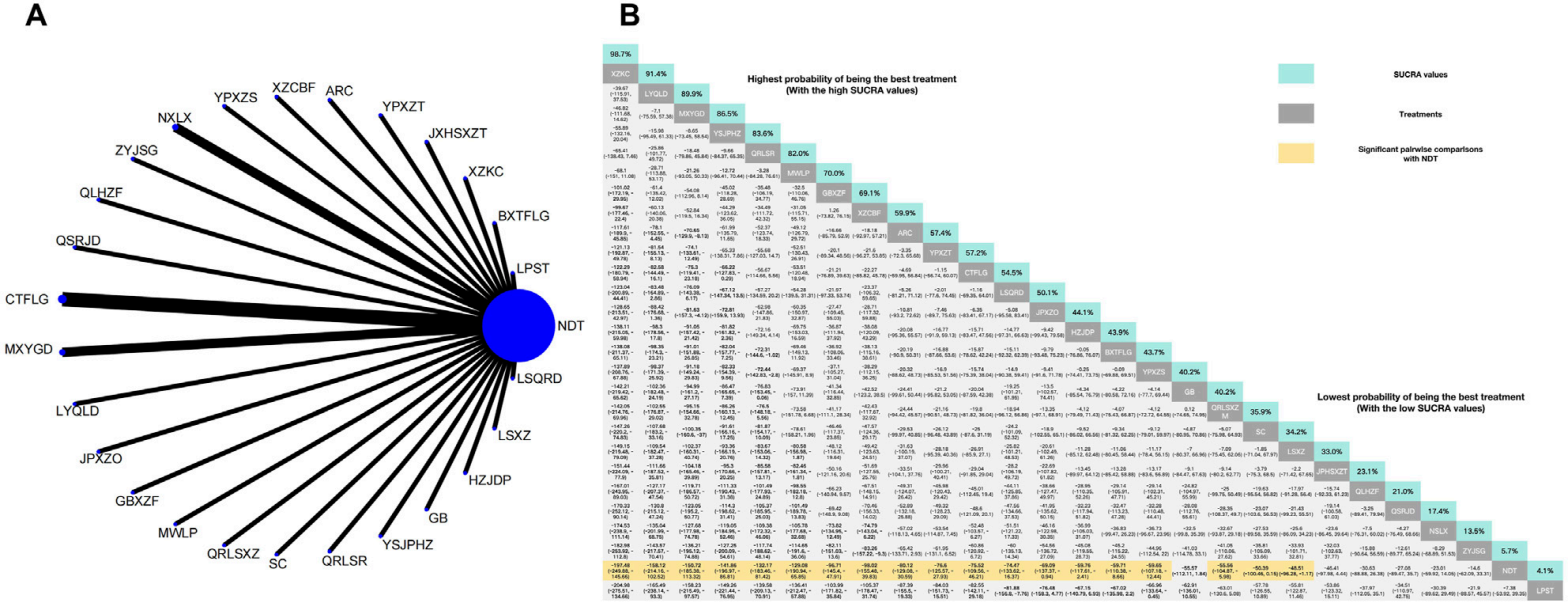
**(A)** Network meta-analysis of available comparisons. **(B)** Legend relative effects size of efficacy at SUA according to NMA. Treatments are ranked according to their chance of being the best treatment. Numbers in the grey boxes are the values of SUCRA, which represents the rank of treatment in SUA. Significant pairwise comparisons with NDT are highlighted in orange. Results with significant differences are bolded.

**FIGURE 4:**
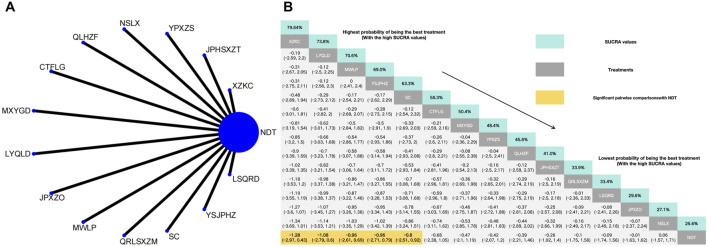
**(A)** Network meta-analysis of available comparisons. **(B)** Legend relative effects size of efficacy at TC according to NMA. Treatments are ranked according to their chance of being the best treatment. Numbers in the grey boxes are the values of SUCRA, which represents the rank of treatment in TC. Significant pairwise comparisons with NDT are highlighted in orange. Results with significant differences are bolded.

**FIGURE 5:**
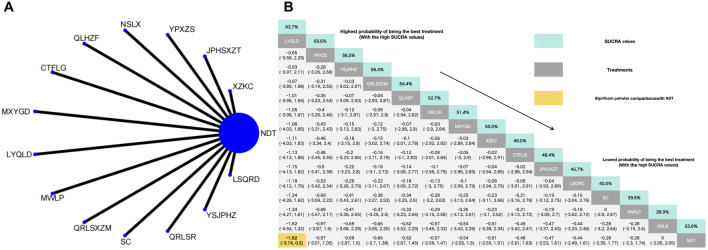
**(A)** Network meta-analysis of available comparisons. **(B)** Legend relative effects size of efficacy at TG according to NMA. Treatments are ranked according to their chance of being the best treatment. Numbers in the grey boxes are the values of SUCRA, which represents the rank of treatment in TG. Significant pairwise comparisons with NDT are highlighted in orange. Results with significant differences are bolded.

**FIGURE 6:**
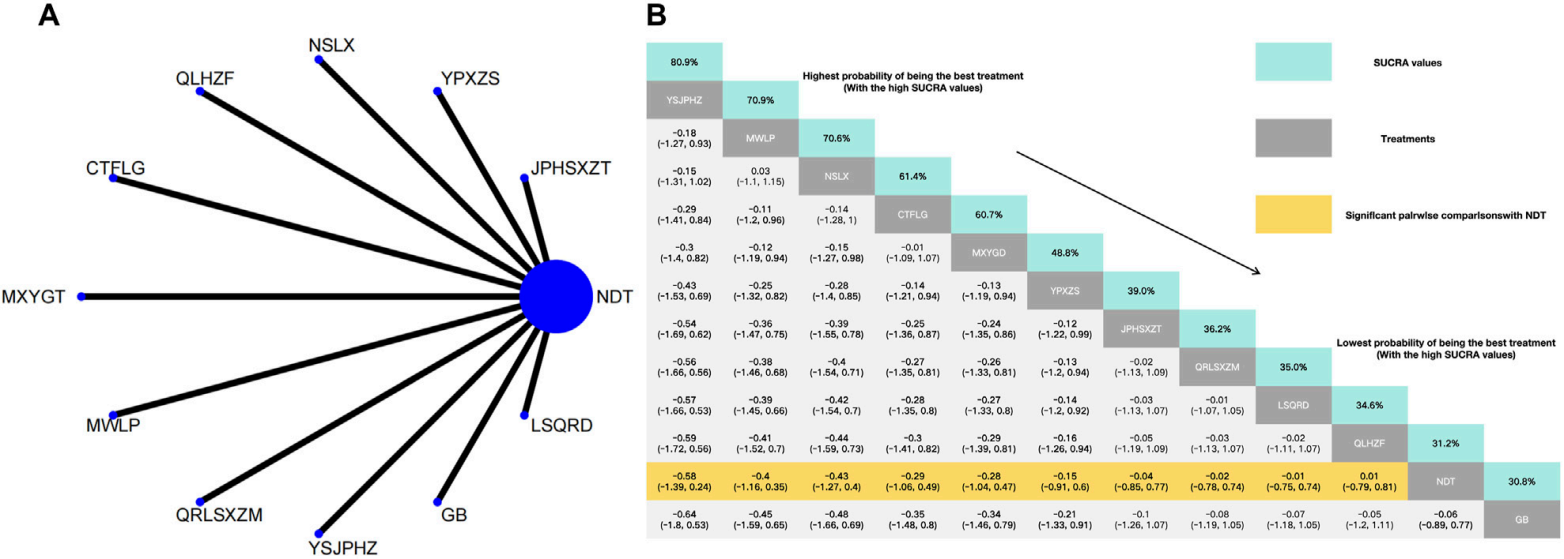
**(A)** Network meta-analysis of available comparisons. **(B)** Legend relative effects size of efficacy at LDL according to NMA. Treatments are ranked according to their chance of being the best treatment. Numbers in the grey boxes are the values of SUCRA, which represents the rank of treatment in LDL. Significant pairwise comparisons with NDT are highlighted in orange. Results with significant differences are bolded.

**FIGURE 7:**
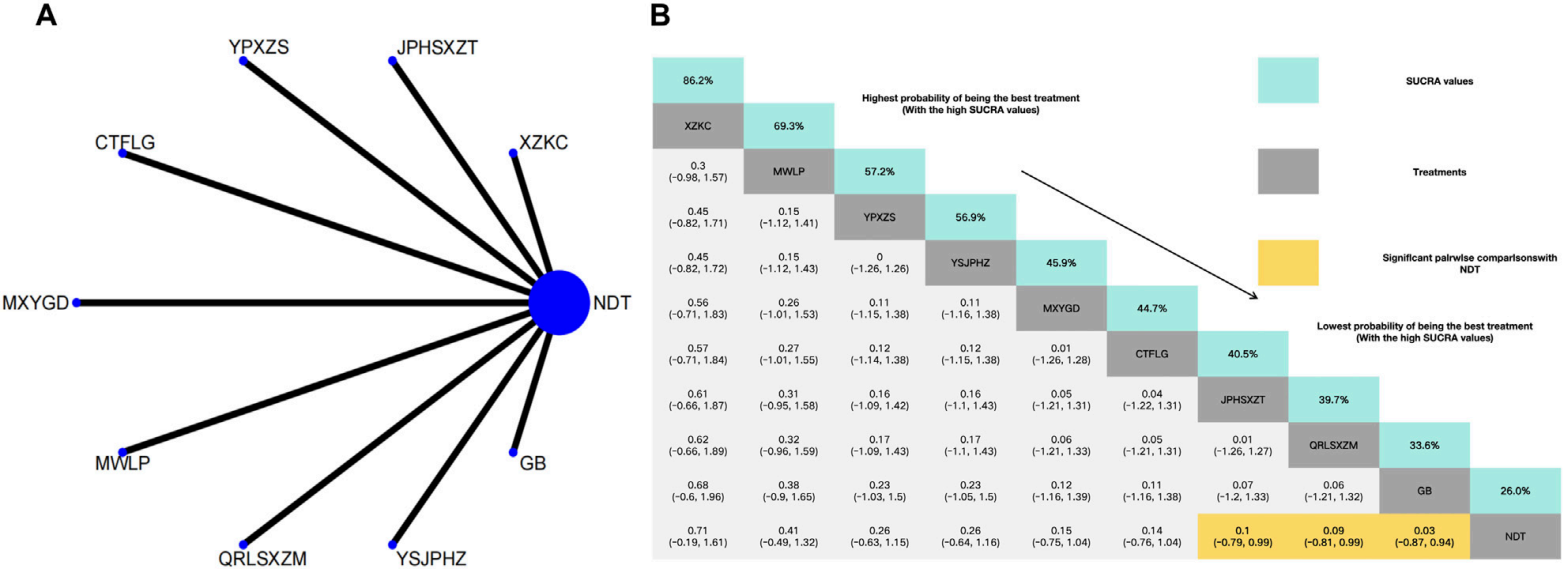
**(A)** Network meta-analysis of available comparisons. **(B)** Legend relative effects size of efficacy at HDL according to NMA. Treatments are ranked according to their chance of being the best treatment. Numbers in the grey boxes are the values of SUCRA, which represents the rank of treatment in HDL. Significant pairwise comparisons with NDT are highlighted in orange. Results with significant differences are bolded.

**FIGURE 8:**
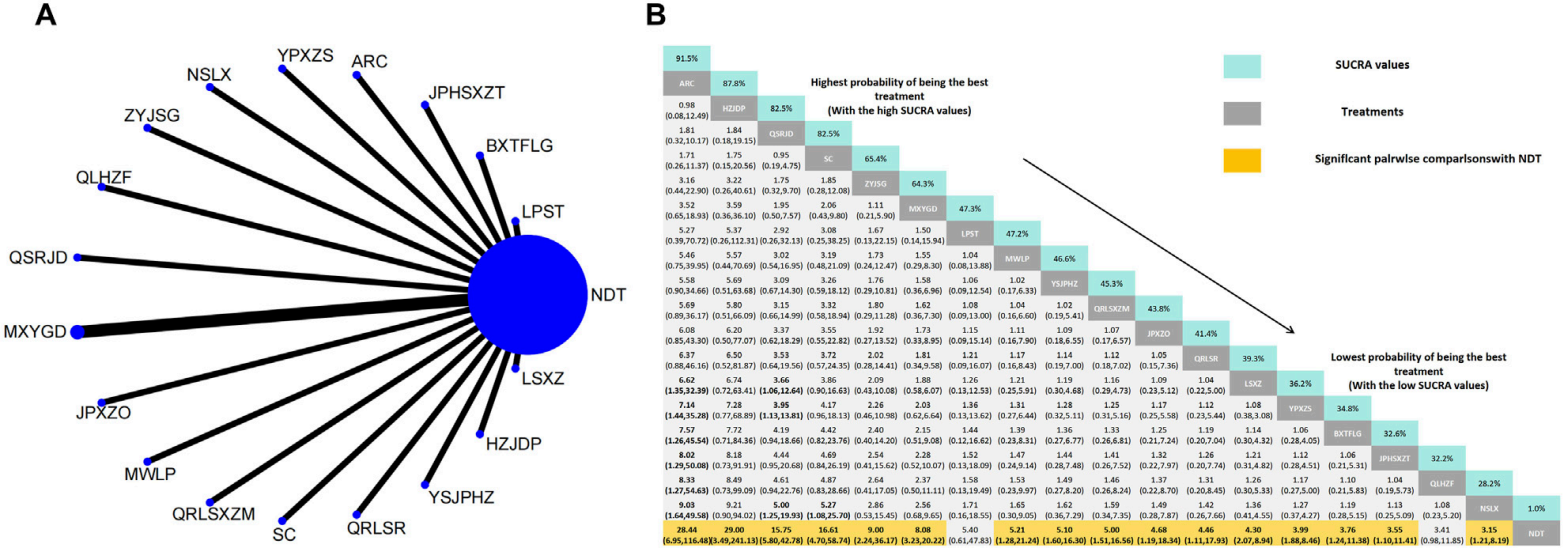
**(A)** Network meta-analysis of available comparisons. **(B)** Legend relative effects size of efficacy at TCMSS according to NMA. Treatments are ranked according to their chance of being the best treatment. Numbers in the grey boxes are the values of SUCRA, which represents the rank of treatment in TCMSS. Significant pairwise comparisons with NDT are highlighted in orange. Results with significant differences are bolded.

Twenty-six herbal intervention measures were used for comparative analysis of SUA, TC, TG, LDL, HDL, and TCMSS, and the network plot and SUCRA value rankings of each intervention measure are shown in Figures 3–8. Supplementary Appendix S3 shows the surface of the cumulative ranking graph.

#### 3.3.1 Reduction of SUA

The NMA of 30 RCTs revealed that 25 herbal interventions outperformed NDT in reducing SUA levels, with Xuezhikang capsule being identified as the most effective. Conversely, LPST was ranked the least effective for SUA reduction. Compared with NDT, Xuezhikang capsule (MD: −197.48, 95% CI: −249.88 to −145.56), Lingyu Qingluo Decoction (MD: 158.12, 95% CI: −214.16 to −102.52), Maxingyigan Decoction (MD: −150.72, 95% CI: −185.38 to −113.32), YiShenJianPiHuaZhuo decoction (MD: −141.86, 95% CI: −196.97 to −86.81), Qingre Lishi Recipe (MD: −132.17, 95% CI: −183.46 to −81.42), Modified Wuling Powder (MD: −129.08, 95% CI: −190.94 to −65.85), Guben Xiezhuo prescription (MD: −96.71, 95% CI: −145.4 to −47.91), Xiezhuo Chubi Formula (MD: −98.02, 95% CI: −155.48 to −39.83), Anoectochilus roxburghii capsule (MD: −80.12, 95% CI: −129。08 to −30.59), Spleen Nourishing and Turbidity Relieving Therapy (MD: −76.6, 95% CI: −125.57 to −27.93), Compound Tufuling Granules (MD: −75.52, 95% CI: −109.56 to −46.21), Lishi Qingre Decoction (MD: −74.47, 95% CI: −133.62 to −27.93), Jianpi Shen Xie Zhuo Ointment (MD: −69.09, 95% CI: −137.37 to −0.94), Huazhuojiedu prescription (MD: −59.76, 95% CI: 117.61 to −2.41), Rhizoma Dioscoreae Hypoglaucae and Glabrous Greenbrier Ghizome Traditional Chinese Medicine granules (MD: −59.71, 95% CI: 110.38 to −8.66), Yunpi xiezhuo Powder (MD: −59.65, 95% CI: 107.18 to −12.44), Green barley (MD: −55.57, 95% CI: 112.11 to 1.84), Qingrelishixiezhuo Method (MD: −55.56, 95% CI: −104.87 to −5.98), Shenchayin (MD: −50.9, 95% CI: −100.46 to 0.15), Substituting tea drinking for dampness-removing and turbidity-resolving (MD: −48.51, 95% CI: −96.28 to −1.17), Strengthening the spleen, dispelling dampness, and relieving turbidity therapy (MD: −46.41, 95% CI: −97.98 to 4.44), Qinluo huazhuo Formula (MD: −30.63, 95% CI: −88.88 to 26.38), TCM formula granules of clear heat evil and detoxification (MD: −27.08, 95% CI: −89.47 to 35.7), TCM uric acid particles (MD: −23.01, 95% CI: −59.92 to 14.05), Zhongyue jiangsangao yaocha (MD: −14.6, 95% CI: −62.09 to 33.31) were better than NDT in reduction in SUA. Furthermore, there was no significant difference between NDT and Lily-Plantago Seed tea (MD: −7.38, 95% CI: −53.92 to 39.35) in their effectiveness in reduction of SUA. The details are shown in Figure 3.

#### 3.3.2 Reduction of TC

The NMA of 14 RCTs revealed that 14 herbal interventions outperformed NDT in reducing TC levels, with Xuezhikang capsule being identified as the most effective. Conversely, NDT was ranked the least effective for TC reduction. Xuezhikang capsule (MD: −1.28, 95% CI: −2.97 to 0.43), Lingyu Qingluo Decoction (MD: −1.08, 95% CI: −2.97 to 0.6), Modified Wuling Powder (MD: −0.96, 95% CI: −2.61 to 0.69), YiShenJianPiHuaZhuo decoction (MD: −0.96, 95% CI: 2.71to 0.79), Shenchayin (MD: −0.8, 95% CI: −2.38 to 1.05) demonstrated a stronger effect in reduction of TC that were superior to the NDT. However, The NMA revealed no significant difference in the reduction of TC between Compound Tufuling Granules (MD: −0.68, 95% CI: −2.38 to 1.05), Maxingyigan Decoction (MD: −0.47, 95% CI: −2.1 to 1.19), Yunpi xiezhuo Powder (MD: −0.42, 95% CI: −2.07 to 1.2), Qinluo huazhuo Formula (MD: −0.39, 95% CI: −2.21 to 1.46), Strengthening the spleen, dispelling dampness, and relieving turbidity therapy (MD: −0.26, 95% CI: −1.92 to 1.4), Qingrelishixiezhuo Method (MD: −0.1, 95% CI: −1.75 to 1.56), Lishi Qingre Decoction (MD: −0.09, 95% CI: −1.74 to 1.56), Jianpi Shen Xie Zhuo Ointment (MD: −0.01, 95% CI: −1.63 to 1.62), TCM uric acid particles (MD: 0.06, 95% CI: −1.57–1.71), and NDT (Figure 4).

#### 3.3.3 Reduction of TG

In the NMA of 14 RCTs, Lingyu Qingluo Decoction was ranked the best for reduction of TG, while NDT was ranked worst for reduction of TG. Compared with NDT, Lingyu Qingluo Decoction (MD: −1.62, 95% CI: −3.74 to 0.5) had a superior effect in reduction of TG. Yunpi xiezhuo Powder (MD: −0.97, 95% CI: −3.01 to 1.05), YiShenJianPiHuaZhuo decoction (MD: −0.69, 95% CI: −2.87 to 1.5), Qingrelishixiezhuo Method (MD: −0.66, 95% CI: −2.7 to 1.38), Qinluo huazhuo Formula (MD: −0.62, 95% CI: −2.67 to 1.38), Qingre Lishi Recipe (MD: −0.57, 95% CI: −2.59 to 1.47), Maxingyigan Decoction (MD: −0.54, 95% CI: −2.55 to 1.5), Xuezhikang capsule (MD: −0.51, 95% CI: −2.55 to 1.61), Compound Tufuling Granules (MD: −0.49, 95% CI: −2.61 to 1.63), Strengthening the spleen, dispelling dampness, and relieving turbidity therapy (MD: −0.47, 95% CI: −2.53 to 1.61), Lishi Qingre Decoction (MD: −0.44, 95% CI: −2.49 to 1.61), Shenchayin (MD: −0.28, 95% CI: 0.-2.35 to 1.77), Modified Wuling Powder (MD: −0.28, 95% CI: −2.3 to 1.74), TCM uric acid particles (MD: 0, 95% CI: −2.65 to 2.05) were all found to reduce TG. However, there was no statistically significant difference in efficacy compared to NDT for any of these interventions (Figure 5).

#### 3.3.4 Reduction of LDL

In the NMA of 11 RCTs, YiShenJianPiHuaZhuo decoction was ranked the best for reduction LDL, and Green barley was ranked the worst for reduction LDL. Compared with NDT, YiShenJianPiHuaZhuo decoction (MD: −0.58, 95% CI: −1.39 to 0.24), Modified Wuling Powder (MD: −0.4, 95% CI: −1.16 to 0.35), TCM uric acid particles (MD: −0.43, 95% CI: −1.27 to 0.4), Compound Tufuling Granules (MD: −0.29, 95% CI: −1.06 to 0.49), Maxingyigan Decoction (MD: −0.28, 95% CI: −1.04 to 0.47), Yunpi xiezhuo Powder (MD: −0.15, 95% CI: −0.91 to 0.6), Strengthening the spleen, dispelling dampness, and relieving turbidity therapy (MD: −0.04, 95% CI: −0.85 to 0.77), Qingrelishixiezhuo Method (MD: −0.02, 95% CI: −0.78 to 0.74), Lishi Qingre Decoction (MD: −0.01, 95% CI: −0.75 to 0.74), Qinluo huazhuo Formula (MD: 0.01, 95% CI: −0.79–0.81) had a superior effect in reduction of LDL. In addition, and NDT (MD: −0.06, 95% CI: −0.89 to 0.77) exhibited no significant differences compared with Green barley (Figure 6).

#### 3.3.5 Effects of HDL

Xuezhikang capsule was identified as the most effective intervention for increasing HDL levels, whereas NDT was rated the least effective for the same outcome. Compared with NDT, Xuezhikang capsule (MD: 0.71, 95% CI: −0.19–1.61), Modified Wuling Powder (MD: 0.41, 95% CI: −0.49–1.32), Yunpi xiezhuo Powder (MD: 0.26, 95% CI: −0.63–1.15), YiShenJianPiHuaZhuo decoction (MD: 0.26, 95% CI: −0,64 to 1.16), Maxingyigan Decoction (MD: 0.15, 95% CI: −0.75–1.04), Compound Tufuling Granules (MD: 0.14, 95% CI: −0.76–1.04) can slightly increase HDL but not statistically significantly. Interestingly, Strengthening the spleen, dispelling dampness, and relieving turbidity therapy (MD: 0.1, 95% CI: −0.79–0.99), Qingrelishixiezhuo Method (MD: 0.09, 95% CI: −0.81–0.99) and Green barley (MD: 0.03, 95%CI: −0.87–0.94) exhibited significant differences compared with the NDT (Figure 7).

#### 3.3.6 Efficacy of TCMSS

In a comparison of TCM symptom scores across eighteen RCTs, the efficacy was found to be superior to that of NDT. Anoectochilus roxburghii capsule were ranked as the most effective TCMSS. TCM uric acid particles ranked worst in terms of the efficacy of TCMSS, which was still superior to NDT. Anoectochilus roxburghii capsule (OR:28.44, 95%CI: 6.95–116.48), Huazhuojiedu prescription (OR:29.00, 95%CI: 3.49–241.13), TCM formula granules of clear heat evil and detoxification (OR:15.75, 95%CI: 5.80–42.78), Shenchayin (OR:16.61, 95%CI: 4.70–58.74), Zhongyue jiangsangao yaocha (OR: 9.00, 95%CI: 2.24–36.17), Maxingyigan Decoction (OR: 8.08, 95%CI: 3.23–20.22), Modified Wuling Powder (OR: 5.21, 95%CI: 1.28–21.24), YiShenJianPiHuaZhuo decoction (OR: 5.10, 95%CI: 1.60–16.30), Qingrelishixiezhuo Method (OR: 5.00, 95%CI: 1.51–16.56), Jianpi Shen Xie Zhuo Ointment (OR: 4.68, 95%CI: 1.19–18.34), Qingre Lishi Recipe (OR: 4.46, 95%CI: 1.11–17.93), Substituting tea drinking for dampness-removing and turbidity-resolving (OR: 4.30, 95%CI: 2.07–8.94), Yunpi xiezhuo Powder (OR: 3.99, 95%CI: 1.88–8.46), Rhizoma Dioscoreae Hypoglaucae and Glabrous Greenbrier Ghizome Traditional Chinese Medicine granules (OR: 3.76, 95%CI: 1.24–11.38), Strengthening the spleen, dispelling dampness, and relieving turbidity therapy (OR: 3.55, 95%CI: 1.10–11.41) and TCM uric acid particles (OR: 3.15, 95%CI: 1.21–8.19) demonstrated greater efficacy than NDT. While compared with NDT, both Lily-Plantago Seed tea (OR: 5.40, 95%CI: 0.61–47.83) and Qinluo huazhuo Formula (OR: 3.41, 95%CI: 0.98–11.85) demonstrated no statistically significant differences in efficacy, despite a slight superiority in effectiveness over NDT (Figure 8).

### 3.4 Inconsistency and heterogeneity

The heterogeneities of SUA, TC, TG, LDL, HDL, and TCMSS were calculated (Supplementary Appendix S4). Thirty clinical trials demonstrated consistent SUA reduction in HUA patients receiving HM therapy. Fixed-effects analysis identified substantial between-study heterogeneity (I^2^ = 96%, P < 0.00001). Comparative analysis showed superior efficacy of HM intervention over NDT, with mean SUA levels decreasing by 42.23 μmol/L (95% CI: 39.16 to 45.30, Z = 26.98, P < 0.00001). Fourteen clinical trials demonstrated consistent TC reduction in HUA patients receiving HM therapy. Fixed-effects analysis identified substantial between-study heterogeneity (I^2^ = 54%, P = 0.008). Comparative analysis showed superior efficacy of HM intervention over NDT, with mean TC levels decreasing by 0.34 mmol/L (95% CI: 0.24 to 0.43, Z = 6.93, P < 0.00001). Fourteen clinical trials demonstrated significant TG reduction in HUA patients receiving HM therapy. Fixed-effects analysis identified substantial between-study heterogeneity (I^2^ = 50%, P = 0.02). Comparative analysis showed superior efficacy of HM intervention over NDT, with mean TG levels decreasing by 0.52 mmol/L (95% CI: 0.43 to 0.61, Z = 11.75, P < 0.00001). Eleven clinical trials demonstrated LDL reduction in HUA patients receiving HM therapy. Fixed-effects analysis identified substantial between-study heterogeneity (I^2^ = 59%, P = 0.006). Comparative analysis showed superior efficacy of HM intervention over NDT, with mean TG levels decreasing by 0.20 mmol/L (95% CI: 0.12 to 0.28, Z = 4.75, P < 0.00001). The effect of HDL in patients with HUA treated with HM was reported in 9 studies. Significant heterogeneity was observed among the studies (P < 0.00001, I^2^ = 92%). The fixed effect model combining effect size was used for analysis, with HM treatment, HDL was significantly higher than that of the NDT group (MD = -0.19, 95% CI: 0.23 to −0.14, Z = 8.56, P < 0.0001). Nineteen clinical trials demonstrated TCMSS improvement in HUA patients receiving HM therapy. Fixed-effects analysis identified substantial between-study heterogeneity (I^2^ = 80%, P < 0.00001). Comparative analysis showed superior efficacy of HM intervention over NDT, with HM treatment, the efficacy of TCMSS was significantly higher than that of the NDT group (OR = 0.66, 95% CI:0.62 to 0.70, Z = 13.36, P < 0.0001).

### 3.5 Adverse events

None of the RCTs (Supplementary Appendix S1) reported any serious adverse events. Three studies reported adverse reactions associated with both HM andNDT, while one study solely reported adverse reactions linked to HM, and another study exclusively reported adverse reactions pertaining to NDT. An RCT focusing on Strengthening the spleen, dispelling dampness, and relieving turbidity therapy reported two cases of adverse events (n = 2) exclusively in the NDT group (Sun et al., 2006). These adverse events were related to acute gout attacks. Separately, an RCT on Traditional Chinese Medicine (TCM) formula granules designed to clear heat and detoxify (Sun et al., 2016) and another RCT on TCM uric acid particles (Xu et al., 2013) each documented two cases of mild adverse events, which included lower back pain and stomach discomfort. Notably, an additional case of oral ulcer was reported in the study on TCM uric acid particles. In the HM group of the Maxingyigan Decoction RCT (Huang et al., 2018), there were two recorded cases of mild adverse events: one case of stomach discomfort (n = 1) and one case of abnormal transaminase levels (n = 1). Furthermore, the RCT on Anoectochilus roxburghii capsule (Chen and Xia, 2010) reported two cases of mild elevation in transaminase levels.

### 3.6 Consistency tests

The previous results indicate that there is no closed loop between these intervention measures, so there is no need for consistency testing.

### 3.7 Model convergence evaluation

In the Bayesian model constructed in this study, each Markov chain in the trace diagrams of the SUA (an outcome index) in the iterative process (Supplementary Appendix S5) reached stable convergence from the beginning. In addition, the overlap in the subsequent calculations accounted for most of the fluctuation in the chain, without no fluctuations in a single chain identifiable by the naked eye, indicating satisfactory convergence. Densities followed approximately normal distributions, with small preset bandwidths, indicating satisfactory model convergence. The Gelman-Rubin-Brooks plot (Supplementary Appendix S6) showed that after 20,000 preiterations, the median and 97.fifth-percentile value of the shrink factor rapidly approached 1 and stabilized, with a PSRF of 1, indicating satisfactory model convergence. The results on model convergence for the other outcome indices are provided in Supplementary Appendixs S4, S5. The results all suggest satisfactory model convergence and sufficient iterations. Therefore, the Bayesian model constructed in this study can effectively predict the posterior distribution of the parameters.

### 3.8 Publication bias

Comparison-adjusted funnel plots were drawn to assess the publication bias of each outcome indicator (Supplementary Appendix S7). Each point on the funnel plots represents an RCT. The points in Figure A are asymmetrically distributed, indicating that the SUA results may include publication bias.The points in other Figures are roughly symmetrically distributed, indicating that the likelihood of publication bias in the TC, TG, LDL, HDL, and TCMSS was small.

## 4 Discussion

### 4.1 Summary of findings

The paired meta-analysis demonstrated that HMs were more effective than placebo in reducing SUA levels. Furthermore, NMAs showed that specific formulations, including Xuezhikang Capsule, Lingyu Qingluo Decoction, and Maxingyigan Decoction, may exhibit significant efficacy in lowering SUA levels. HM additionally demonstrated superiority over placebo in alleviating symptoms of Asymptomatic HUA patients, with notable clinical relevance. Moreover, compared to placebo, HM may significantly impact lipid metabolism by elevating HDL levels and decreasing LDL levels. It also exhibited greater efficacy than placebo in regulating TC and TG levels.

### 4.2 Implications for clinical therapy

#### 4.2.1 Role of herbal medicine in patients with HUA

To the best of our knowledge, this represents one of the first evidence syntheses comparing herbal remedies for the treatment of AH using a Bayesian network meta-analysis. Our findings substantiate that herbal remedies demonstrate substantial efficacy in lowering serum uric acid concentrations relative to baseline levels. The NMA results of HUA patients showed that compared with the control intervention, almost all HMs could significantly reduce abnormally elevated serum uric acid levels, with Xuezhikang capsule, Lingyu Qingluo Decoction, and Maxingyigan Decoction showing the most significant reduction in uric acid levels. Interestingly, The HM therapies reported are all based on the principle of dispelling dampness, with high-frequency use of herbs such as Smilax glabra Roxb (Tufuling), Coix seed (Yiyiren), Dioscorea collettii var. hypoglauca (Palib.) S.J.Pei & C.T.Ting (Bixie), and Atractylodes macrocephala Koidz (Baizhu). According to reports, the total flavonoids of Smilax glabra Roxb (Tufuling) can significantly reduce uric acid in hyperuricemia mice by inhibiting XOD activity and upregulating the expression of organic anion transporter 1 (OAT1), organic cation/carnitine transporter 2 (OCTN2), and their mRNA in renal tissue (Huang et al., 2018). The ethanol extract of Coix seed (Yiyiren) significantly reduced the serum uric acid and creatinine levels in mice, and had a certain improvement effect on mouse kidney damage caused by high uric acid (Sui Y, 2023). The saponins extracted from D. collettii (Bixie) facilitate the re-absorption of uric acid and decrease the secretion of serum uric acid by modulating the expression of various transporters. Specifically, they upregulate glucose transporter 9 (GLUT9) and renal urate transporter 1 (URAT1), while downregulating the expression levels of organic anion transporter 1 (OAT1) and organic anion transporter 3 (OAT3). Furthermore, uric acid excretion is increased in a manner that is dependent on the dose of the saponins (Zhu et al., 2017). Collectively, these findings, alongside existing preclinical and clinical evidence, provide preliminary support for the potential efficacy of selected herbs in reducing SUA levels, though further validation in larger, well-designed studies is warranted.

#### 4.2.2 Potential role of herbal medicine in regulating lipid function

This study confirms that, compared to pre-treatment levels, herbal medicine has demonstrated significant efficacy in regulating hyperuricemia accompanied by lipid metabolism disorders. The relationship between SUA and dyslipidemia is intricate and remains incompletely understood. Nevertheless, numerous studies have indicated an association between elevated SUA levels and dyslipidemia. Two studies utilizing data from the National Health and Nutrition Examination Survey (NHANES) have revealed a significant positive correlation between serum TC, TG, LDL cholesterol levels, and serum uric acid levels in American adults. Conversely, serum HDL cholesterol levels exhibited a negative correlation with serum uric acid levels (Peng et al., 2015; Zhou et al., 2024). Recent cross-sectional studies conducted within the Chinese Mainland have demonstrated a significant association between dyslipidemia and hyperuricemia. The likelihood of hyperuricemia is significantly positively correlated with elevated levels of TG and TC, while an inverse correlation is observed with HDL levels (Xu et al., 2014; Cui et al., 2020; Fang et al., 2024). Current research shows that, HUA can increase the activity of fructokinase in fructose metabolism, enhance TG synthesis, and activate NADPH oxidase that induces oxidative stress, producing lipid peroxidation and inflammatory mediators (Yin et al., 2017). Furthermore, uric acid induces lipid metabolic disturbances through lysophosphatidylcholine acyltransferases (LPCAT)-3 mediated p- Signal transducer and activator of transcription (STAT)-3 inhibition and SREBP-1c activation (Liu et al., 2020). The mechanisms by which traditional Chinese medicine reduces blood lipids primarily involve inhibiting exogenous lipid absorption, suppressing endogenous lipid synthesis, enhancing bile acid secretion and excretion, preventing lipid peroxidation, elevating HDL-C levels, reducing insulin resistance, and modulating intestinal flora homeostasis (Li et al., 2021). Salvia miltiorrhiza Bunge (Danshen) contains various active metabolites, such as tanshinone I, tanshinone IIA, cryptotanshinone, which can inhibit fat production by reducing the levels of peroxisome proliferator activated receptor (PPAR)-γ and fatty acid synthase (FASN) (Yang et al., 2024). Emodin, a natural anthraquinone derivative found in Rheum palmatum L. (Dahuang), can enhance the activity of brown adipose tissue (BAT), accelerate the transport and consumption of fatty acids, reduce the generation and accumulation of fat in the liver, and improve lipid metabolism disorders (Cheng et al., 2021; Tzeng et al., 2012). At present, there is sufficient evidence to prove that herbs can improve lipid metabolism disorders. However, whether these herbs can improve lipid metabolism disorders while reducing uric acid requires further modern pharmacology to confirm their effects and mechanisms.

#### 4.2.3 Advantages of herbal medicine in alleviating symptoms

At present, there is still a lack of clear consensus on the definition of AH, with some scholars defining it as elevated serum uric acid levels (typically >6.8 mg/dL) without clinical manifestations of gout (Yip et al., 2020). Although AH is mainly characterized by elevated blood uric acid levels in modern medicine and lacks obvious clinical symptoms, from the perspective of traditional Chinese medicine, asymptomatic does not mean complete internal balance of the body. Traditional Chinese Medicine (TCM) embraces a holistic approach as its core principle. Through the four diagnostic methods—observation, auscultation and olfaction, inquiry, and palpation—TCM practitioners can discern internal imbalances in AH patients. These imbalances may manifest as specific syndrome types, such as damp heat syndrome, spleen deficiency, and phlegm-dampness. These symptoms not only indicate functional disturbances within the patient’s body but may also serve as the underlying factors contributing to the elevation of uric acid levels. Herbal therapy alleviates the syndromes identified in TCM, which not only aids in balancing the overall functional status of patients but may also indirectly diminish the risk of complications associated with hyperuricemia. However, the number of clinical studies on herbal treatment for AH is limited, and the quality of research design as well as the completeness of follow-up data are inadequate. These limitations somewhat obscure the clarity of the causal relationship between the improvement of TCM syndromes through herbal therapy and the reduction of uric acid levels.

#### 4.2.4 Potential advantages of herbal medicine

Currently, the necessity of pharmacological intervention for AH remains controversial (Chalès, 2019; Paul et al., 2017; Yip et al., 2020). Conventional treatments include xanthine oxidase inhibitors to reduce uric acid production, uricosuric agents to promote uric acid excretion, and uricase-based therapies to enhance uric acid metabolism (Dalbeth et al., 2016; Du et al., 2024). While these therapies demonstrate efficacy in lowering serum uric acid levels, they are associated with potential adverse effects such as hepatotoxicity, renal burden, and drug interactions (Du et al., 2024). In contrast, herbal therapy for hyperuricemia has a long - standing history, with definite therapeutic effects and minimal side effects, making it a highly - regarded alternative therapy. Studies have shown that herbs can effectively reduce serum uric acid levels through multiple pathways and targets (Bai et al., 2024), while also alleviating TCM syndromes associated with hyperuricemia, such as damp - heat syndrome (Yu et al., 2018). Lychnophora trichocarpha, a traditional medicine in Brazil, used in Brazilian folk medicine to treat pain, bruise, rheumatism, and inflammatory diseases,its ethanol extracts and ethyl acetate fraction can exert anti-hyperuricemia activity through XOD inhibition, and can also reduce monosodium urate crystals induced paw edema in mice (De Souza et al., 2012). A meta-analysis of 11 RCTs studies involving 845 HUA patients showed that the clinical effect of TCM combined with febuxostat in the treatment of hyperuricemia was better than that of febuxostat alone, which could significantly reduce the level of serum uric acid and the incidence of adverse events (Pan and Liu, 2024). However, the recurrence rate of HUA was not described in the RCT study we included, so we could not further analyze the data. Hyperuricemia is prone to relapse due to the influence of high purine diet, fructose and alcohol intake. If the level of uric acid cannot be controlled in time, it may induce gout and other diseases, causing serious health and psychological burden to patients. Therefore, even if the patient’s uric acid level returns to normal, it also needs to be followed up in time. We recommend more follow-up in HUA clinical trials to better evaluate the efficacy and safety of drugs. If future studies provide sufficient evidence supporting the ability of herbal medicine to reduce AH recurrence rates, its application in HUA management might be further explored.

### 4.3 Strengths and limitations

The main strengths of this study are that we systematically integrated existing research, analyzed the effectiveness of different HM and placebo/FO on HUA patients, and used a network meta-analysis method to evaluate their impact on HUA related indicators. In addition, literature searches and screenings were conducted across multiple electronic databases to ensure comprehensiveness. Our NMA results will provide unique insights into HM localization, and we hope that these findings will enhance the collaborative decision-making process by offering evidence-based guidance for optimizing patient care.

However, our study also has some limitations that must be addressed. First, the clinical trials included in this study all took place in Chinese Mainland, and the HM therapy used was mainly based on traditional Chinese medicine. The efficacy of herbal medicine in other countries and regions was not evaluated. Secondly, although all included trials have undergone rigorous review, there are also some differences in the course of HUA disease, follow-up time of each study, and the range of detection indicators in each group, which may lead to heterogeneity in the results. We believe there may be several reasons for this: 1) Approximately 83% (25/30) of included trials used lifestyle interventions or follow-up observation as comparators instead of placebo. This design limitation impedes blinding, potentially inflating perceived efficacy due to performance and detection bias; 2) In the included studies, the renal function status of AH patients is unknown, and there is no report on whether the patients have had gout attacks in the past. Different disease stages and severity lead to significant differences in SUA. Thirdly, the included studies have issues such as small sample sizes and lack of reporting on random methods, which may lead to biased research results. In addition, the inclusion of heterogeneous dosage forms (e.g., decoctions, capsules, ointments) with divergent pharmacokinetic profiles may violate the transitivity assumption essential for valid indirect comparisons, potentially confounding efficacy rankings. Future RCTs should prioritize standardized preparations (e.g., fixed-dose granules) to minimize compositional variability and enhance evidence reliability. Despite males comprising 76.7% of participants, the lack of sex-stratified efficacy data in original trials prevented subgroup analysis. Future studies should report outcomes by sex.Therefore, large sample, multicenter, prospective randomized controlled trials are needed to compare the clinical efficacy and safety of placebo and FO, in order to clarify whether herbal intervention is necessary for AH.

## 5 Conclusion

Despite limitations, this study represents the first network meta-analysis reviewing multiple herbal medicines for AH. Preliminary evidence suggests potential SUA-lowering effects of certain HMs and offered additional benefits in lipid metabolism regulation and TCM symptom score improvement. However, the efficacy of HM interventions across different stages of hyperuricemia (including AH, gout and uric acid nephropathy) requires further observation to clarify clinical medication strategies. Future research should include large-scale placebo-controlled trials to validate the efficacy, safety, tolerability, and recurrence rates of herbal treatments for hyperuricemia.

## Data Availability

The original contributions presented in the study are included in the article/Supplementary Material, further inquiries can be directed to the corresponding authors.
