# Supplementary material for: Herbal medicine for asymptomatic hyperuricemia: a systematic review and network meta-analysis
Source: Front Pharmacol. 2025 Sep 29;16:1627714. doi: 10.3389/fphar.2025.1627714 (PMC12515837; doi:10.3389/fphar.2025.1627714)
Supplement: Supplementary file 1 [file Supplementaryfile5.pdf]

Trace plot and density plot for the ratio of SUA

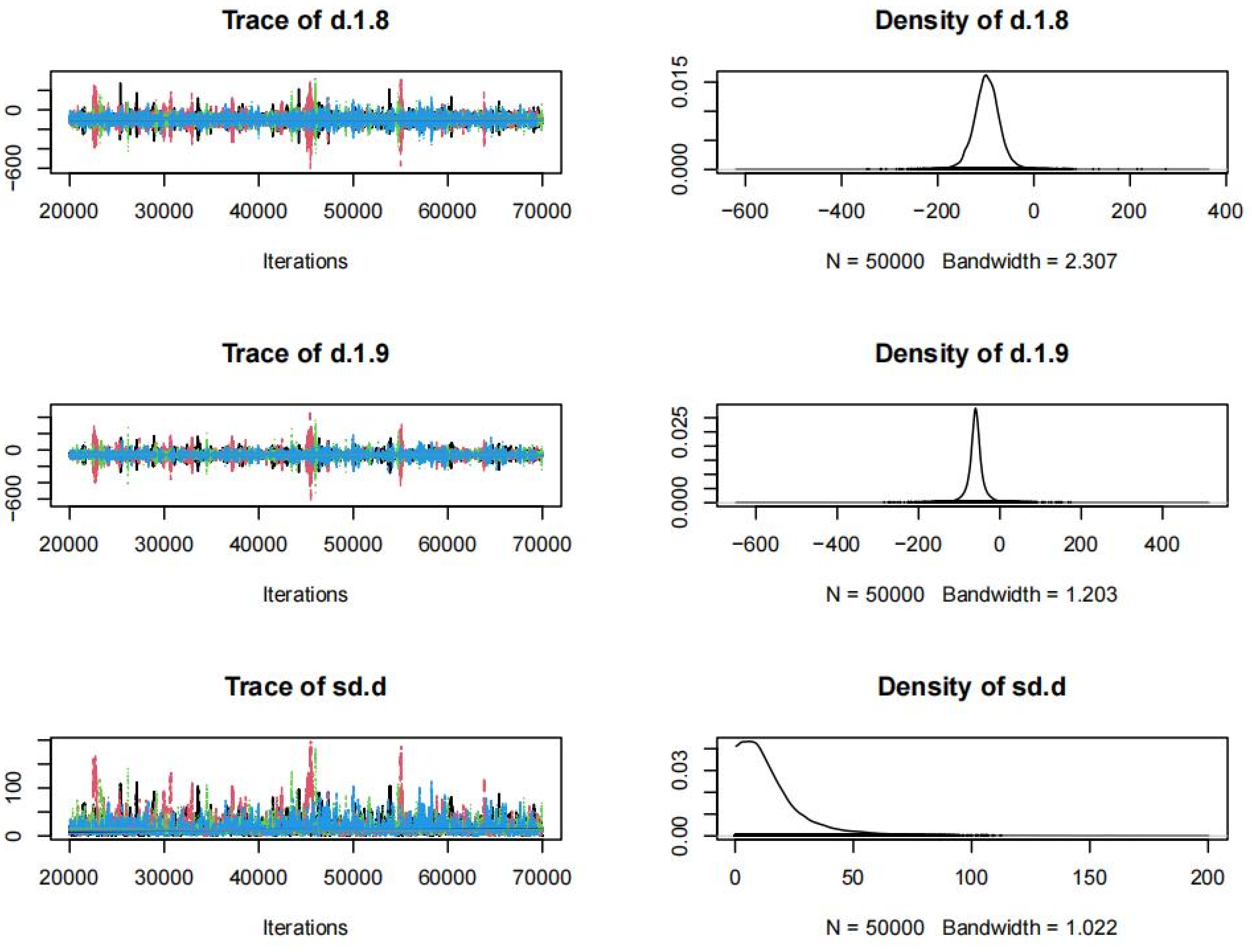

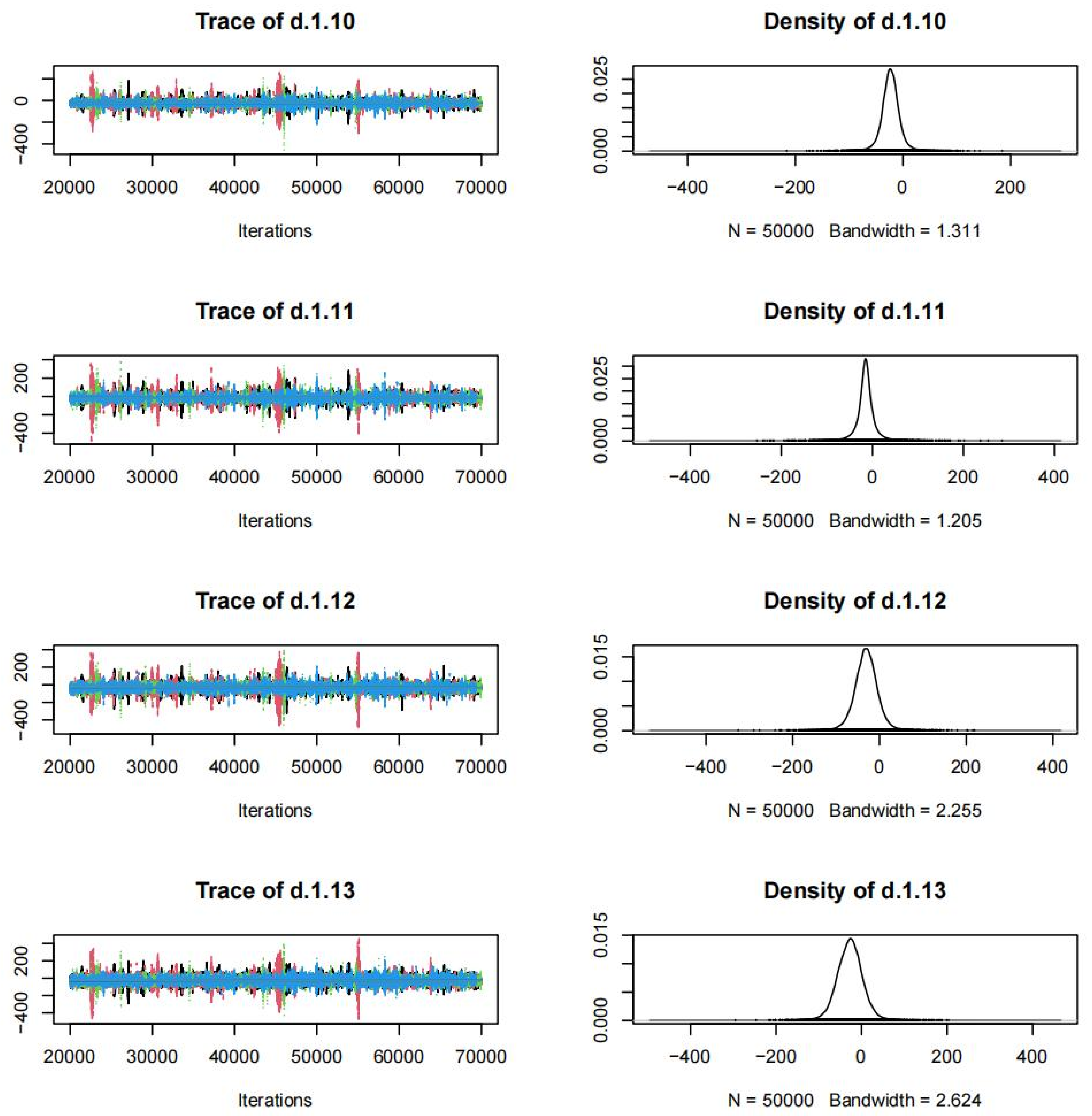
Trace of d.1.10
Iterations
Density of d.1.10
N = 50000 Bandwidth = 1.311
Trace of d.1.11
Iterations
Density of d.1.11
N = 50000 Bandwidth = 1.205
Trace of d.1.12
Iterations
Density of d.1.12
N = 50000 Bandwidth = 2.255
Trace of d.1.13
Iterations
Density of d.1.13
N = 50000 Bandwidth = 2.624

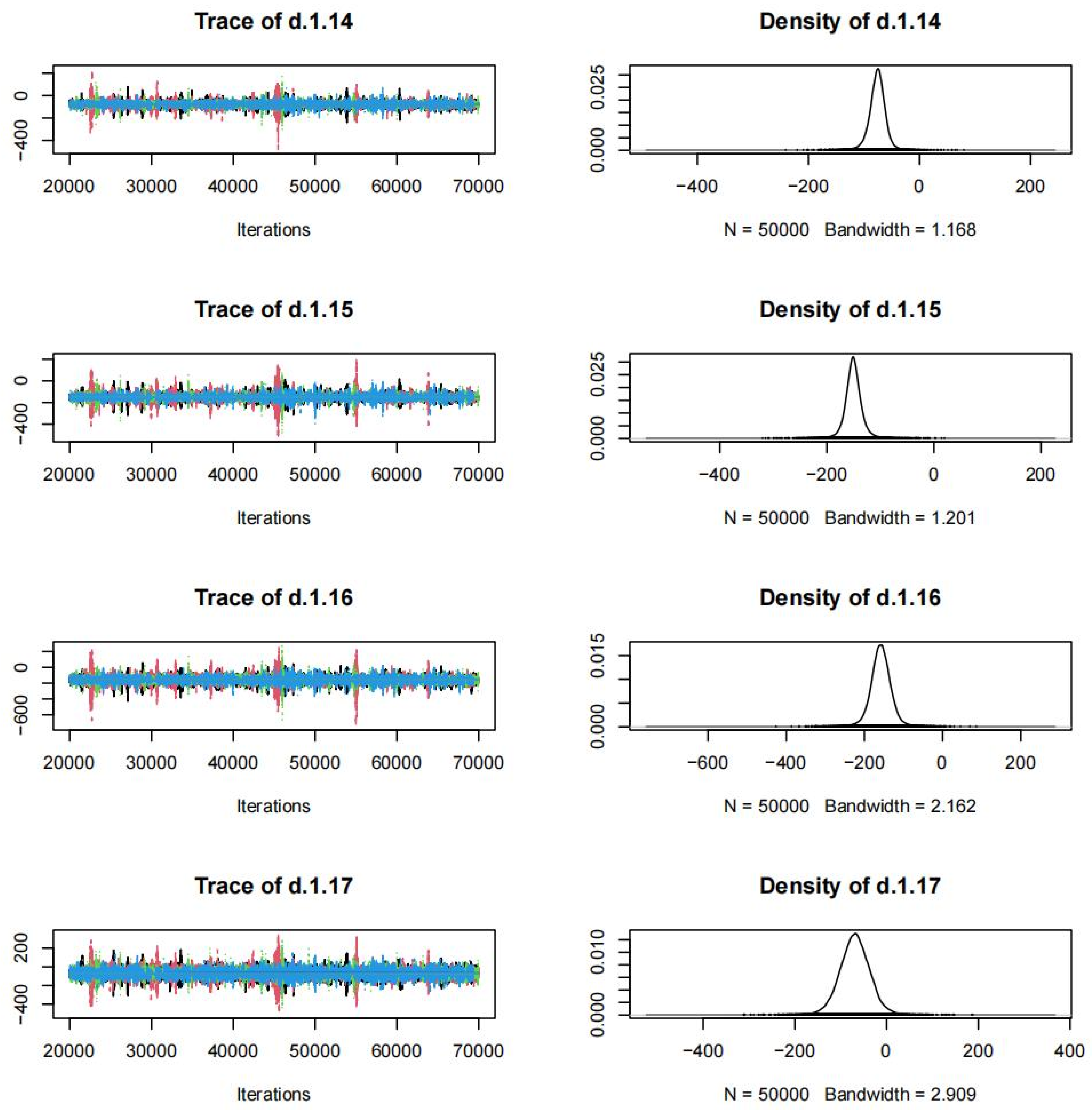
Trace of d.1.14
Iterations
Density of d.1.14
N = 50000 Bandwidth = 1.168
Trace of d.1.15
Iterations
Density of d.1.15
N = 50000 Bandwidth = 1.201
Trace of d.1.16
Iterations
Density of d.1.16
N = 50000 Bandwidth = 2.162
Trace of d.1.17
Iterations
Density of d.1.17
N = 50000 Bandwidth = 2.909

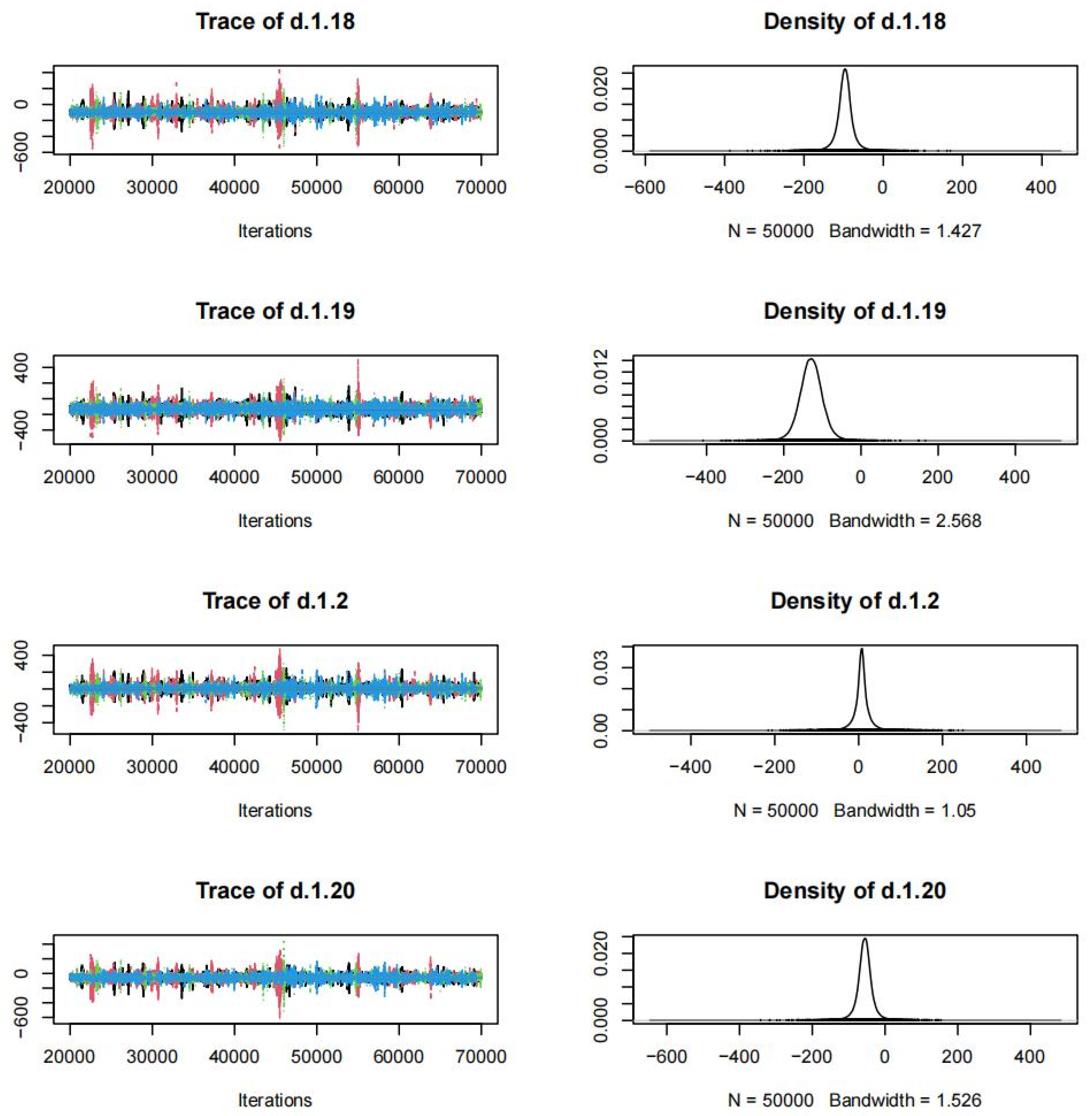
Trace of d.1.18
Iterations
Density of d.1.18
N = 50000 Bandwidth = 1.427
Trace of d.1.19
Iterations
Density of d.1.19
N = 50000 Bandwidth = 2.568
Trace of d.1.2
Iterations
Density of d.1.2
N = 50000 Bandwidth = 1.05
Trace of d.1.20
Iterations
Density of d.1.20
N = 50000 Bandwidth = 1.526

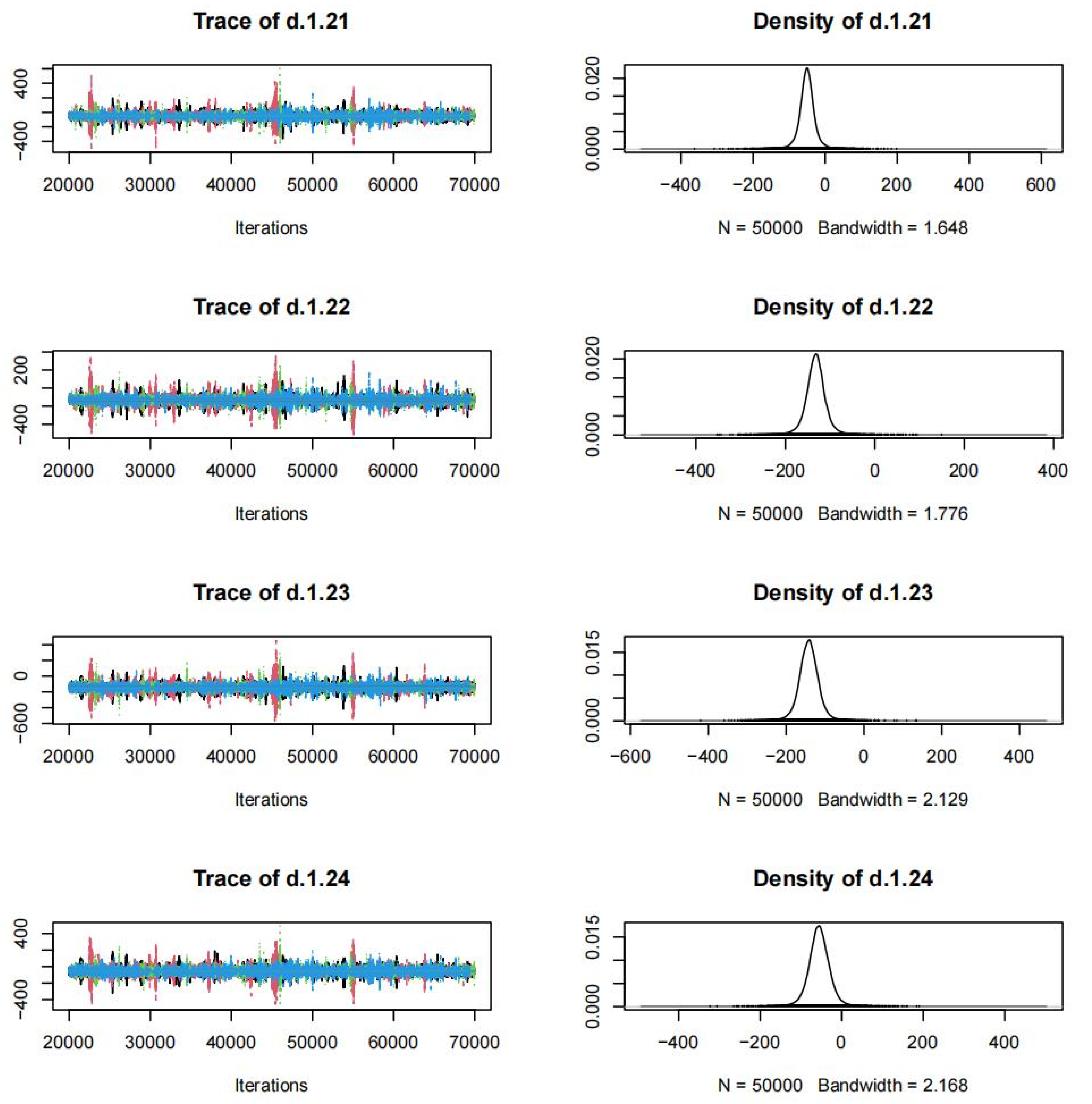
Trace of d.1.21
Iterations
Density of d.1.21
N = 50000 Bandwidth = 1.648
Trace of d.1.22
Iterations
Density of d.1.22
N = 50000 Bandwidth = 1.776
Trace of d.1.23
Iterations
Density of d.1.23
N = 50000 Bandwidth = 2.129
Trace of d.1.24
Iterations
Density of d.1.24
N = 50000 Bandwidth = 2.168

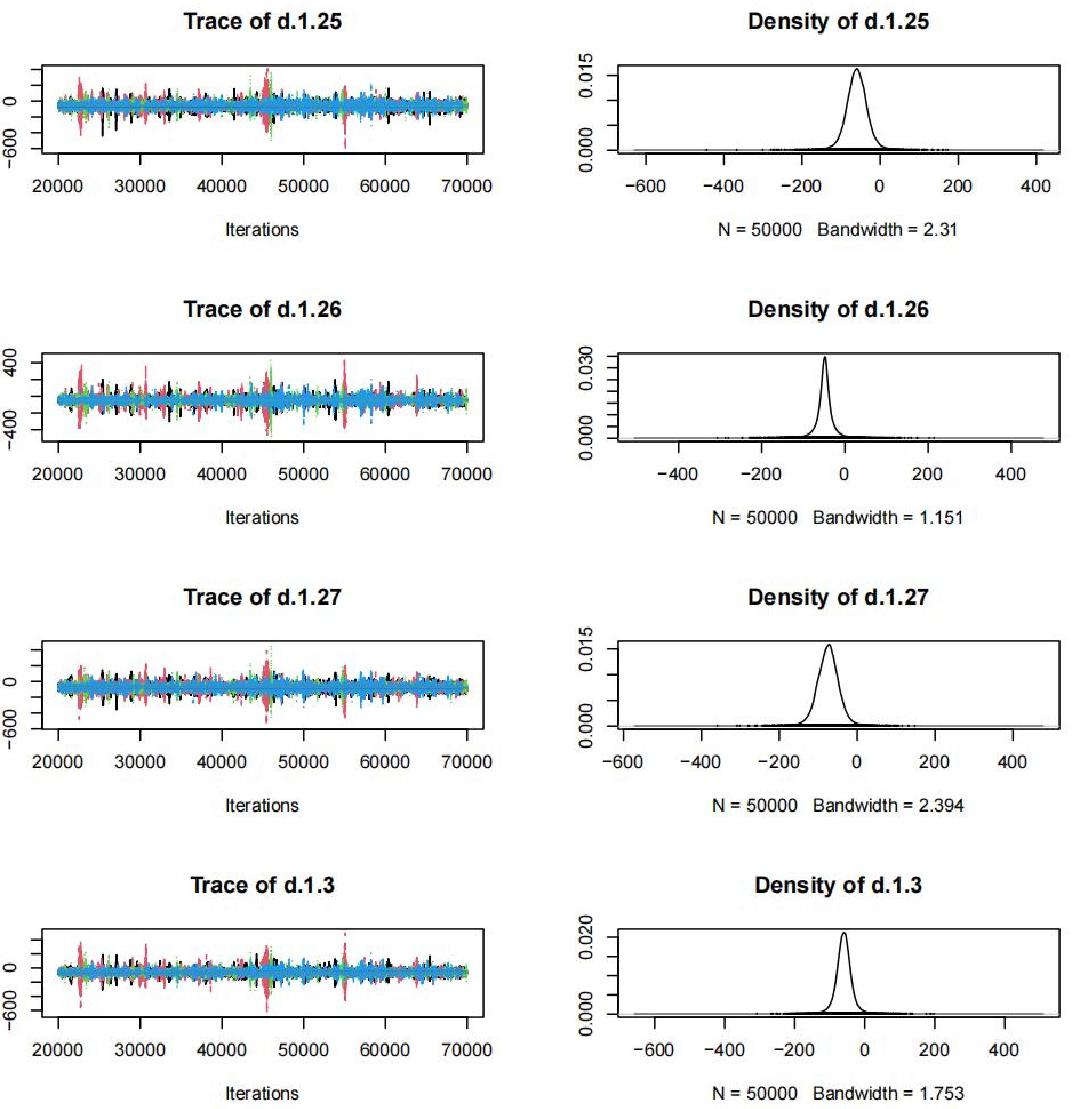
Trace of d.1.25
Iterations
Density of d.1.25
N = 50000 Bandwidth = 2.31
Trace of d.1.26
Iterations
Density of d.1.26
N = 50000 Bandwidth = 1.151
Trace of d.1.27
Iterations
Density of d.1.27
N = 50000 Bandwidth = 2.394
Trace of d.1.3
Iterations
Density of d.1.3
N = 50000 Bandwidth = 1.753

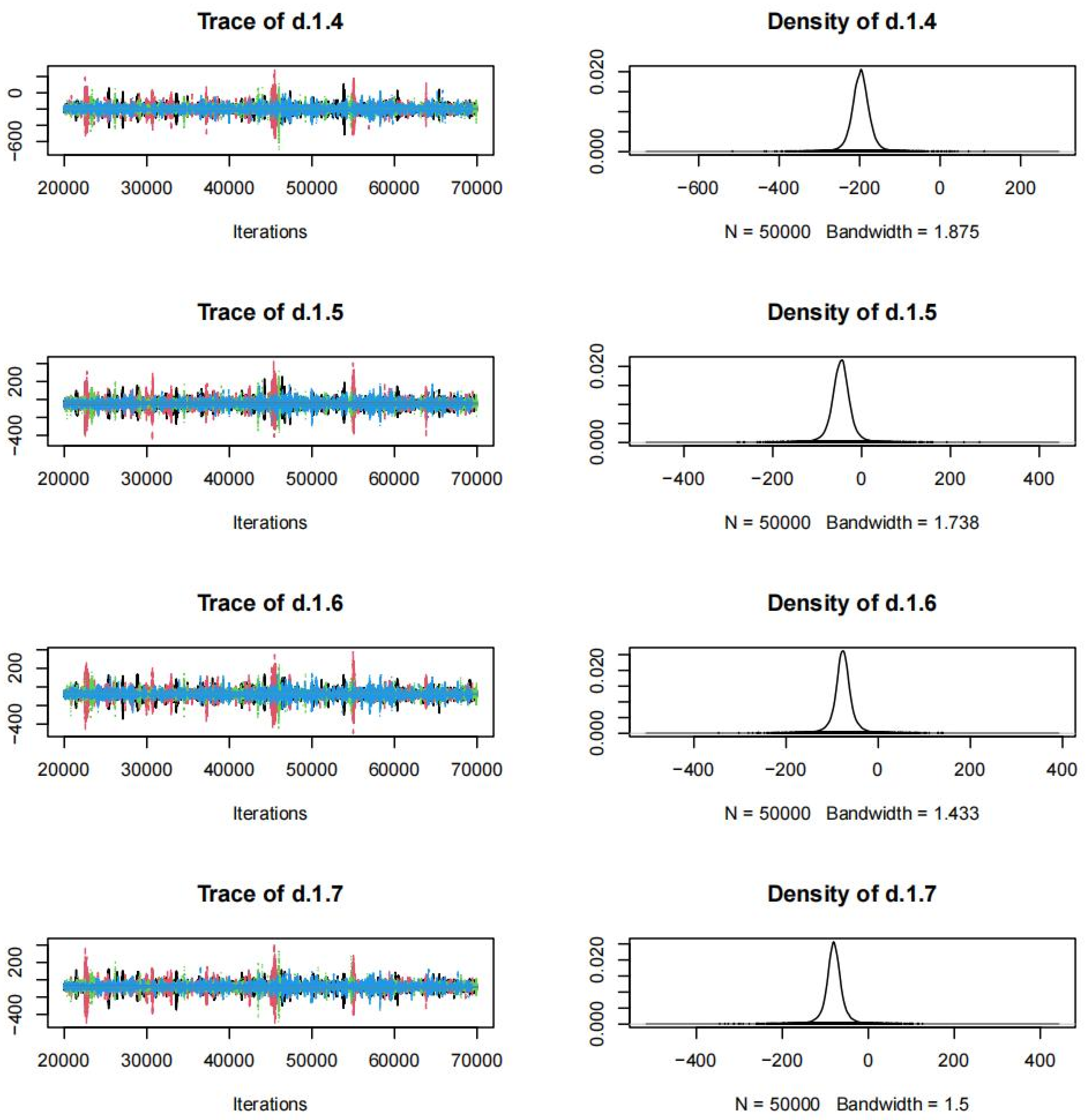
Trace of d.1.4
Iterations
Density of d.1.4
N = 50000 Bandwidth = 1.875
Trace of d.1.5
Iterations
Density of d.1.5
N = 50000 Bandwidth = 1.738
Trace of d.1.6
Iterations
Density of d.1.6
N = 50000 Bandwidth = 1.433
Trace of d.1.7
Iterations
Density of d.1.7
N = 50000 Bandwidth = 1.5

Trace plot and density plot for the ratio of TC

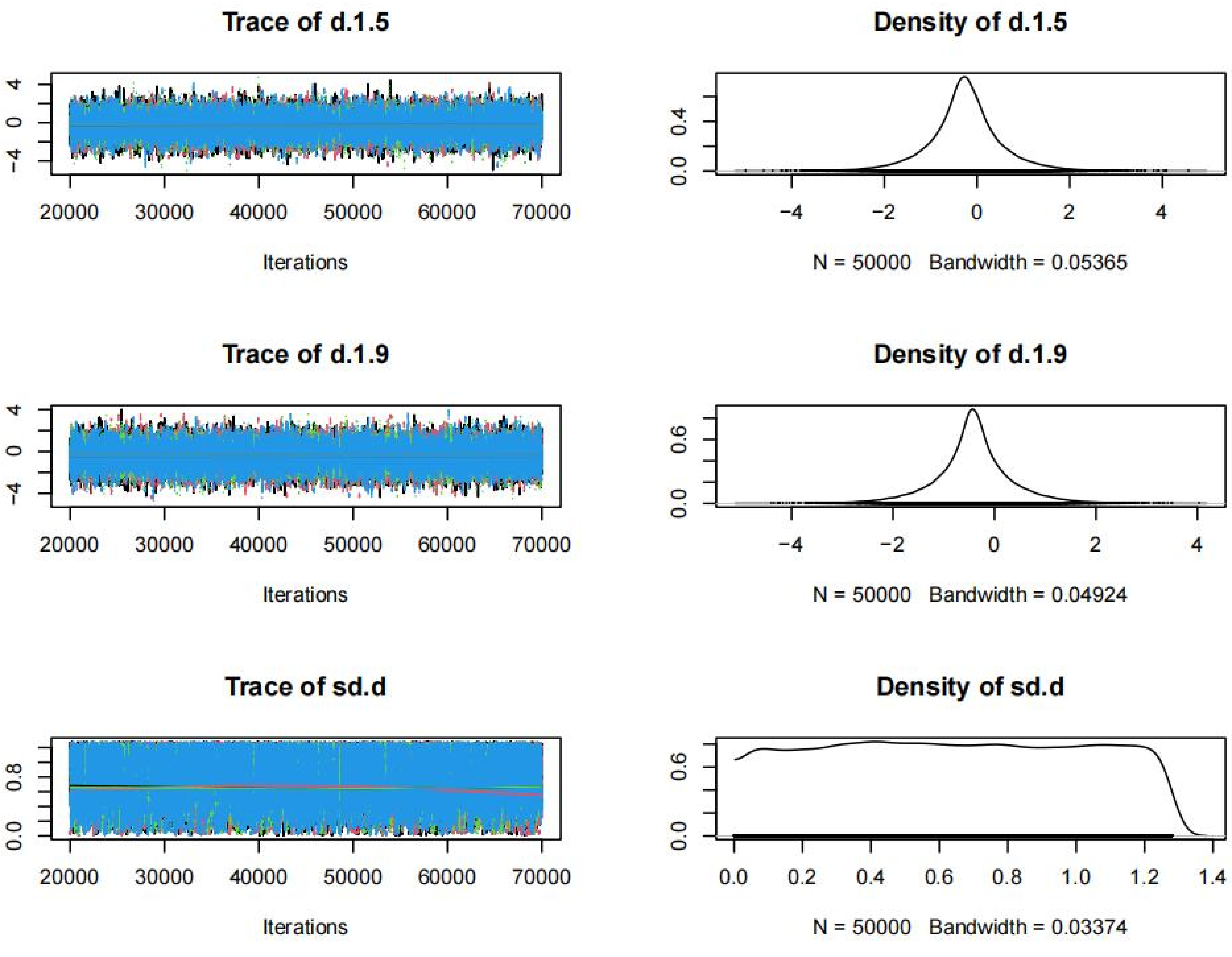

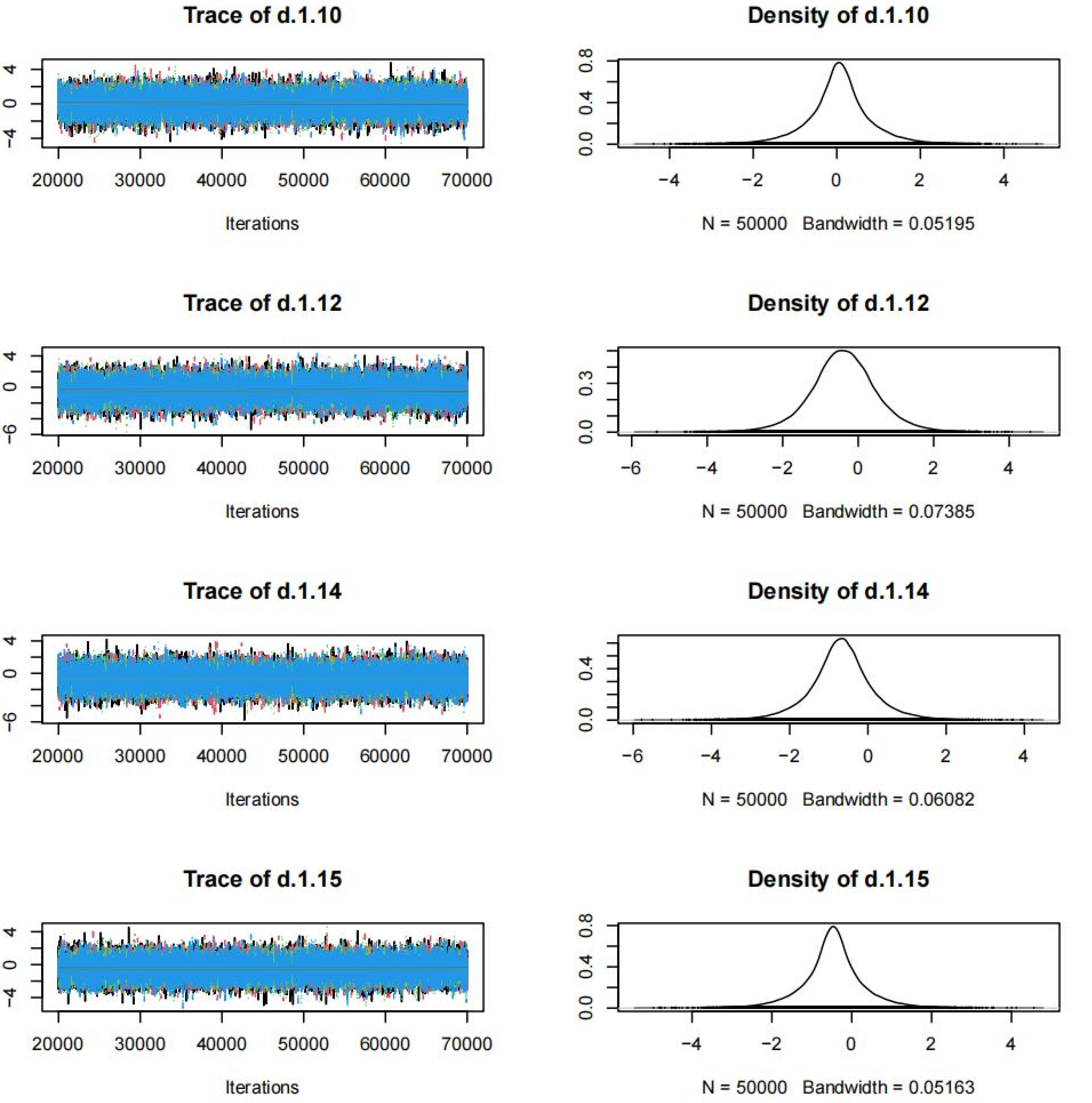

Trace of d.1.10
Iterations
Density of d.1.10
N = 50000 Bandwidth = 0.05195
Trace of d.1.12
Iterations
Density of d.1.12
N = 50000 Bandwidth = 0.07385
Trace of d.1.14
Iterations
Density of d.1.14
N = 50000 Bandwidth = 0.06082
Trace of d.1.15
Iterations
Density of d.1.15
N = 50000 Bandwidth = 0.05163

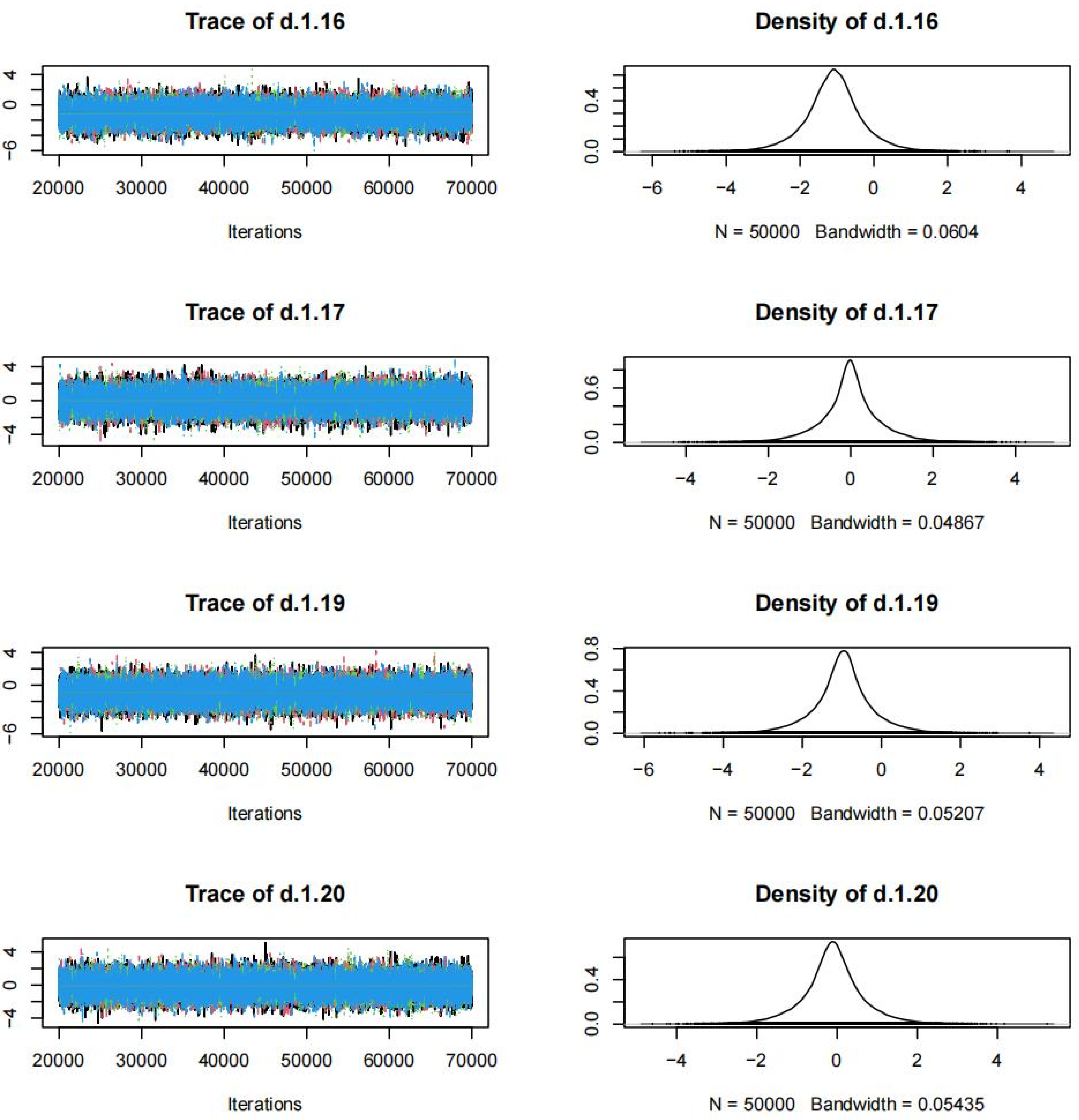

Trace of d.1.16
Iterations
Density of d.1.16
N = 50000 Bandwidth = 0.0604
Trace of d.1.17
Iterations
Density of d.1.17
N = 50000 Bandwidth = 0.04867
Trace of d.1.19
Iterations
Density of d.1.19
N = 50000 Bandwidth = 0.05207
Trace of d.1.20
Iterations
Density of d.1.20
N = 50000 Bandwidth = 0.05435

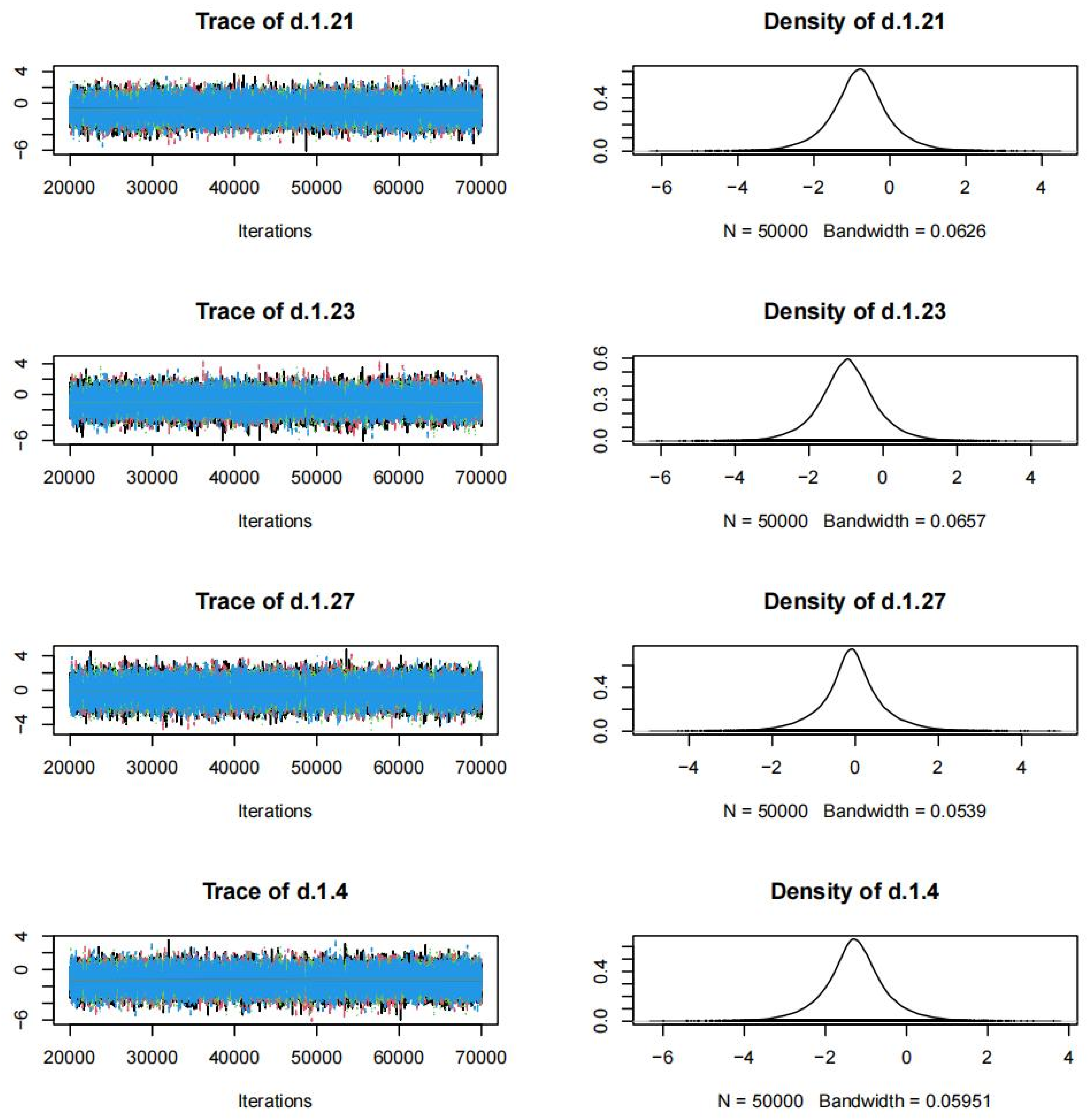
Trace of d.1.21
4
0
-6
20000
30000
40000
50000
60000
70000
Iterations
Density of d.1.21
0.0
0.4
-6
-4
-2
0
2
4
N = 50000 Bandwidth = 0.0626
Trace of d.1.23
4
0
-6
20000
30000
40000
50000
60000
70000
Iterations
Density of d.1.23
0.0
0.3
0.6
-6
-4
-2
0
2
4
N = 50000 Bandwidth = 0.0657
Trace of d.1.27
4
0
-4
20000
30000
40000
50000
60000
70000
Iterations
Density of d.1.27
0.0
0.4
-4
-2
0
2
4
N = 50000 Bandwidth = 0.0539
Trace of d.1.4
4
0
-6
20000
30000
40000
50000
60000
70000
Iterations
Density of d.1.4
0.0
0.4
-6
-4
-2
0
2
4
N = 50000 Bandwidth = 0.05951

Trace plot and density plot for the ratio of TG

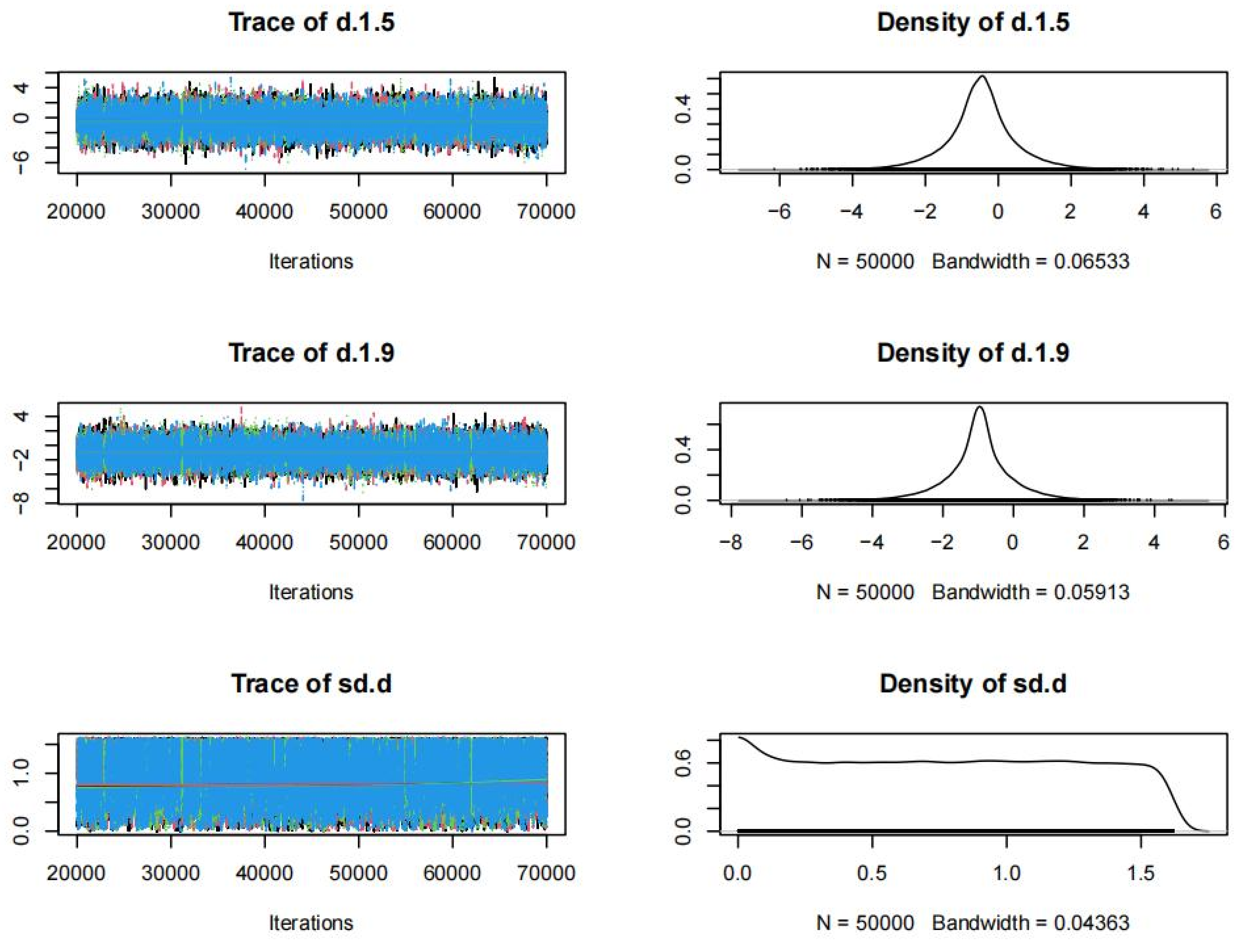

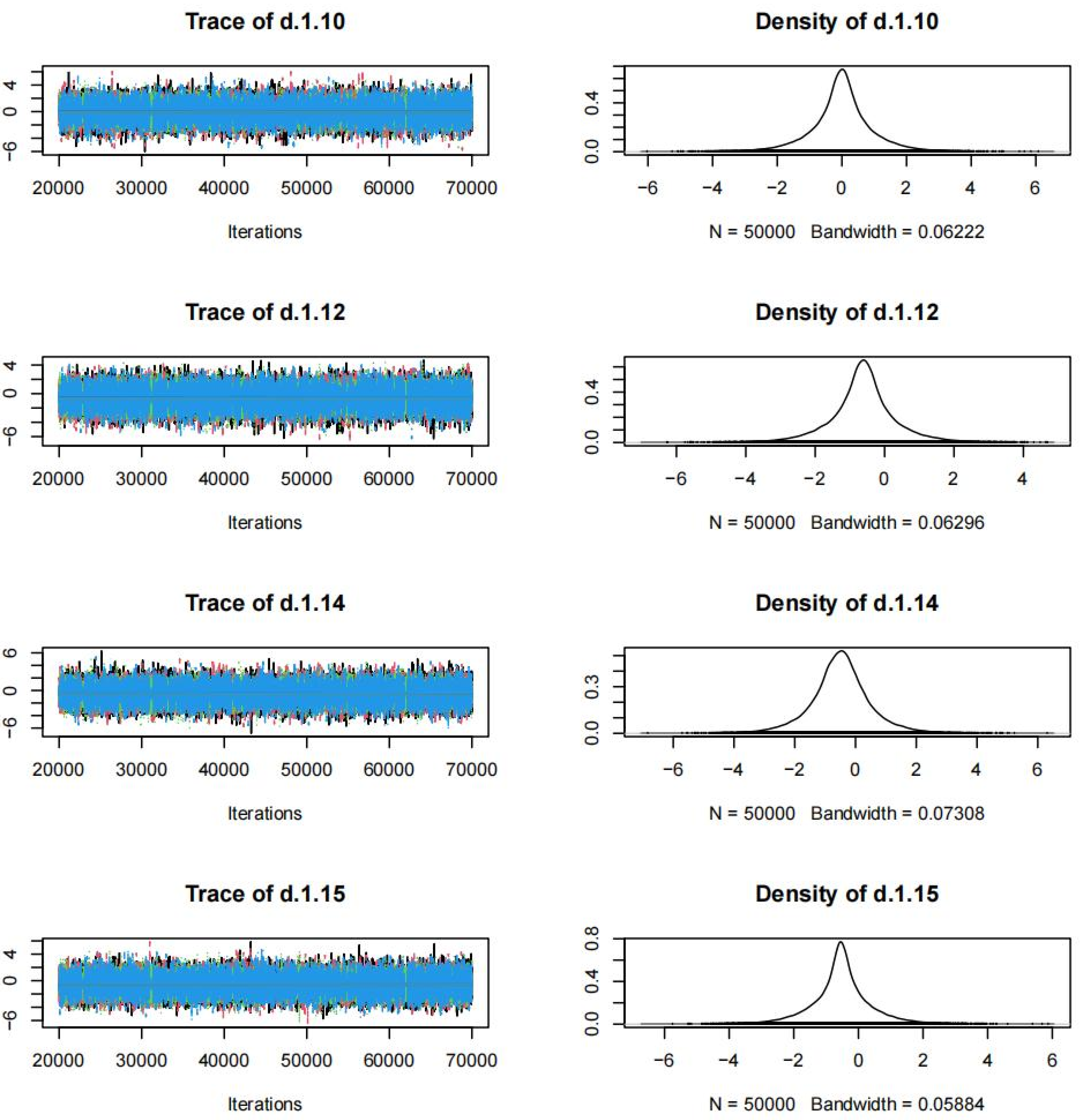
Trace of d.1.10
Iterations
Density of d.1.10
N = 50000 Bandwidth = 0.06222
Trace of d.1.12
Iterations
Density of d.1.12
N = 50000 Bandwidth = 0.06296
Trace of d.1.14
Iterations
Density of d.1.14
N = 50000 Bandwidth = 0.07308
Trace of d.1.15
Iterations
Density of d.1.15
N = 50000 Bandwidth = 0.05884

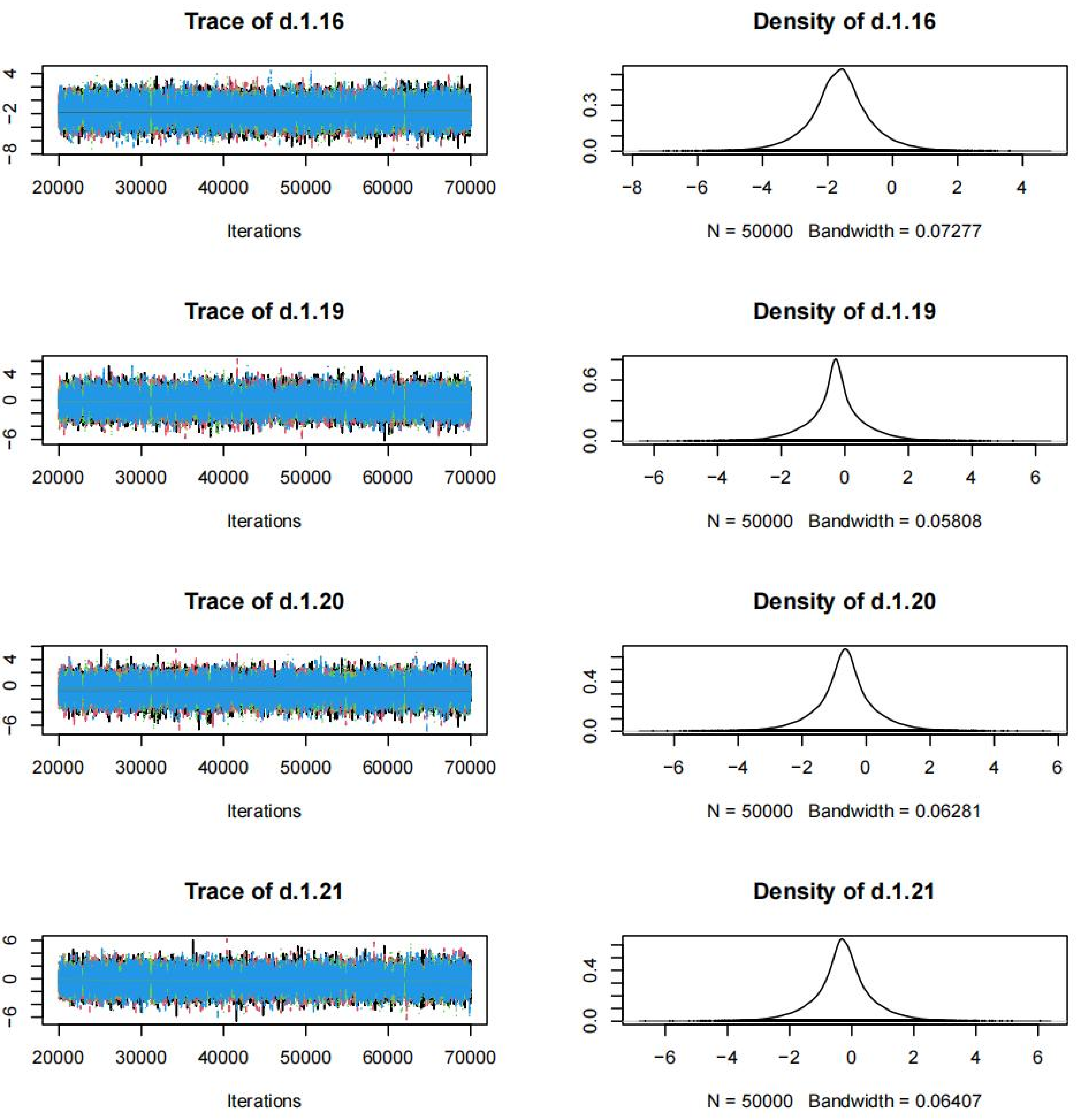
Trace of d.1.16
Iterations
Density of d.1.16
N = 50000 Bandwidth = 0.07277
Trace of d.1.19
Iterations
Density of d.1.19
N = 50000 Bandwidth = 0.05808
Trace of d.1.20
Iterations
Density of d.1.20
N = 50000 Bandwidth = 0.06281
Trace of d.1.21
Iterations
Density of d.1.21
N = 50000 Bandwidth = 0.06407

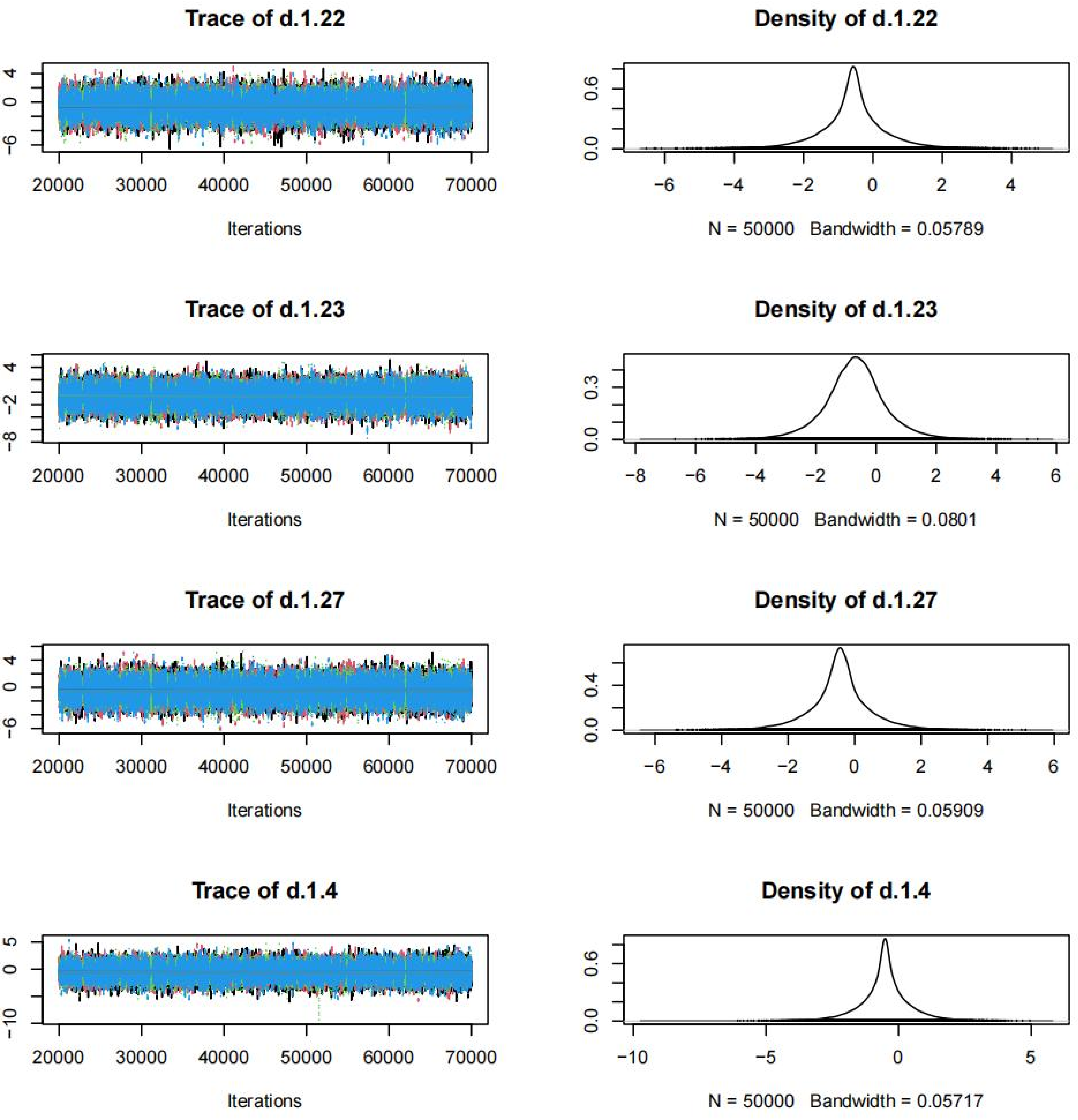
Trace of d.1.22
Iterations
Density of d.1.22
N = 50000 Bandwidth = 0.05789
Trace of d.1.23
Iterations
Density of d.1.23
N = 50000 Bandwidth = 0.0801
Trace of d.1.27
Iterations
Density of d.1.27
N = 50000 Bandwidth = 0.05909
Trace of d.1.4
Iterations
Density of d.1.4
N = 50000 Bandwidth = 0.05717

Trace plot and density plot for the ratio of LDL

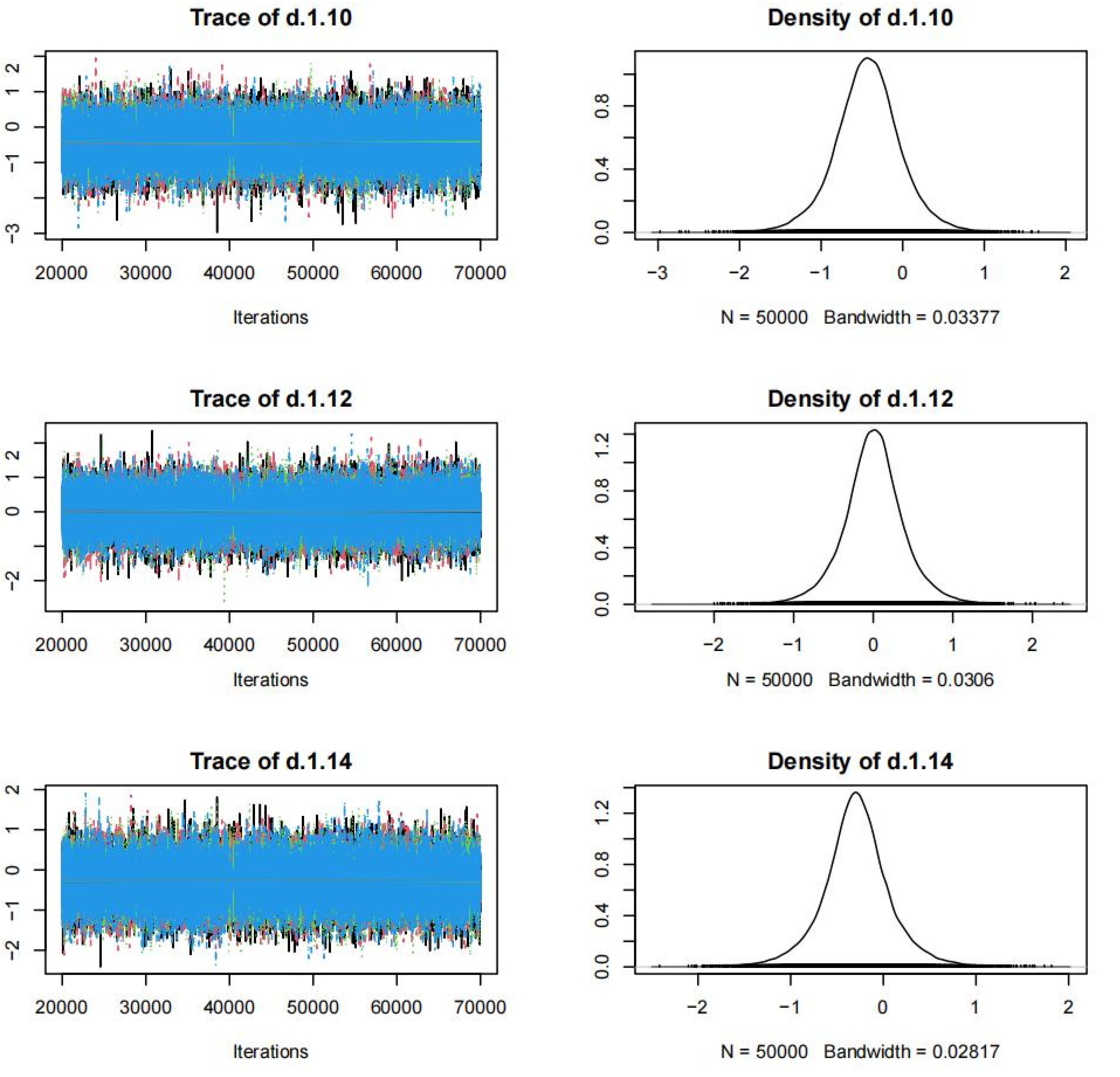

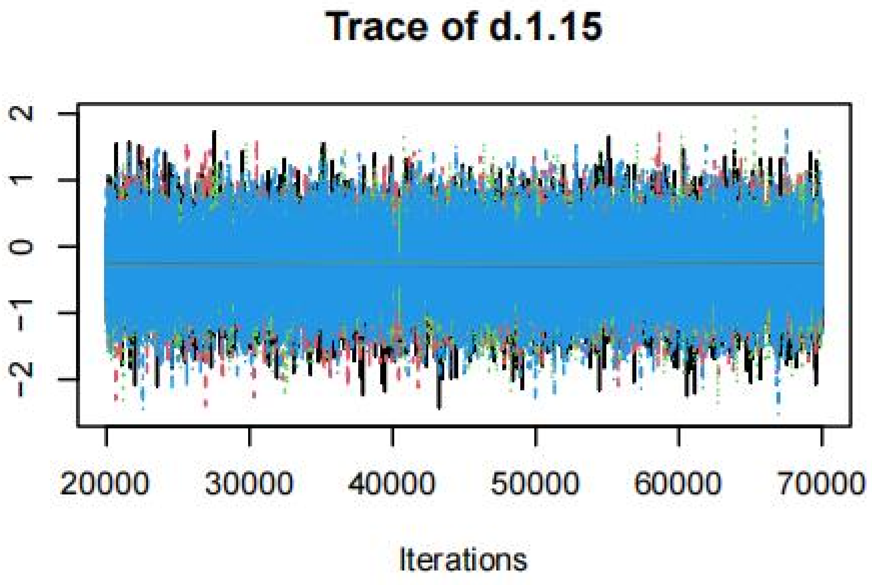
Trace of d.1.15
Iterations
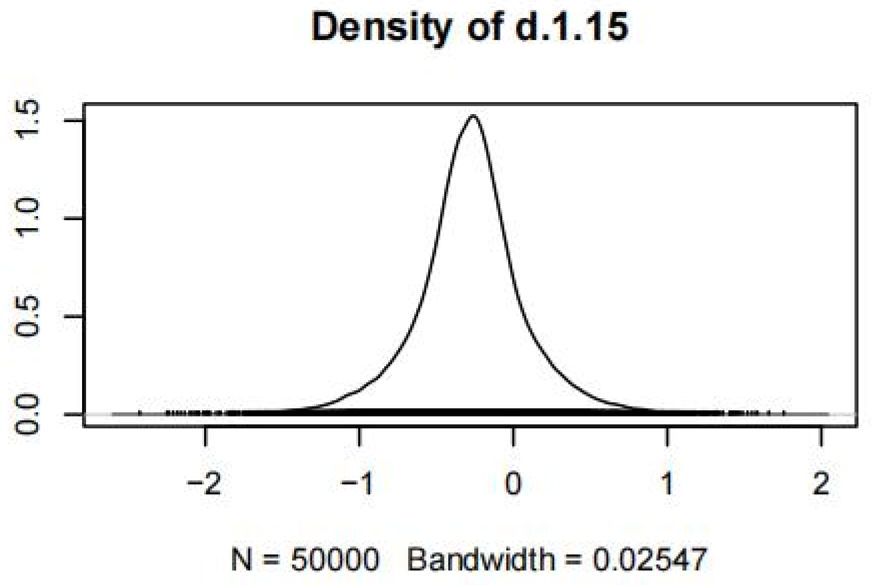
Density of d.1.15
N = 50000 Bandwidth = 0.02547
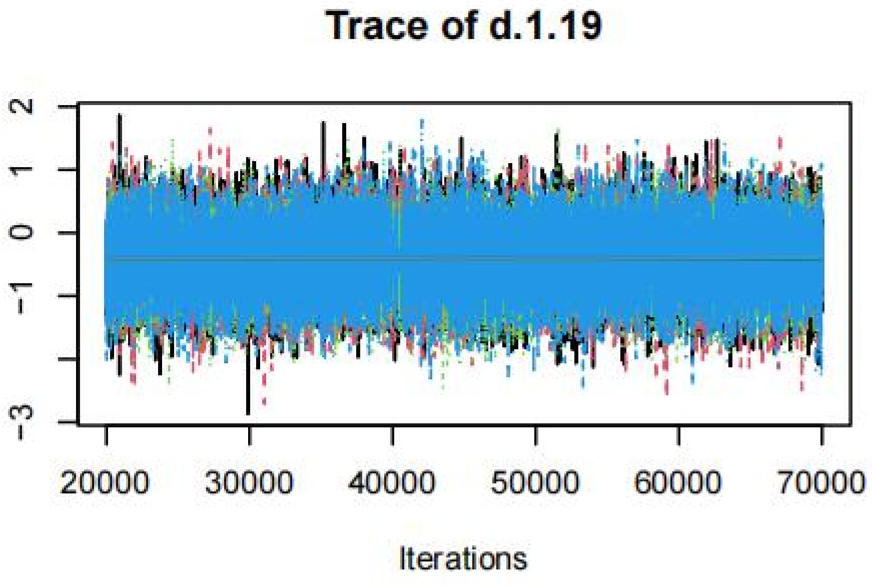
Trace of d.1.19
Iterations
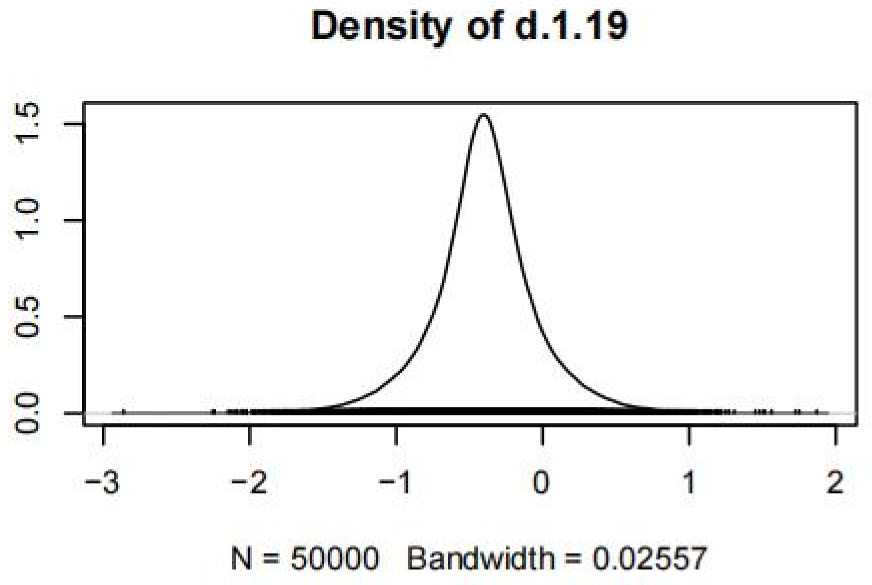
Density of d.1.19
N = 50000 Bandwidth = 0.02557
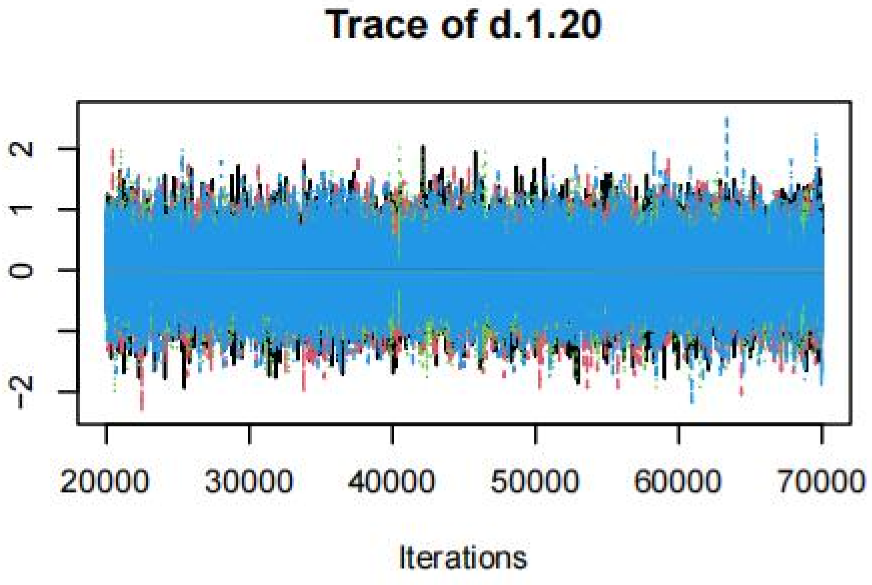
Trace of d.1.20
Iterations
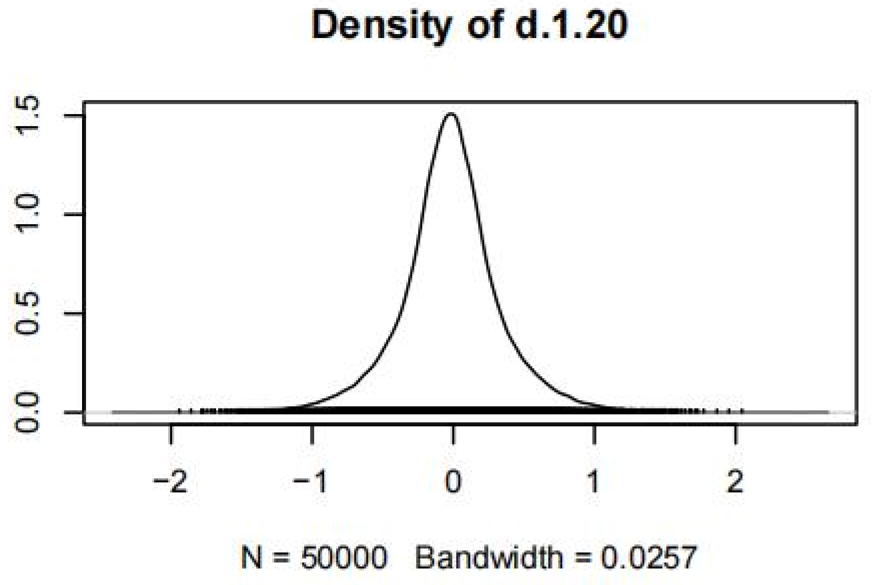
Density of d.1.20
N = 50000 Bandwidth = 0.0257

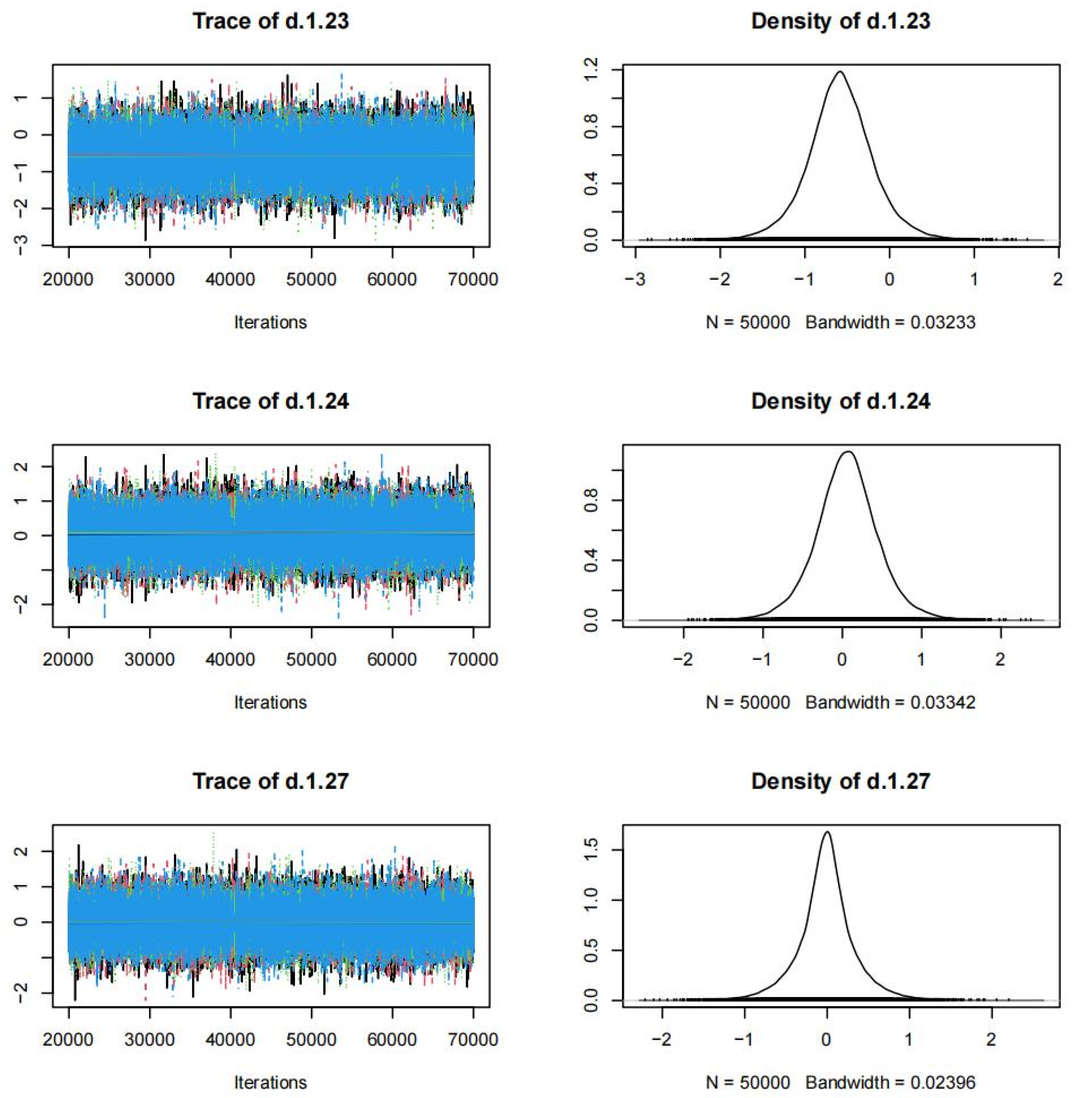
Trace of d.1.23
Iterations
Density of d.1.23
N = 50000 Bandwidth = 0.03233
Trace of d.1.24
Iterations
Density of d.1.24
N = 50000 Bandwidth = 0.03342
Trace of d.1.27
Iterations
Density of d.1.27
N = 50000 Bandwidth = 0.02396

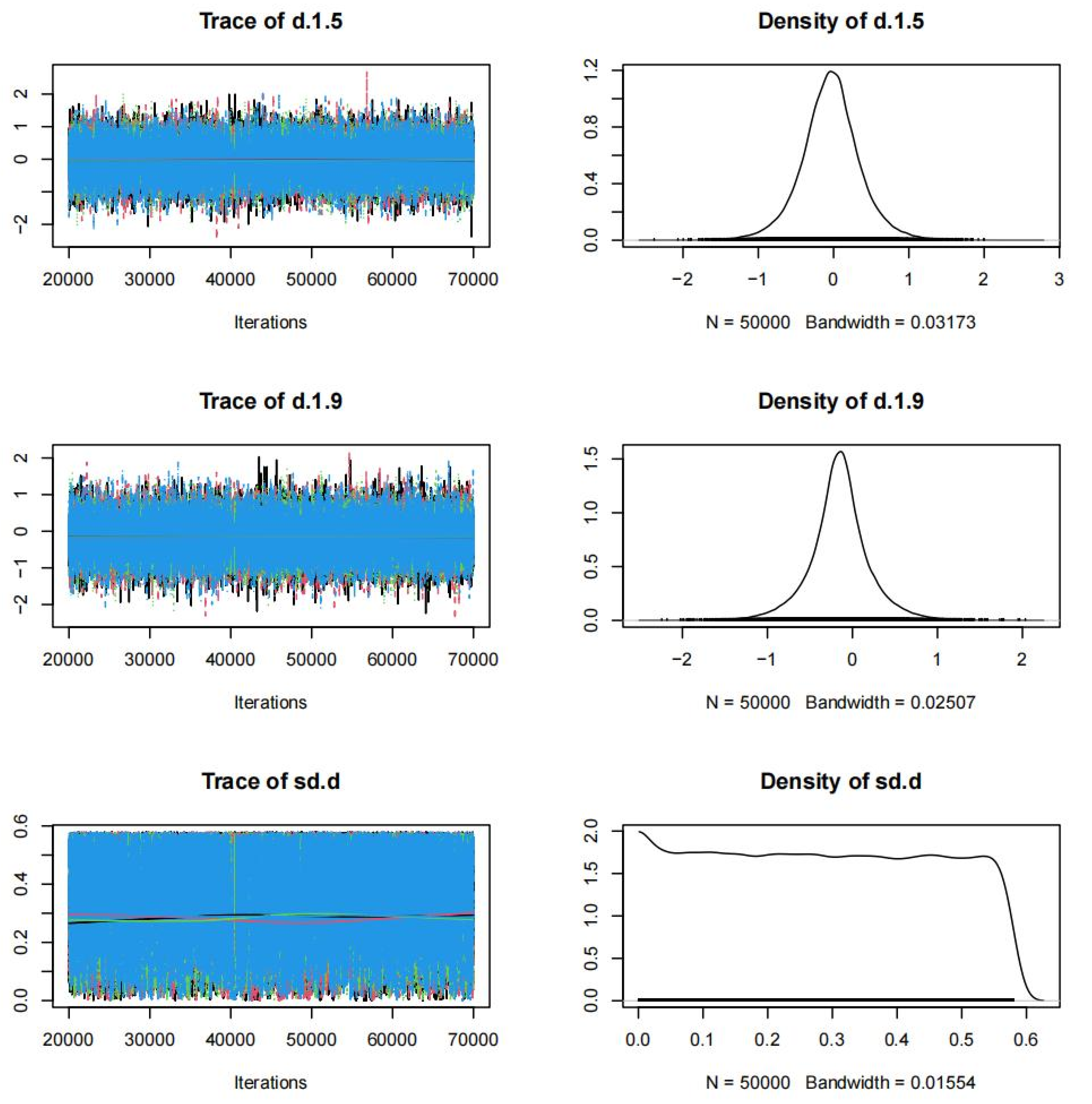
Trace of d.1.5
Iterations
20000 30000 40000 50000 60000 70000
-2 0 1 2
Density of d.1.5
N = 50000 Bandwidth = 0.03173
-2 -1 0 1 2 3
0.0 0.4 0.8 1.2
Trace of d.1.9
Iterations
20000 30000 40000 50000 60000 70000
-2 -1 0 1 2
Density of d.1.9
N = 50000 Bandwidth = 0.02507
-2 -1 0 1 2
0.0 0.5 1.0 1.5
Trace of sd.d
Iterations
20000 30000 40000 50000 60000 70000
0.0 0.2 0.4 0.6
Density of sd.d
N = 50000 Bandwidth = 0.01554
0.0 0.1 0.2 0.3 0.4 0.5 0.6
0.0 0.5 1.0 1.5 2.0

Trace plot and density plot for the ratio of HDL

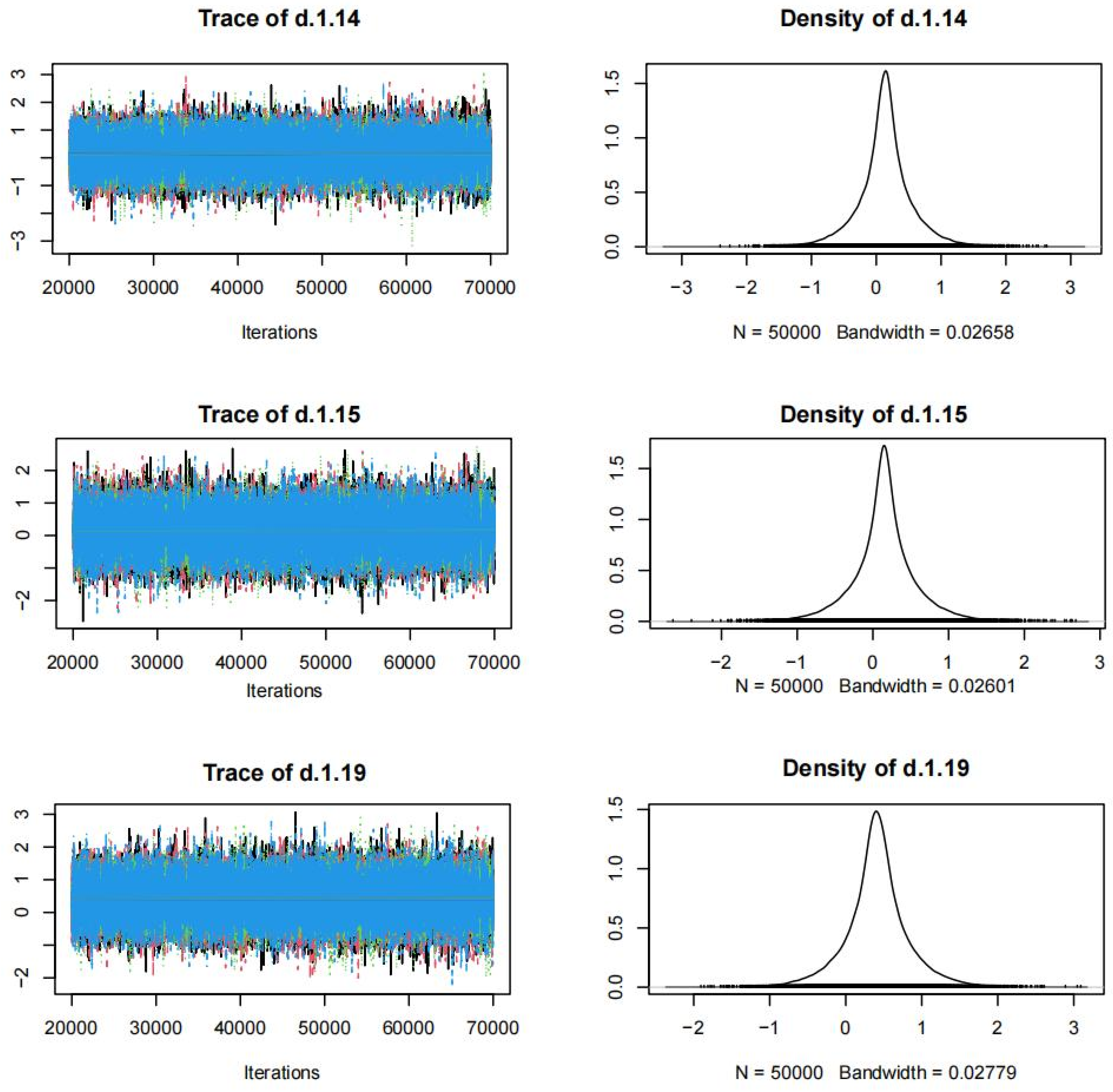

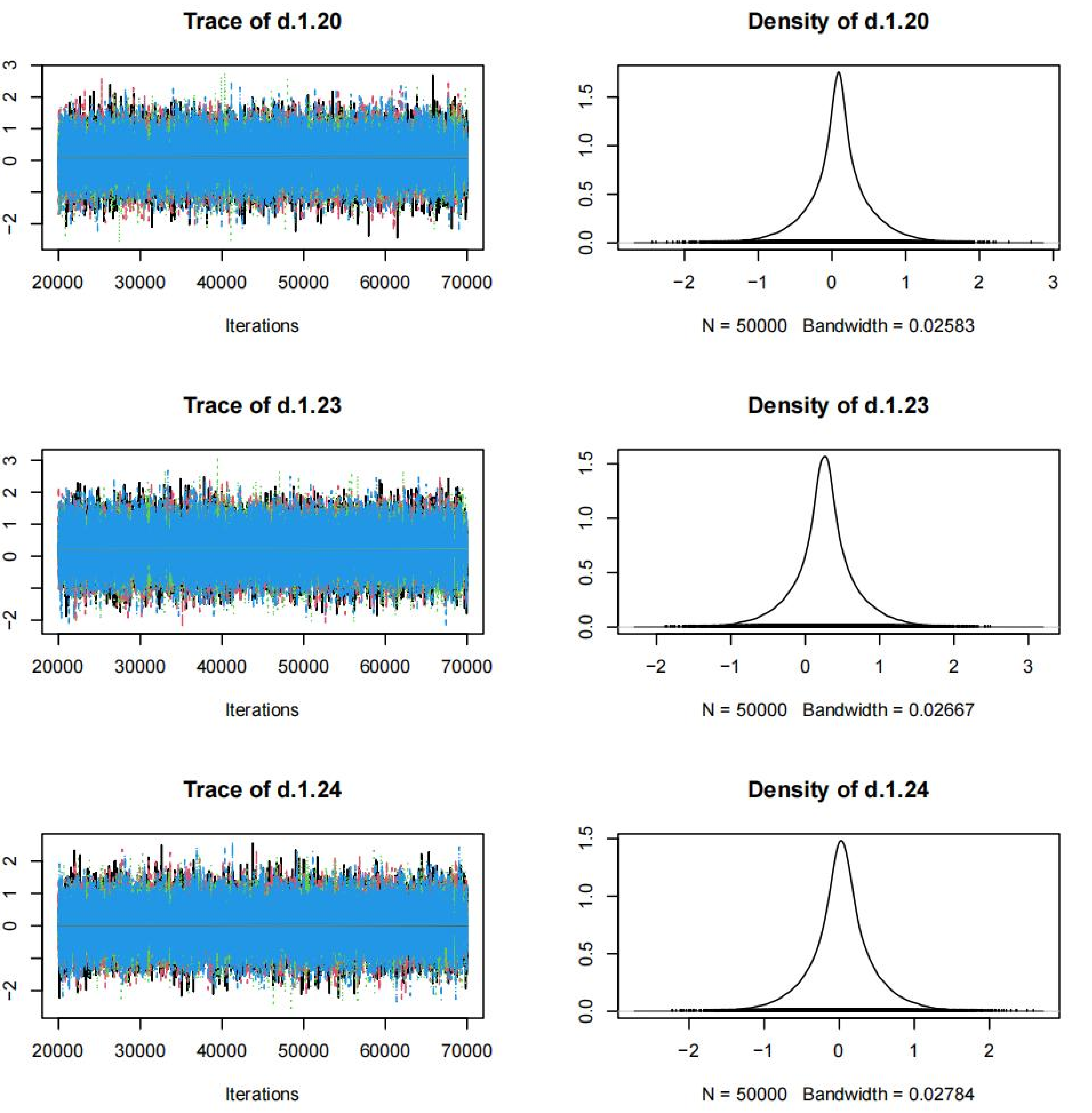
Trace of d.1.20
Iterations
Density of d.1.20
N = 50000 Bandwidth = 0.02583
Trace of d.1.23
Iterations
Density of d.1.23
N = 50000 Bandwidth = 0.02667
Trace of d.1.24
Iterations
Density of d.1.24
N = 50000 Bandwidth = 0.02784

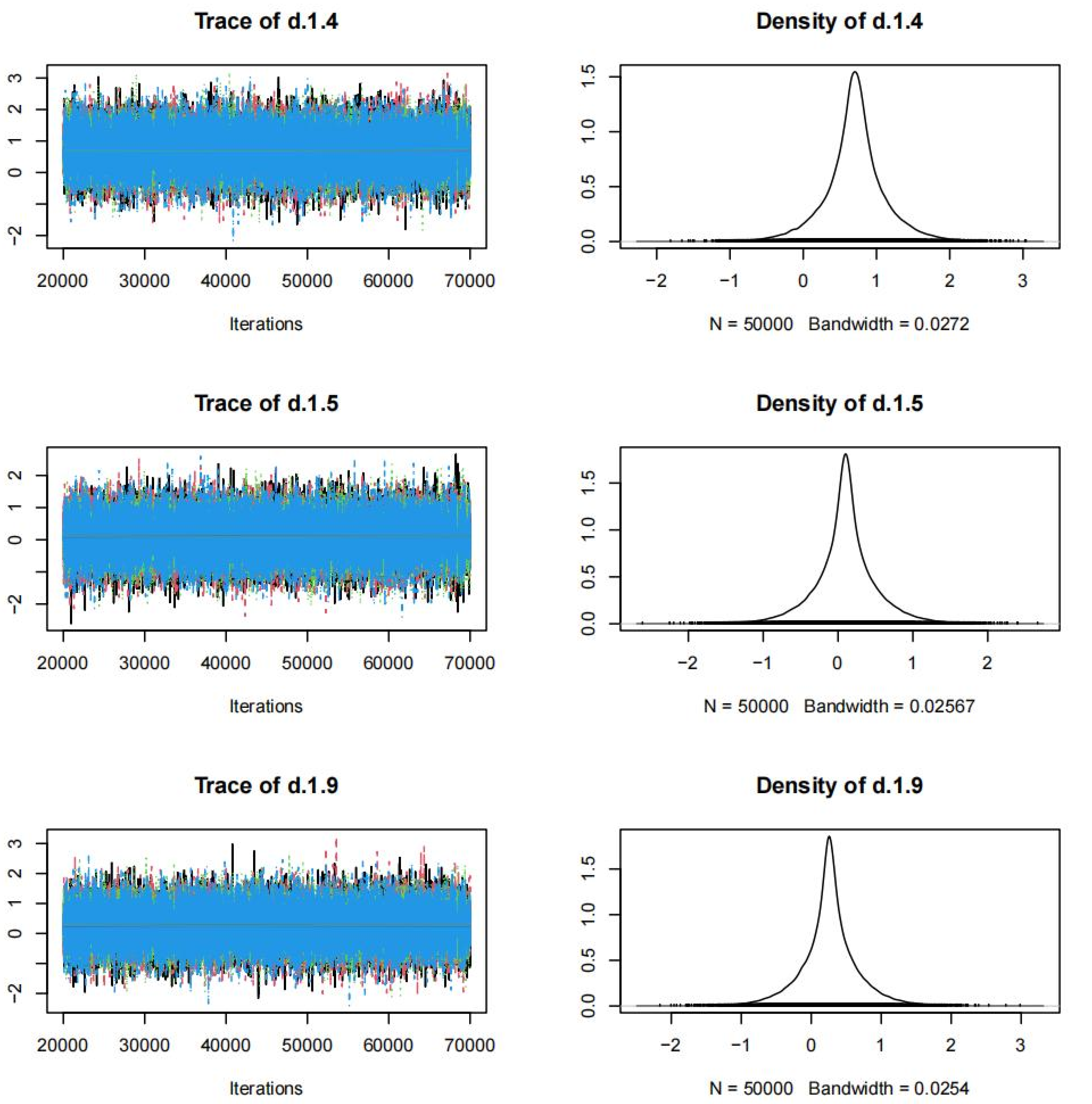

Trace of d.1.4
Iterations
Density of d.1.4
N = 50000 Bandwidth = 0.0272
Trace of d.1.5
Iterations
Density of d.1.5
N = 50000 Bandwidth = 0.02567
Trace of d.1.9
Iterations
Density of d.1.9
N = 50000 Bandwidth = 0.0254

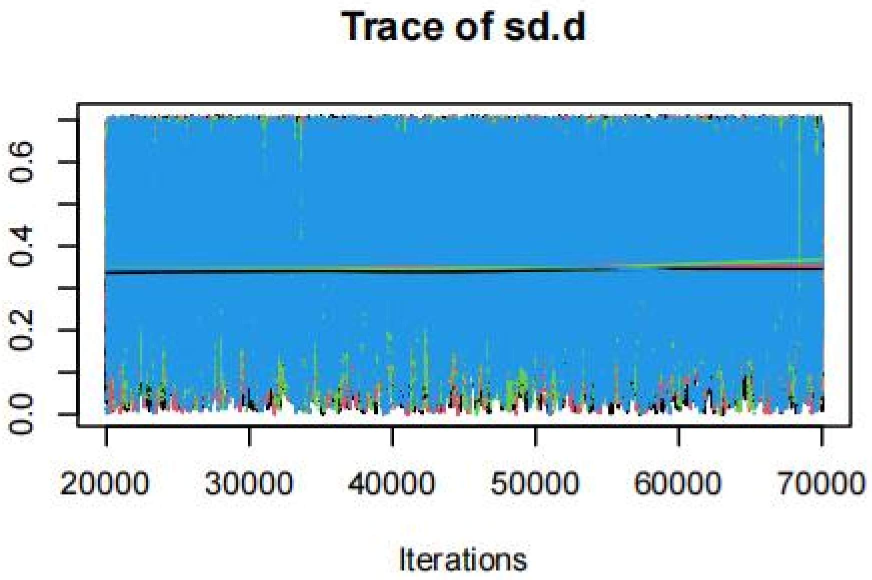
Trace of sd.d
0.0
0.2
0.4
0.6
20000
30000
40000
50000
60000
70000
Iterations

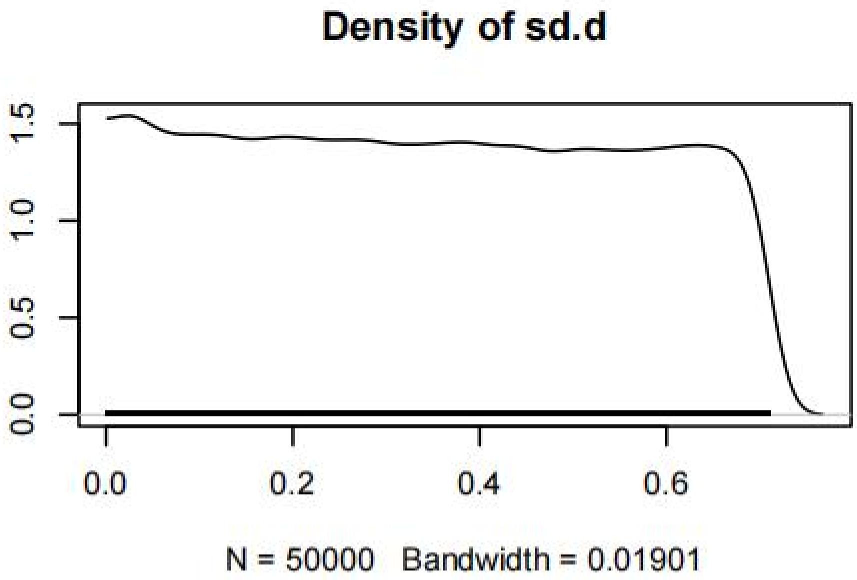
Density of sd.d
0.0
0.5
1.0
1.5
0.0
0.2
0.4
0.6
N = 50000 Bandwidth = 0.01901

Trace plot and density plot for the ratio of TCMSS

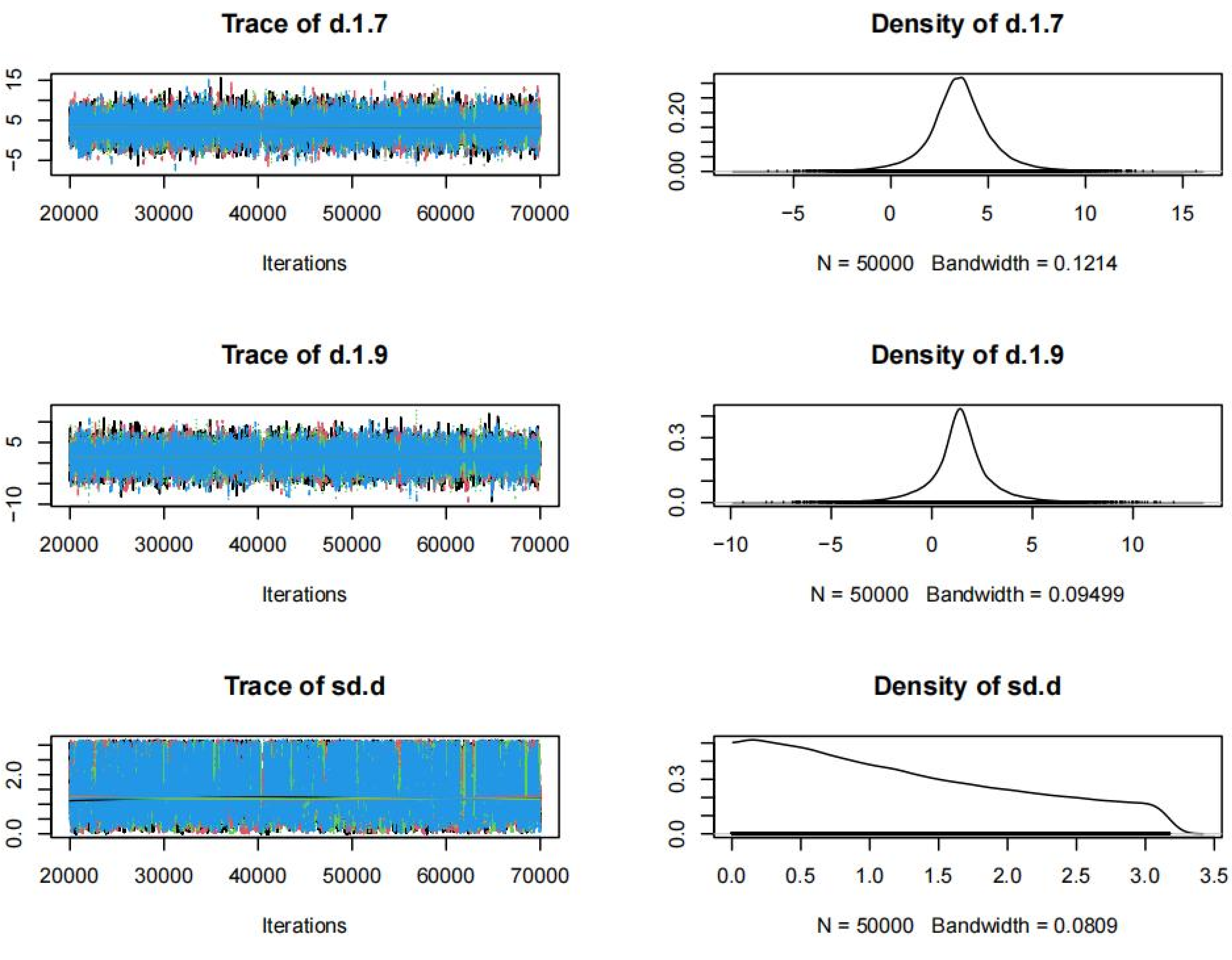

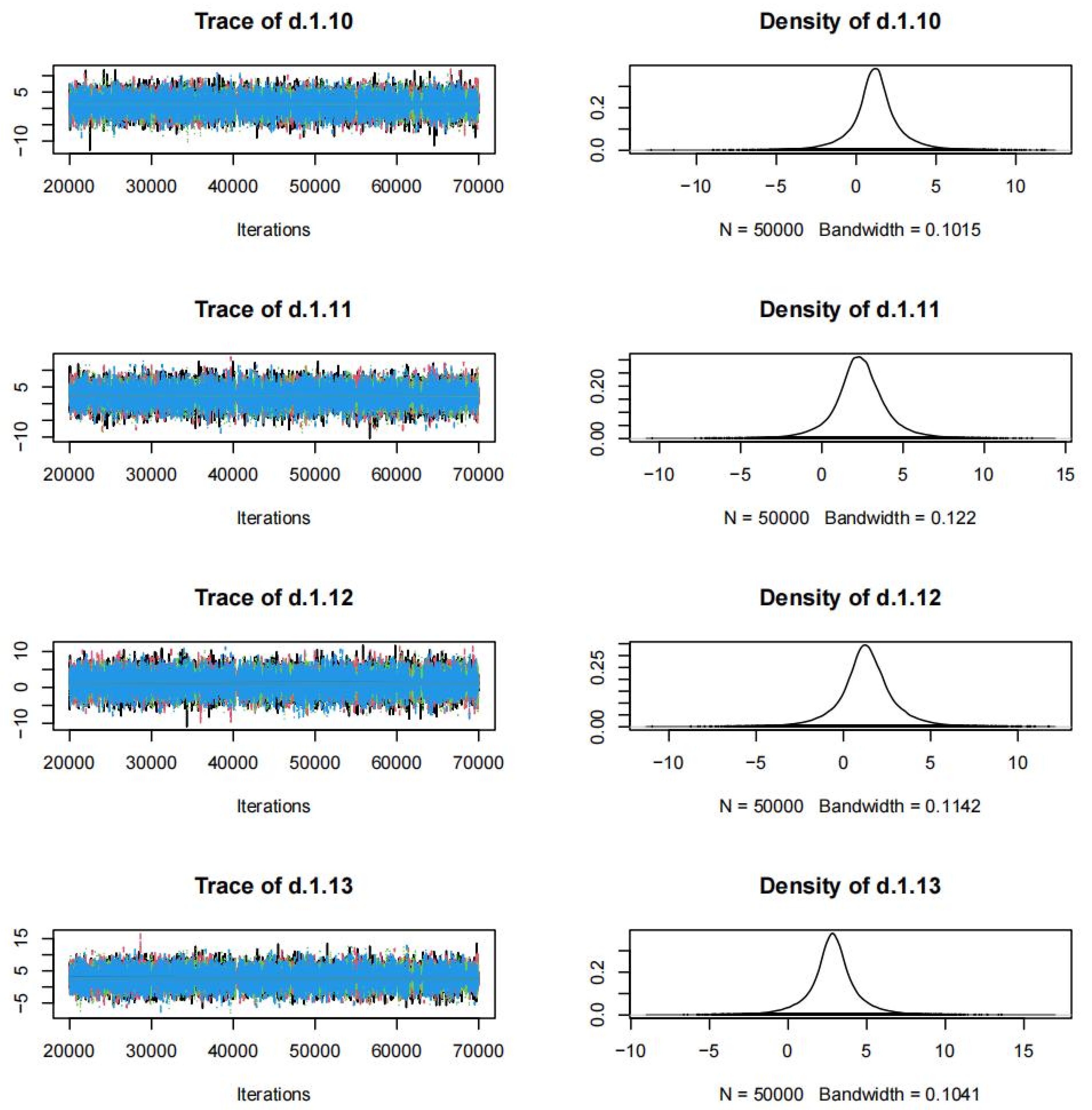

Trace of d.1.10
Iterations
Density of d.1.10
N = 50000 Bandwidth = 0.1015
Trace of d.1.11
Iterations
Density of d.1.11
N = 50000 Bandwidth = 0.122
Trace of d.1.12
Iterations
Density of d.1.12
N = 50000 Bandwidth = 0.1142
Trace of d.1.13
Iterations
Density of d.1.13
N = 50000 Bandwidth = 0.1041

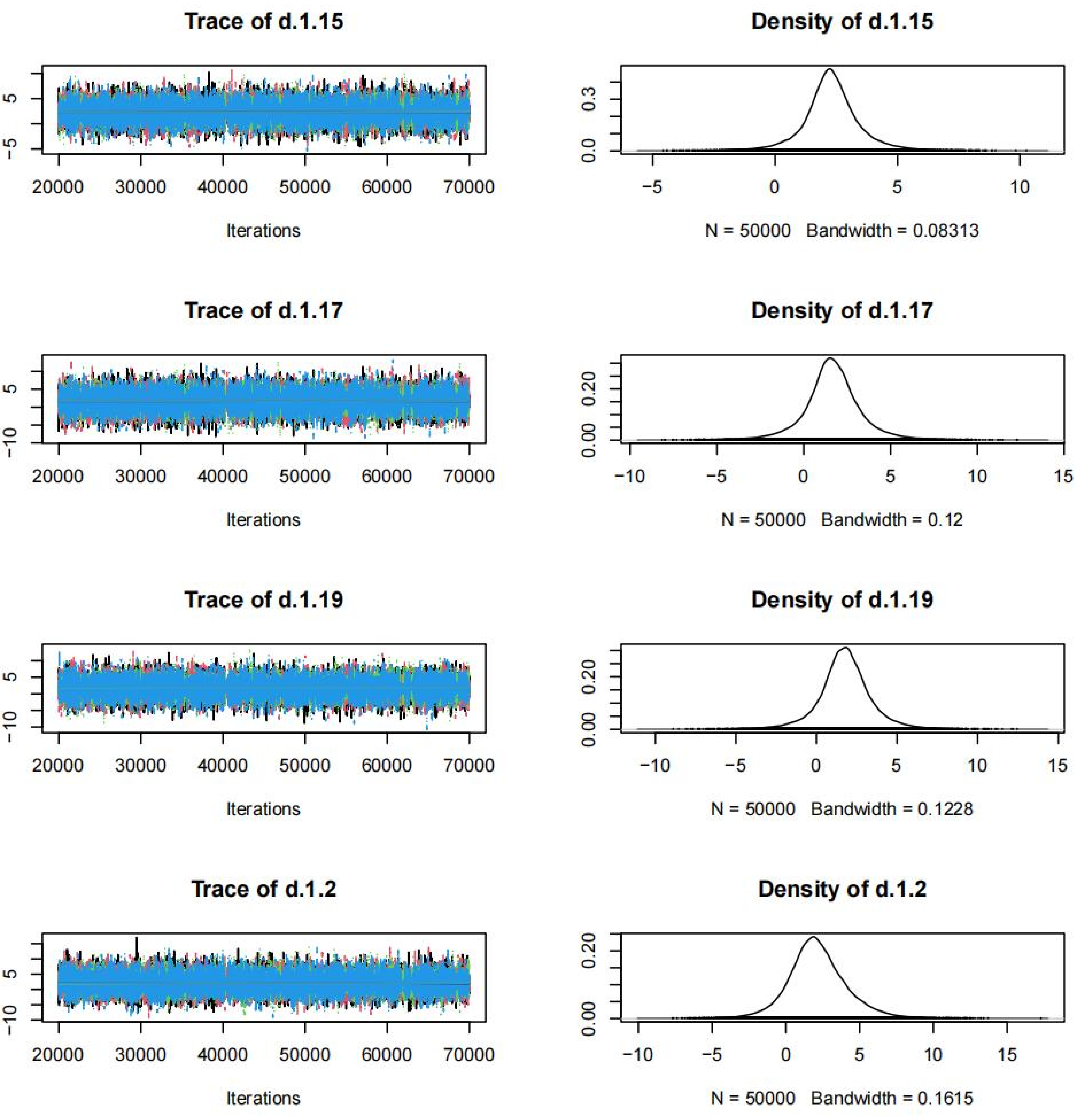
Trace of d.1.15
Iterations
Density of d.1.15
N = 50000 Bandwidth = 0.08313
Trace of d.1.17
Iterations
Density of d.1.17
N = 50000 Bandwidth = 0.12
Trace of d.1.19
Iterations
Density of d.1.19
N = 50000 Bandwidth = 0.1228
Trace of d.1.2
Iterations
Density of d.1.2
N = 50000 Bandwidth = 0.1615

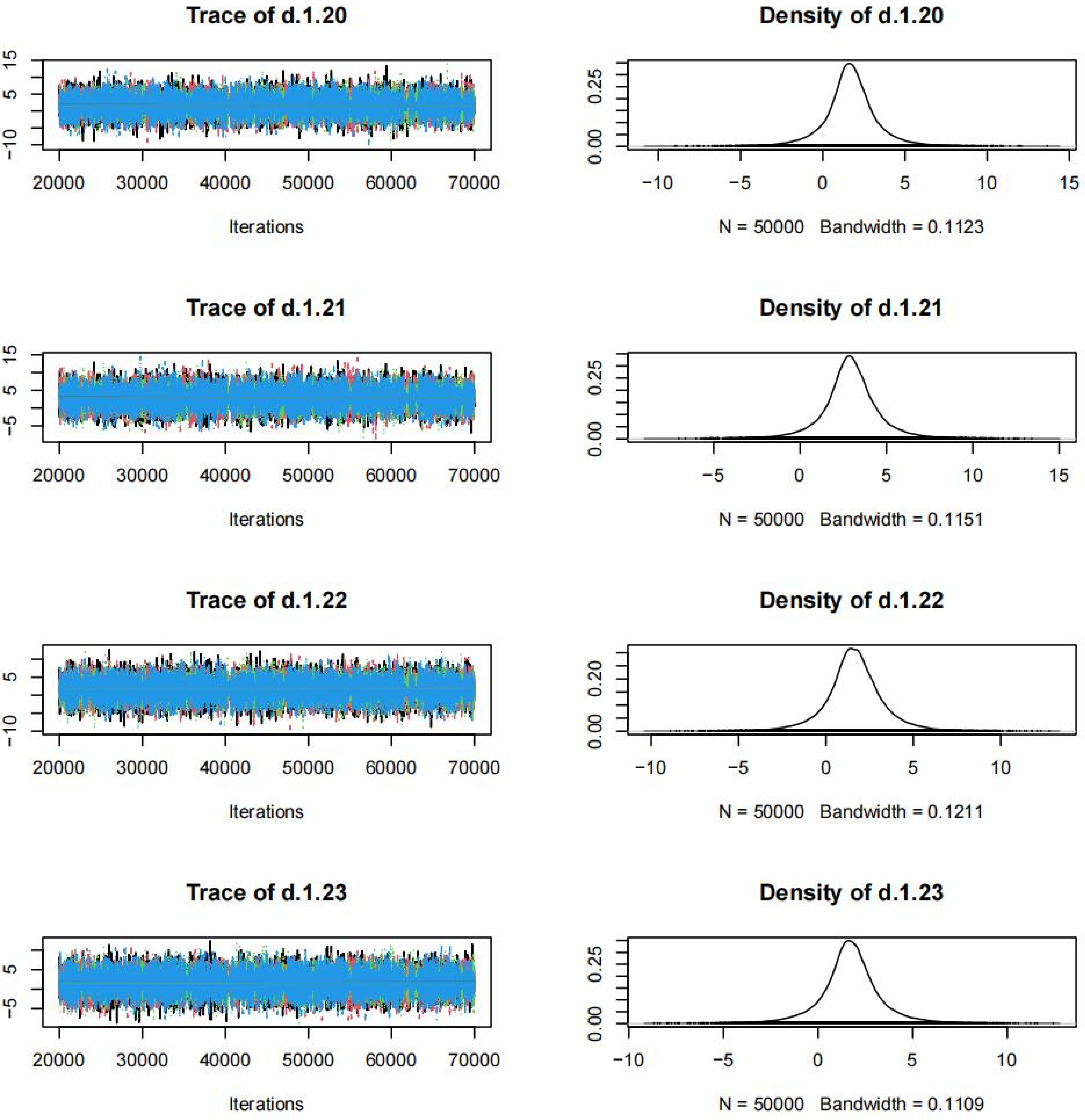
Trace of d.1.20
Iterations
Density of d.1.20
N = 50000 Bandwidth = 0.1123
Trace of d.1.21
Iterations
Density of d.1.21
N = 50000 Bandwidth = 0.1151
Trace of d.1.22
Iterations
Density of d.1.22
N = 50000 Bandwidth = 0.1211
Trace of d.1.23
Iterations
Density of d.1.23
N = 50000 Bandwidth = 0.1109

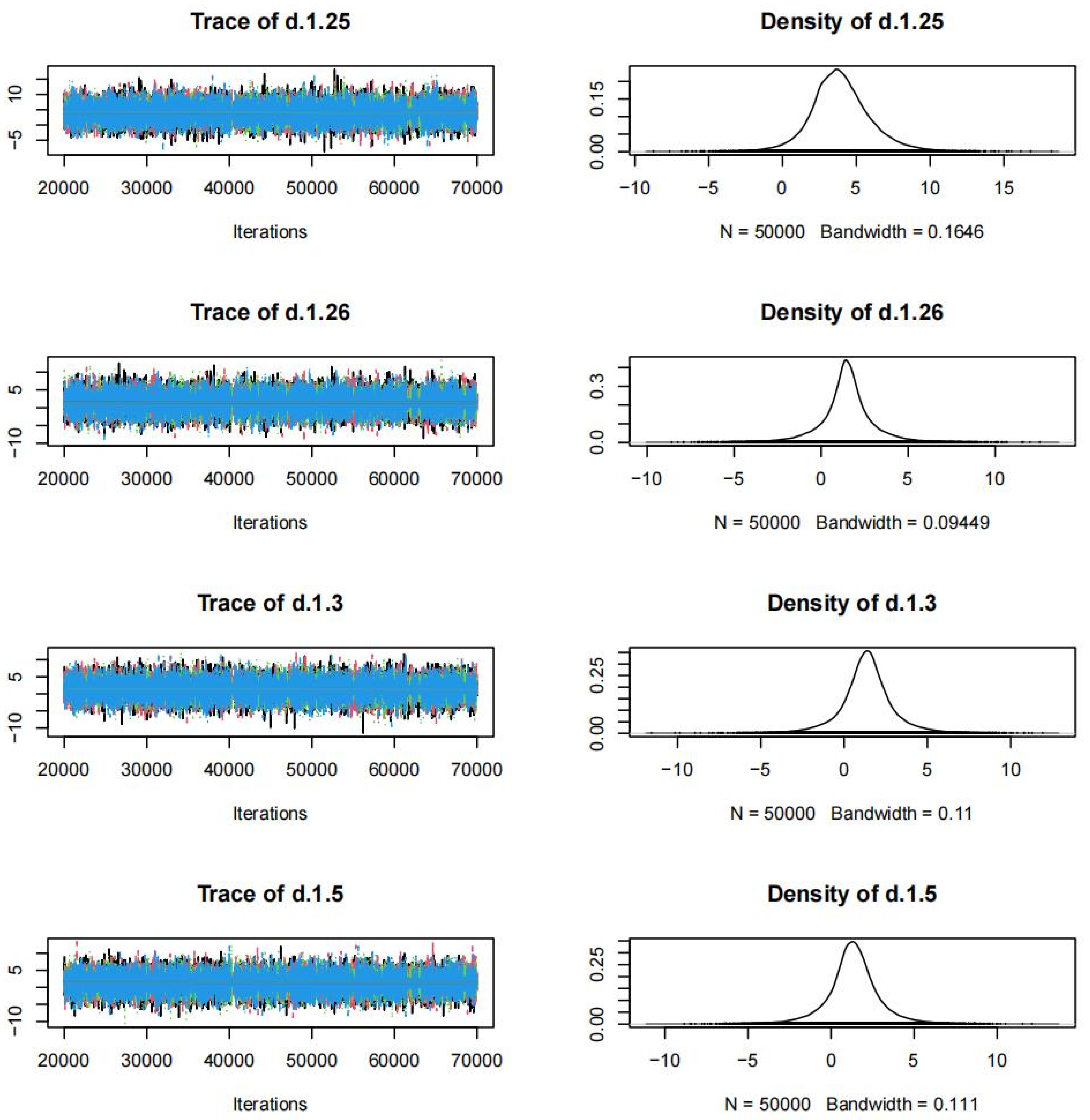
Trace of d.1.25
Iterations
Density of d.1.25
N = 50000 Bandwidth = 0.1646
Trace of d.1.26
Iterations
Density of d.1.26
N = 50000 Bandwidth = 0.09449
Trace of d.1.3
Iterations
Density of d.1.3
N = 50000 Bandwidth = 0.11
Trace of d.1.5
Iterations
Density of d.1.5
N = 50000 Bandwidth = 0.111
