# Supplementary material for: Herbal medicine for asymptomatic hyperuricemia: a systematic review and network meta-analysis
Source: Front Pharmacol. 2025 Sep 29;16:1627714. doi: 10.3389/fphar.2025.1627714 (PMC12515837; doi:10.3389/fphar.2025.1627714)
Supplement: Supplementary file 2 [file Supplementaryfile1.docx]

Appendix 1.General information of included studies.

| Reference | HM formula | Control group | Number of participants R/A | Average age (SD) | HT diagnostic criteria (TCM diagnostic pattern(s), if reported) | Duration of HUA | Treatment duration (Length of follow-up) | Outcome |
| --- | --- | --- | --- | --- | --- | --- | --- | --- |
| P. Dong 2022 | Lily-Plantago Seed tea | Follow-up observation | HM group: 55/55 Control group: 55/55 | HM group: 59.85 (3.13)  Control group: 59.88 (3.19) | Practice Guidelines for the Diagnosis and Treatment of Hyperuricemia in Kidney Diseases in China (2017 edition) (Damp-heat accumulation or phlegm stagnation) | Not reported | 3 months (3 months) | Reduction of SUA Symptom improvement |
| RJ. Liang 2014 | Rhizoma Dioscoreae Hypoglaucae and Glabrous Greenbrier Ghizome Traditional Chinese Medicine granules | Follow-up observation | HM group: 30/30 Control group: 30/30 | HM group: 62.5 (5.4)  Control group: 61.8 (5.7) | Practical Internal Medicine (Phlegm-dampness or pampness-heat) | HM group: 6.5±3.4 months Control group: 6.8±3.7 months | 4 weeks (4 weeks) | Reduction of SUA Symptom improvement |
| BC. Chen 2006 | Xuezhikang capsule | Placebo | HM group: 40/38 Control group: 40/37 | HM group: 35.81 (12.13)  Control group: 35.65 (12.19) | Not reported | Not reported | 4 weeks (4 weeks) | Reduction of SUA, XOD, TC, TG, LP, CRP, ET-1, β2-MG Elevation of HDL |
| LN. Sun 2006 | Strengthening the spleen, dispelling dampness, and relieving turbidity therapy | Follow-up observation | HM group: 35/35 Control group: 35/35 | HM group: 42.03 (10.68)  Control group: 38.60 (10.66) | Internal Medicine (Sixth Edition) | Not reported | 2 months (2 months) | Reduction of SUA, TG Symptom improvement |
| ZJ. Lu 2010 | Spleen Nourishing and Turbidity Relieving Therapy | Follow-up observation | HM group: 72/72 Control group: 72/72 | Not reported | Not reported | Not reported | 2 months (2 months) | Reduction of SUA Vascular endothelial function improvement |
| XX. Chen 2010 | Anoectochilus roxburghii capsule | Placebo | HM group: 36/35 Control group: 33/33 | HM group: 84.73 (4.16)  Control group: 83.56 (3.72) | Practical Internal Medicine (Phlegm-dampness and Dampness-heat) | Not reported | 30 days (30 days) | Reduction of SUA |
| XX. Zhang 2011 | Xiezhuo　Chubi　Formula | Follow-up observation | HM group: 33/28 Control group: 33/30 | HM group: 56.07 (17.62)  Control group: 53.07 (15.76) | Chinese Rheumatology | Not reported | 20 days (20 days) | Reduction of SUA Symptom improvement |
| JH. Peng 2012 | Yunpi xiezhuo Powder | Follow-up observation | HM group: 100/100 Control group: 100/100 | HM group: 41.03 (9.68)  Control group: 40.60 (10.66) | Internal Medicine (Seventh Edition) (Spleen deficiency and dampness accumulation) | HM group: 1.67±0.68 years Control group: 1.55±1.06 years | 3 months (3 months) | Reduction of SUA, GLU, TC, TG, LDL Elevation of HDL |
| H. Xu 2013 | Traditional Chinese medicine uric acid particles | Placebo | HM group: 60/50 Control group: 60/48 | HM group: 51(13.0)  Control group: 52 (14.1) | (Spleen deficiency or damp-heat accumulation) | Not reported | 4 weeks (4 weeks) | Reduction of SUA, LDL Symptom improvement |
| CY. Han 2015 | Zhongyue jiangsangao yaocha | Follow-up observation | HM group: 30/30 Control group: 30/30 | HM group: 49.1 (7.3)  Control group: 49.4 (7.6) | (Qi deficiency with phlegm dampness constitution) | Not reported | 1 month (1 month) | Reduction of SUA |
| HX. Qiao 2016 | Qinluo huazhuo granules | Follow-up observation | HM group: 40/40 Control group: 40/40 | HM group: 67.09 (8.23)  Control group: 63.58 (6.62) | Chinese expert consensus about hyperuricemia and gout treatment | Not reported | 1 month (1 month) | Reduction of SUA, TG |
| H. Sun 2016 | Traditional Chinese medicine formula granules of clear heat evil and detoxification | Placebo | HM group: 60/50 Control group: 60/48 | HM group: 51 (12.0)  Control group: 52 (12.1) | Chinese expert consensus recommendations for diagnosis and treatment of asymptomatic hyperuricemia complicated with cardiovascular diseases (Dampness toxin heat obstruction) | Not reported | 4 weeks (4 weeks) | Reduction of SUA, LDL-C Symptom improvement |
| XX. Zhang 2016a | Compound Tufuling Granules | Follow-up observation | HM group: 30/23 Control group: 30/28 | HM group: 50.17 (15.12) Control group: 53.50 (16.32) | Chinese Rheumatology (Phlegm dampness obstruction) | Not reported | 60 days (60 days) | Reduction of SUA Symptom improvement |
| XX. Zhang 2016b | Compound Tufuling Granules | Follow-up observation | HM group: 20/15 Control group: 20/20 | HM group: 50.17 (15.12) Control group: 53.50 (16.32) | Chinese Rheumatology (Phlegm dampness obstruction) | HM group: 10.63 (8.91) Control group: 8.30 (8.42) | 12 months (12 months) | Reduction of SUA Symptom improvement |
| HL. Liu 2017 | Traditional Chinese medicine uric acid particles | Placebo | HM group: 39/39 Control group: 39/39 | HM group: 52.41 (14.04)  Control group: 55.26 (14.38) | Not reported | Not reported | 4 weeks (4 weeks) | Reduction of SUA Symptom improvement |
| YT. Liu 2017 | Compound Tufuling Granules | Follow-up observation | HM group: 30/30 Control group: 30/30 | HM group: 51.94 (12.62)  Control group: 50.98 (9.29) | American College of Rheumatology Gout Classification Standard (Phlegm dampness) | Not reported | 3 months (3 months) | Reduction of SUA, MCP-1, TNF-α, IL-6, TC, TG, CysC Elevation of APN, NO, HDL-C |
| HX. Huang 2017 | Maxingyigan Decoction | Follow-up observation | HM group: 60/60 Control group: 60/60 | HM group: 38.4 (4.1)  Control group: 38.3 (4.2) | Internal Medicine (Third Edition) (Phlegm dampness) | HM group: 6.5±2.1 months Control group: 6.4±2.0 months | 30 days (30 days) | Reduction of SUA, TC, TG, LDL-C Elevation of HDL-C Symptom improvement |
| LY. Zhang 2017 | Lingyu Qingluo Decoction | Follow-up observation | HM group: 30/30 Control group: 30/30 | HM group: 56.57 (10.33)  Control group: 56.67 (11.82) | Internal Medicine (Eighth Edition) (Phlegm dampness) | Not reported | 1 month (1 month) | Reduction of SUA, TC, TG, hs-CRP |
| HX. Huang 2018 | Maxing Yigan decoction | Follow-up observation | HM group: 60/60 Control group: 60/60 | HM group: 35.8 (11.7)  Control group: 34.7 (11.4) | Chinese expert consensus about hyperuricemia and gout treatment (Phlegm dampness) | Not reported | 30 days (30 days) | Reduction of SUA Symptom improvement |
| CE. Yu 2018 | Jianpi Shen Xie Zhuo Ointment | Follow-up observation | HM group: 40/40 Control group: 40/40 | HM group: 50.31 (12.18)  Control group: 51.32 (11.73) | Chinese expert consensus recommendations for diagnosis and treatment of asymptomatic hyperuricemia complicated with cardiovascular diseases | HM group: 4.62±2.16 months Control group: 4.71±2.02 months | 16 weeks (16 weeks) | Reduction of SUA Symptom improvement |
| M. Liang 2018 | Guben Xiezhuo prescription | Follow-up observation | HM group: 30/30 Control group: 30/30 | HM group: 46.8 (16.58)  Control group: 55.16 (19.01) | Internal Medicine (Eighth Edition) (Spleen and kidney deficiency or Damp-turbid internal retention) | Not reported | 8 weeks (8 weeks) | Reduction of SUA Symptom improvement |
| JY Lu 2019 | Modified Wuling Powder | Follow-up observation | HM group: 30/30 Control group: 30/30 | HM group: 53.60 (14.65)  Control group: 54.40 (13.85) | China multi-disciplinary expert consensus on diagnosis and treatment of hyperuricemia and related diseases (2017 edition) (damp-heat accumulation) | Not reported | 12 weeks (12 weeks) | Reduction of SUA, BMI, FPG, 2hPG, TC, TG, LDL-C, BUN, Scr, ADA, XOD Elevation of HDL-C Symptom improvement |
| JL. Hao 2020 | Qingrelishixiezhuo Method | Follow-up observation | HM group: 30/30 Control group: 30/30 | HM group: 44.37 (11.67)  Control group: 45.00 (11.49) | Chinese expert consensus about hyperuricemia and gout treatment (damp-heat accumulation) | Not reported | 8 weeks (8 weeks) | Reduction of SUA, TG Elevation of NO Symptom improvement |
| L. Zhang 2020 | Shenchayin | Follow-up observation | HM group: 36/32 Control group: 36/29 | Not reported | Chinese expert consensus about hyperuricemia and gout treatment (damp-heat) | Not reported | 3 months (3 months) | Reduction of SUA, TC, ALT, AST, Cr Elevation of NO Symptom improvement |
| WT. Li 2021 | Qingre Lishi Recipe | Follow-up observation | HM group: 35/31 Control group: 35/34 | HM group: 46.29 (16.08)  Control group: 45.97 (12.83) | China multi-disciplinary expert consensus on diagnosis and treatment of hyperuricemia and related diseases (2017 edition) (Dampness heat stagnation in the spleen) | HM group: 33.68 months Control group: 32.38 months | 4 weeks (4 weeks) | Reduction of SUA, URAT1, TC, TG, LDL Symptom improvement |
| ZD.Guo 2021 | YiShenJianPiHuaZhuo decoction | Follow-up observation | HM group: 33/32 Control group: 35/35 | HM group: 42.78 (14.08)  Control group: 41.97 (12.93) | Guideline for the diagnosis and management of hyperuricemia and gout in China (2019 edition) (Symptoms of spleen and kidney deficiency with damp pathogen) | Not reported | 8 weeks (8 weeks) | Reduction of SUA, HX, TC, LDL-C Elevation of HDL-C Symptom improvement |
| F. Shen 2021 | Green barley | Follow-up observation | HM group: 32/32 Control group: 25/25 | HM group: 45.92 (12.68)  Control group: 47.90 (8.83) | Not reported | Not reported | 90 days (90 days) | Reduction of SUA Elevation of HDL-C |
| JY. Liang 2021 | Huazhuojiedu prescription | Follow-up observation | HM group: 30/30 Control group: 30/30 | HM group: 37.50 Control group: 36.50 | Guideline for the diagnosis and management of hyperuricemia and gout in China (2019 edition) (Turbid-toxic internal retention) | Not reported | 4 weeks (4 weeks) | Reduction of SUA, BMI Symptom improvement |
| DX. Zhang 2021 | Substituting tea drinking for dampness-removing and turbidity-resolving | Follow-up observation | HM group: 84/84 Control group: 84/84 | HM group: 40.5 (6.1)  Control group: 41.7 (5.8) | Chinese expert consensus about hyperuricemia and gout treatment (Phlegm dampness) | Not reported | 6 months (6 months) | Reduction of SUA Symptom improvement |
| GR. Shao 2022 | Lishi Qingre Decoction | Follow-up observation | HM group: 30/30 Control group: 30/30 | HM group: 40.83 (13.50)  Control group: 43.87 (13.68) | Guideline for the diagnosis and management of hyperuricemia and gout in China (2019 edition) (Damp-heat accumulation) | Not reported | 4 weeks (4 weeks) | Reduction of SUA; Hcy; TG Symptom improvement |
