# Supplementary material for: Herbal medicine for asymptomatic hyperuricemia: a systematic review and network meta-analysis
Source: Front Pharmacol. 2025 Sep 29;16:1627714. doi: 10.3389/fphar.2025.1627714 (PMC12515837; doi:10.3389/fphar.2025.1627714)
Supplement: Supplementary file 3 [file Supplementaryfile7.pdf]

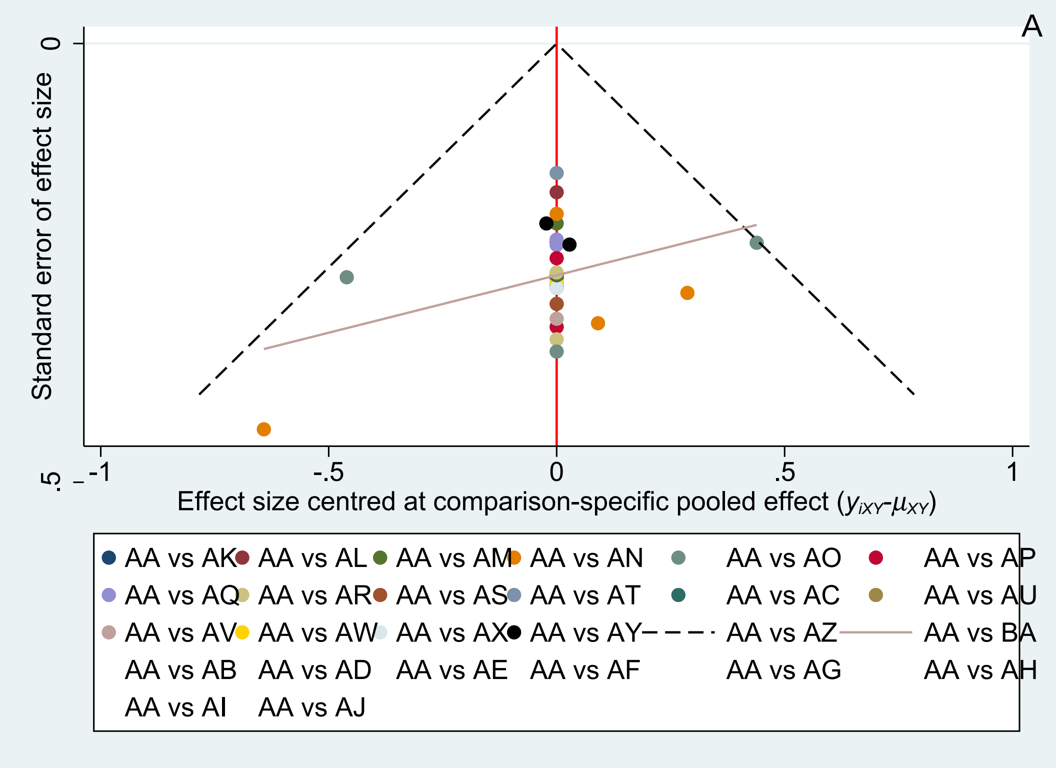

A
Standard error of effect size
0
.5
-1
-.5
0
.5
1
Effect size centred at comparison-specific pooled effect (y_{iXY}-μ_{XY})
AA vs AK
AA vs AL
AA vs AM
AA vs AN
AA vs AO
AA vs AP
AA vs AQ
AA vs AR
AA vs AS
AA vs AT
AA vs AC
AA vs AU
AA vs AV
AA vs AW
AA vs AX
AA vs AY
AA vs AZ
AA vs BA
AA vs AB
AA vs AD
AA vs AE
AA vs AF
AA vs AG
AA vs AH
AA vs AI
AA vs AJ

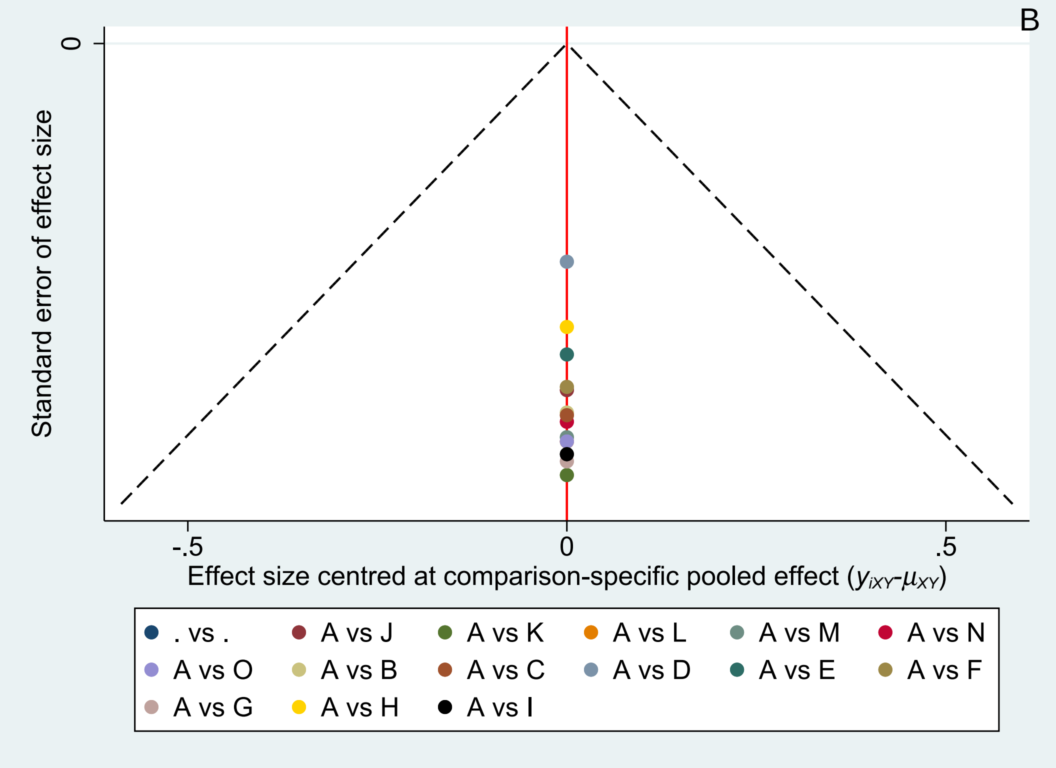
B
0
Standard error of effect size
-.5
0
.5
Effect size centred at comparison-specific pooled effect (y_{iXY}-μ_{XY})
. vs .
A vs J
A vs K
A vs L
A vs M
A vs N
A vs O
A vs B
A vs C
A vs D
A vs E
A vs F
A vs G
A vs H
A vs I

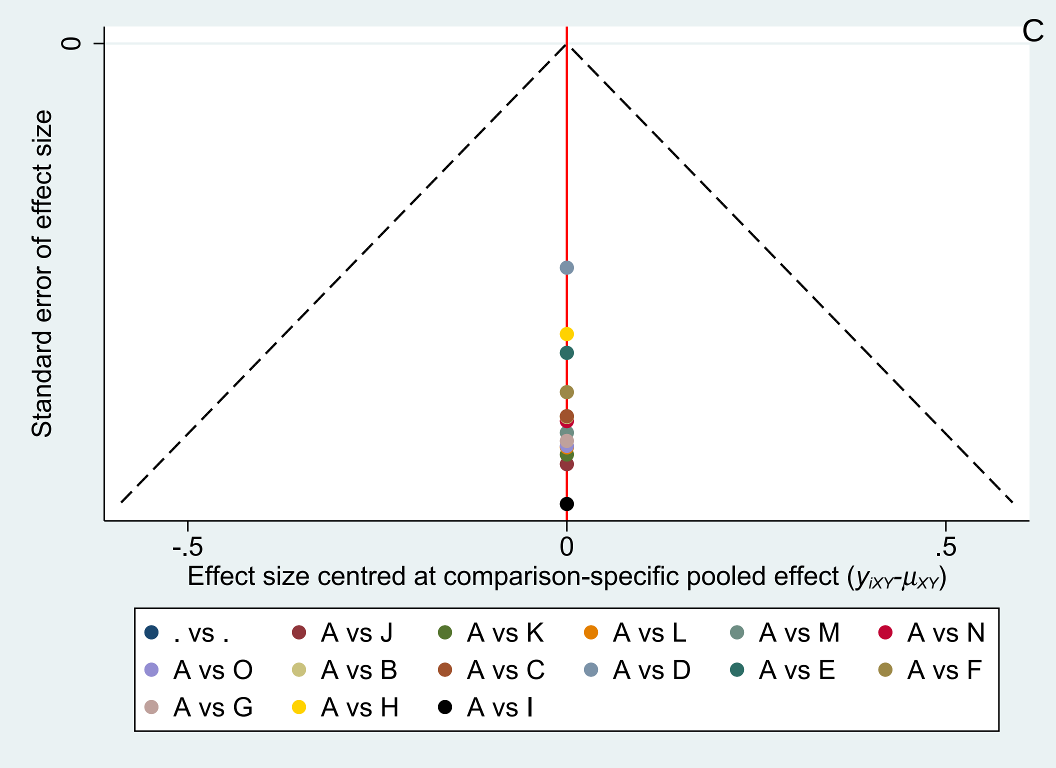
C
Standard error of effect size
0
-.5
0
.5
Effect size centred at comparison-specific pooled effect (y_{iXY}-μ_{XY})
. vs .
A vs J
A vs K
A vs L
A vs M
A vs N
A vs O
A vs B
A vs C
A vs D
A vs E
A vs F
A vs G
A vs H
A vs I

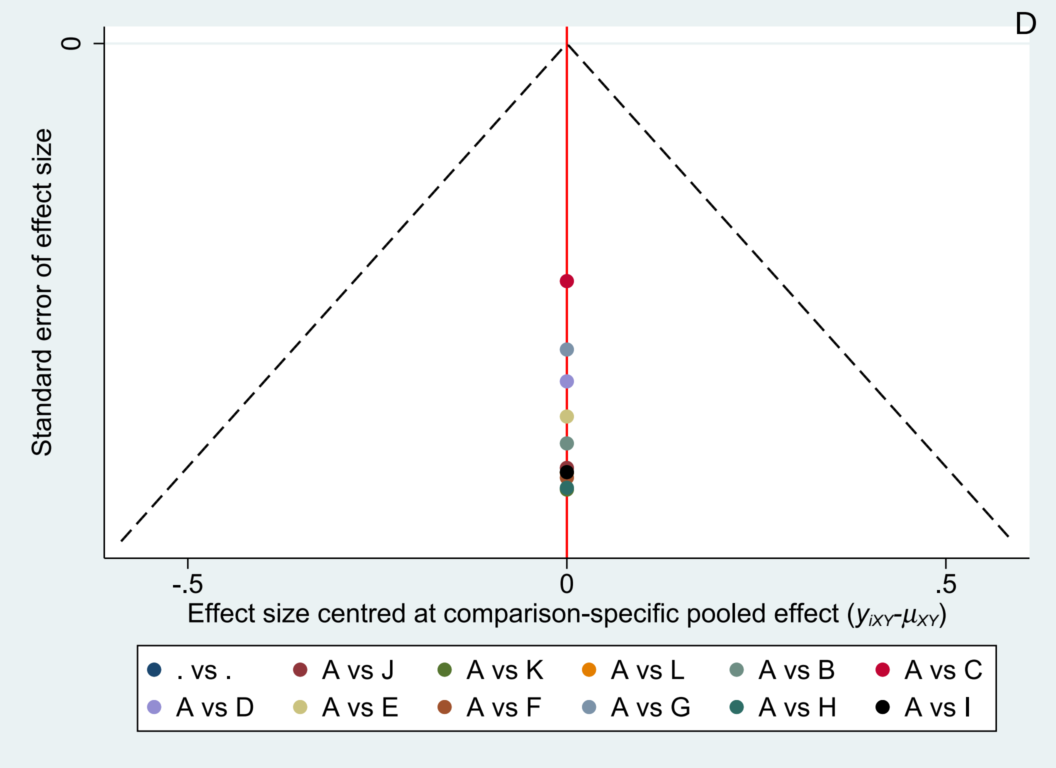
D
0
Standard error of effect size
-.5
0
.5
Effect size centred at comparison-specific pooled effect (y_{iXY}-μ_{XY})
. vs .
A vs J
A vs K
A vs L
A vs B
A vs C
A vs D
A vs E
A vs F
A vs G
A vs H
A vs I

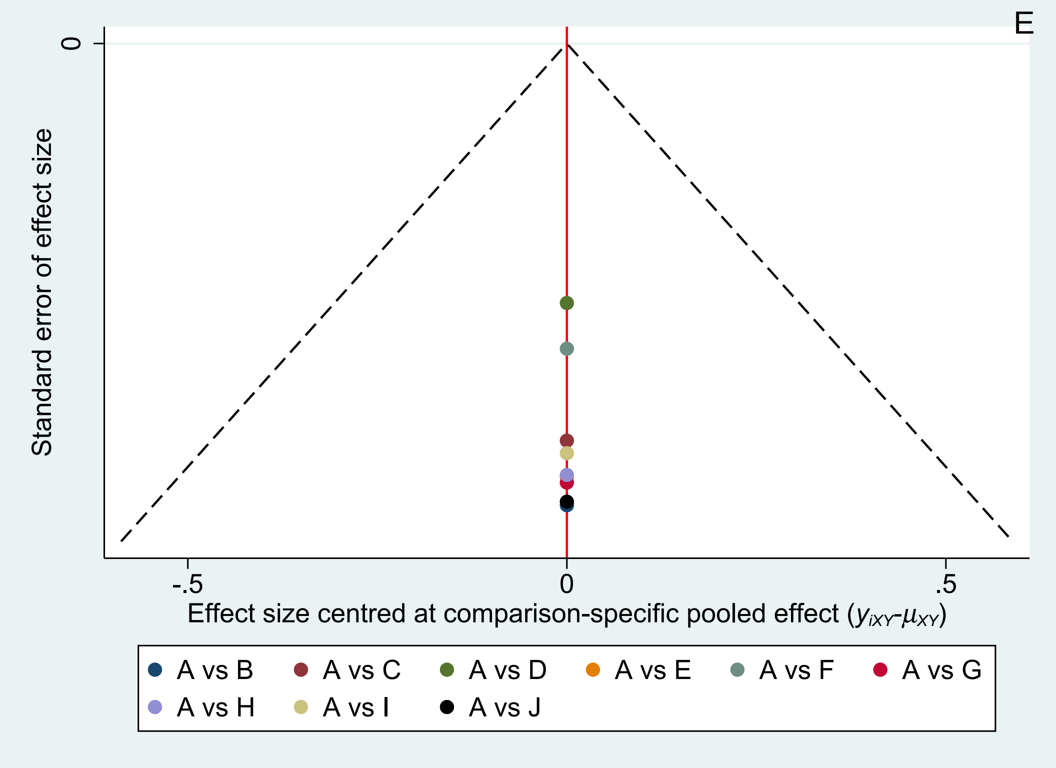

E
Standard error of effect size
0
-.5
0
.5
Effect size centred at comparison-specific pooled effect (y_{iXY}-μ_{XY})
A vs B
A vs C
A vs D
A vs E
A vs F
A vs G
A vs H
A vs I
A vs J

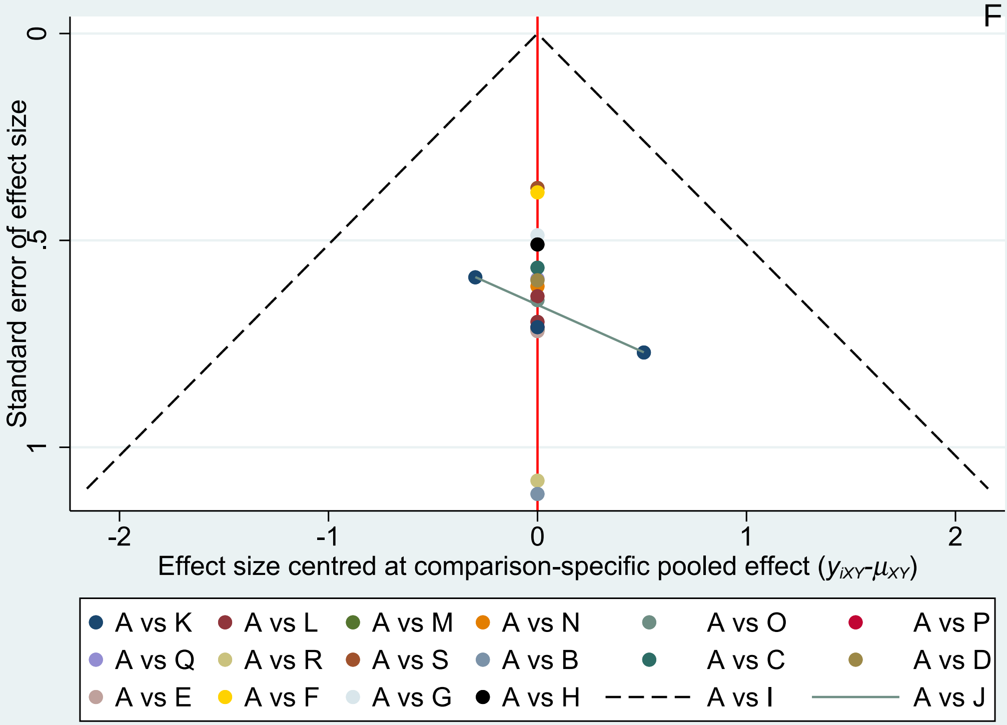
F
Standard error of effect size
0
.5
1
-2
-1
0
1
2
Effect size centred at comparison-specific pooled effect (y_{iXY}-μ_{XY})
A vs K
A vs L
A vs M
A vs N
A vs O
A vs P
A vs Q
A vs R
A vs S
A vs B
A vs C
A vs D
A vs E
A vs F
A vs G
A vs H
A vs I
A vs J
