# Supplementary material for: Herbal medicine for asymptomatic hyperuricemia: a systematic review and network meta-analysis
Source: Front Pharmacol. 2025 Sep 29;16:1627714. doi: 10.3389/fphar.2025.1627714 (PMC12515837; doi:10.3389/fphar.2025.1627714)
Supplement: Supplementary file 4 [file Supplementaryfile2.docx]

Appendix 2. Characteristics of included interventions.

| Reference | HM formula (dosage, frequency) | TCM function(s) of HM formula (if reported) | Ingredients of HM formula | Non-drug therapy (dosage, frequency, if reported) | Adverse event |
| --- | --- | --- | --- | --- | --- |
| P. Dong 2022 | Lily-Plantago Seed tea (13g, twice per day) | Not reported | Lilium lancifolium Thunb., Plantago asiatica L. | Lifestyle intervention: restricting the intake of high purine foods, persist in drinking 2500ml of water every day and exercising more, no alcohol | No adverse events reported |
| RJ. Liang 2014 | Rhizoma Dioscoreae Hypoglaucae and Glabrous Greenbrier Ghizome Traditional Chinese Medicine granules (20g, once per day) | Not reported | Smilax glabra Roxb., Dioscorea collettii var. hypoglauca (Palib.) S.J.Pei & C.T.Ting | Diet controlling | No adverse events reported |
| BC. Chen 2006 | Xuezhikang capsule (0.3 g/capsule, Produced by Beijing Peking University Weixin Biotechnology Co., Ltd) (2 pills,twice per day) | Not reported | Monascus purpureus Went. | Placebo (2 pills, twice per day) | No adverse events reported |
| LN. Sun 2006 | Strengthening the spleen, dispelling dampness, and relieving turbidity therapy (200ml, twice per day) | Promoting diuresis and dampness, invigorating spleen and removing rheumatism | Coix lacryma-jobi var. ma-yuen (Rom.Caill.) Stapf, Smilax glabra Roxb., Clematis chinensis Osbeck, Chaenomeles speciosa (Sweet) Nakai, Dioscorea collettii var. hypoglauca (Palib.) S.J.Pei & C.T.Ting, Cremastra appendiculata (D.Don) Makino | Lifestyle intervention: restricting the intake of high purine foods, no alcohol, maintain a low-fat, low salt, and low sugar diet, drinking sufficient water | All patients did not experience any discomfort, and 2 cases in the control group experienced acute gout attacks during the observation period |
| ZJ. Lu 2010 | Spleen Nourishing and Turbidity Relieving Therapy (10g, twice per day) | Spleen Nourishing and Turbidity Relieving | Coix lacryma-jobi var. ma-yuen (Rom.Caill.) Stapf, Smilax glabra Roxb., Atractylodes macrocephala Koidz., Salvia miltiorrhiza Bunge, Raphanus raphanistrum subsp. sativus (L.) Domin, Chaenomeles speciosa (Sweet) Nakai, Dioscorea collettii var. hypoglauca (Palib.) S.J.Pei & C.T.Ting | Lifestyle intervention: restricting the intake of high purine foods, no smoking and alcohol, drink sufficient water, doing more than 30 minutes of aerobic exercise every day | No adverse events reported |
| XX. Chen 2010 | Anoectochilus roxburghii capsule (3 pills,twice per day) | Not reported | Anoectochilus roxburghii (Wall.) Lindl. | Placebo (3 pills, twice per day) | Two patients in the HM group showed mild elevation of transaminase |
| XX. Zhang 2011 | Xiezhuo Chubi Formula (twice per day) | clearing heat, draining dampness and removing turbidity | Smilax glabra Roxb., Dioscorea collettii var. hypoglauca (Palib.) S.J.Pei & C.T.Ting, Cremastra appendiculata (D.Don) Makino, Gypsophila vaccaria (L.) Sm., Achyranthes bidentata Blume | Health education, restricting the intake of high purine foods, no alcohol | No adverse events reported |
| JH. Peng 2012 | Yunpi xiezhuo Powder (One dose per day) | Strengthening the spleen, removing dampness and turbidity | Coix lacryma-jobi var. ma-yuen (Rom.Caill.) Stapf, Smilax glabra Roxb., Atractylodes macrocephala Koidz., Salvia miltiorrhiza Bunge, Raphanus raphanistrum subsp. sativus (L.) Domin, Chaenomeles speciosa (Sweet) Nakai, Dioscorea collettii var. hypoglauca (Palib.) S.J.Pei & C.T.Ting | Lifestyle intervention: restricting the intake of high purine foods, no smoking and alcohol, persist in drinking 2000ml of water every day, doing more than 30 minutes of aerobic exercise every day | No adverse events reported |
| H. Xu 2013 | Traditional Chinese Medicine Niao SuanLi Xian Formula Granules (1 pack, twice per day) | Strengthening the spleen and kidney, nourishing qi and promoting blood circulation, dispelling wind, dampness, and turbidity | Astragalus mongholicus Bunge, Smilax glabra Roxb., Coix lacryma-jobi var. ma-yuen (Rom.Caill.) Stapf, Cremastra appendiculata (D.Don) Makino, Gentiana macrophylla Pall., Taxillus chinensis (DC.) Danser, Salvia miltiorrhiza Bunge | Placebo (1 pack, twice per day) | "One patient in the HM group experienced lower back pain and one patient experienced oral ulcers |
| CY. Han 2015 | Zhongyue jiangsangao yaocha (5~10g, once per day) | Not reported | Crataegus pinnatifida Bunge, Senna tora (L.) Roxb., Styphnolobium japonicum (L.) Schott, Lycium barbarum L., Polygonatum sibiricum Redouté, Pueraria montana var. lobata (Willd.) Maesen & S.M.Almeida ex Sanjappa & Predeep, Ziziphus jujuba Mill., Dioscorea oppositifolia L., Prunus mume (Siebold) Siebold & Zucc., Glycyrrhiza glabra L. | Lifestyle intervention: eat more alkaline foods and less acidic foods, no alcohol, drink sufficient water and maintain a daily urine output of > 2000 mL | One patient in the control group experienced stomach discomfort" |
| HX. Qiao 2016 | Qinluo huazhuo Formula (1 pack, twice a day) | Invigorating spleen and relieving kidney, tonifying qi and resolving turbidity | Fraxinus chinensis Roxb., Trachelospermum jasminoides (Lindl.) Lem., Astragalus mongholicus Bunge, Atractylodes macrocephala Koidz., Synonym of Smyrnium L., Alisma plantago-aquatica L., Salvia miltiorrhiza Bunge, Coix lacryma-jobi var. ma-yuen (Rom.Caill.) Stapf, Paeonia × suffruticosa Andrews, Conioselinum anthriscoides 'Chuanxiong' | Lifestyle intervention: restricting the intake of high purine foods, no smoking and alcohol, drink sufficient water and maintain a daily urine output of > 1500 mL, health education | No adverse events reported |
| H. Sun 2016 | Traditional Chinese medicine (TCM) formula granules of clear heat evil and detoxification (Contains 50 g/pack of raw medicine) (1 pack, twice per day) | Clearing heat, detoxifying, and removing dampness | Smilax glabra Roxb., Cremastra appendiculata (D.Don) Makino, Lysimachia christinae Hance, Glechoma longituba (Nakai) Kuprian., Citrus × aurantium L. | Placebo (1 pack, twice per day) | No adverse events reported |
| XX. Zhang 2016a | Compound Tufuling Granules (10g, twice per day) | Clearing heat, promoting diuresis, and reducing turbidity | Smilax glabra Roxb., Dioscorea collettii var. hypoglauca (Palib.) S.J.Pei & C.T.Ting, Cremastra appendiculata (D.Don) Makino, Gypsophila vaccaria (L.) Sm., Achyranthes bidentata Blume | Lifestyle intervention: restricting the intake of high purine foods, no alcohol, drink sufficient water, health education | "One patient in the HM group experienced lower back pain |
| XX. Zhang 2016b | Compound Tufuling Granules (10g, twice per day) | Relieving turbidity and eliminating evil, dispelling dampness and clearing heat, promoting blood circulation and removing blood stasis | Smilax glabra Roxb., Dioscorea collettii var. hypoglauca (Palib.) S.J.Pei & C.T.Ting, Cremastra appendiculata (D.Don) Makino, Gypsophila vaccaria (L.) Sm., Achyranthes bidentata Blume | Lifestyle intervention: restricting the intake of high purine foods, no alcohol, drink sufficient water, health education | One patient in the control group experienced stomach discomfort" |
| HL. Liu 2017 | Traditional Chinese medicine (TCM) uric acid particles (One dose per day) | Invigorating spleen and kidney, invigorating qi and activating blood circulation, dissipating phlegm and removing blood stasis | Astragalus mongholicus Bunge, Smilax glabra Roxb., Coix lacryma-jobi var. ma-yuen (Rom.Caill.) Stapf, Cremastra appendiculata (D.Don) Makino, Gentiana macrophylla Pall., Taxillus chinensis (DC.) Danser, Salvia miltiorrhiza Bunge | Placebo (One dose per day) | No adverse events reported |
| YT. Liu 2017 | Compound Tufuling Granules (10g, twice per day) | Clearing heat and dampness, relieving turbidity and detoxification, resolving phlegm and dispersing blood stasis | Smilax glabra Roxb., Dioscorea collettii var. hypoglauca (Palib.) S.J.Pei & C.T.Ting, Cremastra appendiculata (D.Don) Makino, Gypsophila vaccaria (L.) Sm., Achyranthes bidentata Blume | Lifestyle intervention: restricting the intake of high purine foods, no alcohol, persist in drinking 1500ml of water every day (maintain a daily urine output of > 2000 mL), doing more than 30 minutes of aerobic exercise every day | No adverse events reported |
| HX. Huang 2017 | Maxingyigan Decoction (One dose per day) | Not reported | Ephedra sinica Stapf, Prunus sibirica L., Coix lacryma-jobi var. ma-yuen (Rom.Caill.) Stapf, Glycyrrhiza glabra L. | Lifestyle intervention: restricting the intake of high purine foods, no smoking and limit alcohol consumption, doing more than 30 minutes of aerobic exercise 5 times per week | No adverse events reported |
| LY. Zhang 2017 | Lingyu Qingluo Decoction (One dose per day) | Clearing heat and turbidity, promoting blood circulation and unblocking collaterals | Smilax glabra Roxb., Zea mays L., Imperata cylindrica (L.) Raeusch., Coix lacryma-jobi var. ma-yuen (Rom.Caill.) Stapf, Salvia miltiorrhiza Bunge, Reynoutria japonica Houtt. | Lifestyle intervention: restricting the intake of high purine foods, no alcohol, drink sufficient water, health education | No adverse events reported |
| HX. Huang 2018 | Maxingyigan Decoction (One dose per day) | Not reported | Ephedra sinica Stapf, Prunus sibirica L., Coix lacryma-jobi var. ma-yuen (Rom.Caill.) Stapf, Glycyrrhiza glabra L. | Lifestyle intervention: recommend low purine foods and moderate aerobic exercise | No adverse events reported |
| CE. Yu 2018 | Jianpi Shen Xie Zhuo Ointment (25g, twice per day) | Strengthening the spleen and benefiting the kidneys, promoting dampness, promoting blood circulation, and purging turbidity | Astragalus mongholicus Bunge, Smilax glabra Roxb., Codonopsis pilosula (Franch.) Nannf., Cuscuta chinensis Lam., Morus alba L., Reynoutria multiflora (Thunb.) Moldenke, Curcuma longa L., Prunus persica (L.) Batsch, Angelica sinensis (Oliv.) Diels, Chinemys reevesii(Gray), Polyporus umbellatus (Pers.) Fries, Atractylodes lancea (Thunb.) DC., Phellodendron chinense C.K.Schneid., Gynostemma pentaphyllum (Thunb.) Makino, Cervus elaphus Linnaeus, Wurfbainia villosa (Lour.) Škorničk. & A.D.Poulsen, Dioscorea collettii var. hypoglauca (Palib.) S.J.Pei & C.T.Ting, Gentiana macrophylla Pall., Crataegus pinnatifida Bunge, Alisma plantago-aquatica L., Achyranthes bidentata Blume, Reynoutria japonica Houtt., Atractylodes macrocephala Koidz., Clematis chinensis Osbeck, Citrus × aurantium L. | Lifestyle intervention: restricting the intake of high purine foods, no smoking and alcohol, persist in drinking 1500ml of water every day, regulating emotions | No adverse events reported |
| M. Liang 2018 | Guben Xiezhuo prescription (One dose per day) | Strengthening the spleen and warming the kidneys, relieving dampness and turbidity | Astragalus mongholicus Bunge, Atractylodes macrocephala Koidz., Epimedium brevicornu Maxim., Smilax glabra Roxb., Clematis chinensis Osbeck, Morus alba L., Rheum palmatum L. | Lifestyle intervention: restricting the intake of high purine foods, no smoking and alcohol, drink sufficient water and maintain a daily urine output of 1500~2000mL, regulating emotions | One patient in the HM group experienced abnormal transaminase and one patient experienced stomach discomfort |
| JY Lu 2019 | Modified Wuling Powder (One grid , twice per day) | Clearing dampness and heat | Polyporus umbellatus (Pers.) Fries, Synonym of Smyrnium L., Alisma plantago-aquatica L., Dioscorea collettii var. hypoglauca (Palib.) S.J.Pei & C.T.Ting, Atractylodes macrocephala Koidz., Neolitsea cassia (L.) Kosterm., Plantago asiatica L., Salvia miltiorrhiza Bunge, Rheum palmatum L. | Lifestyle intervention: dietary control, moderate and equal amount of exercise | No adverse events reported |
| JL. Hao 2020 | Qingrelishixiezhuo Method (1 pack, twice a day) | Clear heat and detoxify, dissolve dampness and relieve turbidity | Gypsum, Anemarrhena asphodeloides Bunge, Phellodendron chinense C.K.Schneid., Dioscorea collettii var. hypoglauca (Palib.) S.J.Pei & C.T.Ting, Plantago asiatica L., Synonym of Smyrnium L., Dioscorea oppositifolia L., Atractylodes macrocephala Koidz., Acorus calamus var. angustatus Besser, Nelumbo nucifera Gaertn., Salvia miltiorrhiza Bunge, Glycyrrhiza glabra L. | Lifestyle intervention: restricting the intake of high purine foods, no alcohol, persist in drinking ＞1500ml of water every day, regulating emotions, light to moderate exercise for 30 minutes to 1 hour every day | No adverse events reported |
| L. Zhang 2020 | Shenchayin (4.5g, twice a day) | Clearing heat and promoting dampness, removing stones and reducing turbidity | Orthosiphon aristatus var. aristatus, Lophatherum gracile Brongn., Lonicera japonica Thunb., Atractylodes macrocephala Koidz., Imperata cylindrica (L.) Raeusch. | Lifestyle intervention: restricting the intake of high purine foods, no smoking and alcohol, health education | No adverse events reported |
| WT. Li 2021 | Qingre Lishi Recipe (One dose per day) | Clearing heat and promoting dampness | Phellodendron chinense C.K.Schneid., Rehmannia glutinosa (Gaertn.) DC., Plantago asiatica L., Achyranthes bidentata Blume, Alisma plantago-aquatica L., Angelica sinensis (Oliv.) Diels, Synonym of Smyrnium L., Paeonia × suffruticosa Andrews, Lonicera japonica Thunb., Scrophularia ningpoensis Hemsl., Paeonia lactiflora Pall., Coix lacryma-jobi var. ma-yuen (Rom.Caill.) Stapf, Cremastra appendiculata (D.Don) Makino, Smilax glabra Roxb., Stephania tetrandra S.Moore, Glycyrrhiza glabra L. | Lifestyle intervention: restricting the intake of high purine foods, drink sufficient water and maintain a daily urine output of 2000~3000mL, regulating emotions, moderate and equal amount of exercise | No adverse events reported |
| ZD.Guo 2021 | YiShenJianPiHuaZhuo decoction (150ml, twice per day) | Invigorate the kidney and spleen and resolving Turbidity | Reynoutria multiflora (Thunb.) Moldenke, Ligustrum lucidum W.T.Aiton, Achyranthes bidentata Blume, Taxillus chinensis (DC.) Danser, Atractylodes macrocephala Koidz., Atractylodes lancea (Thunb.) DC., Smilax glabra Roxb., Coix lacryma-jobi var. ma-yuen (Rom.Caill.) Stapf, Phellodendron chinense C.K.Schneid., silkworm excrement, Neolitsea cassia (L.) Kosterm., Cremastra appendiculata (D.Don) Makino | Lifestyle intervention: restricting the intake of high purine foods, drink sufficient water and maintain a daily urine output of ＞2000ml, regulating emotions, moderate and equal amount of exercise | No adverse events reported |
| F. Shen 2021 | Green barley (Contains 500mg/pill) (10 pill, third per day) | Not reported | Hordeum vulgare L. | Balanced dietary pattern | No adverse events reported |
| JY. Liang 2021 | Huazhuojiedu prescription (One dose per day) | Dissolve turbidity and detoxify | Bupleurum chinense DC., Scutellaria baicalensis Georgi, Eupatorium fortunei Turcz., Citrus × aurantium L., Paeonia lactiflora Pall., Zingiber officinale Roscoe, Coptis chinensis Franch., Pinellia ternata (Thunb.) Makino, Bombyx moriLinnaeus, Cryptotympanapustulata Fabricius, Curcuma longa L., Rheum palmatum L., Gynostemma pentaphyllum (Thunb.) Makino, Glycyrrhiza glabra L. | Lifestyle intervention: restricting the intake of high purine foods, maintain a daily water intake of ＞2000ml | No adverse events reported |
| DX. Zhang 2021 | Substituting tea drinking for dampness-removing and turbidity-resolving (Contains 101 g/pack of raw medicine) (1 pack, third per day) | Strengthening the spleen and benefiting qi, promoting diuresis and removing turbidity | Synonym of Smyrnium L., Atractylodes macrocephala Koidz., Alisma plantago-aquatica L., Coix lacryma-jobi var. ma-yuen (Rom.Caill.) Stapf, Crataegus pinnatifida Bunge, Citrus reticulata Blanco | Lifestyle intervention: restricting the intake of high purine foods | No adverse events reported |
| GR. Shao 2022 | Lishi Qingre Decoction (200ml, twice per day) | Promoting dampness and clearing heat, invigorating the spleen and tonifying qi | Lysimachia christinae Hance, Phellodendron chinense C.K.Schneid., Atractylodes lancea (Thunb.) DC., Cyathula officinalis K.C.Kuan, Coix lacryma-jobi var. ma-yuen (Rom.Caill.) Stapf, Synonym of Smyrnium L., Alisma plantago-aquatica L., Atractylodes macrocephala Koidz., Smilax glabra Roxb., Plantago asiatica L., Dioscorea collettii var. hypoglauca (Palib.) S.J.Pei & C.T.Ting, Dioscorea nipponica Makino | Lifestyle intervention: restricting the intake of high purine foods; maintain a daily water intake of ＞2000ml | No adverse events reported |
