# Supplementary figures and images for: Herbal medicine for asymptomatic hyperuricemia: a systematic review and network meta-analysis
Source: Front Pharmacol. 2025 Sep 29;16:1627714. doi: 10.3389/fphar.2025.1627714 (PMC12515837; doi:10.3389/fphar.2025.1627714)

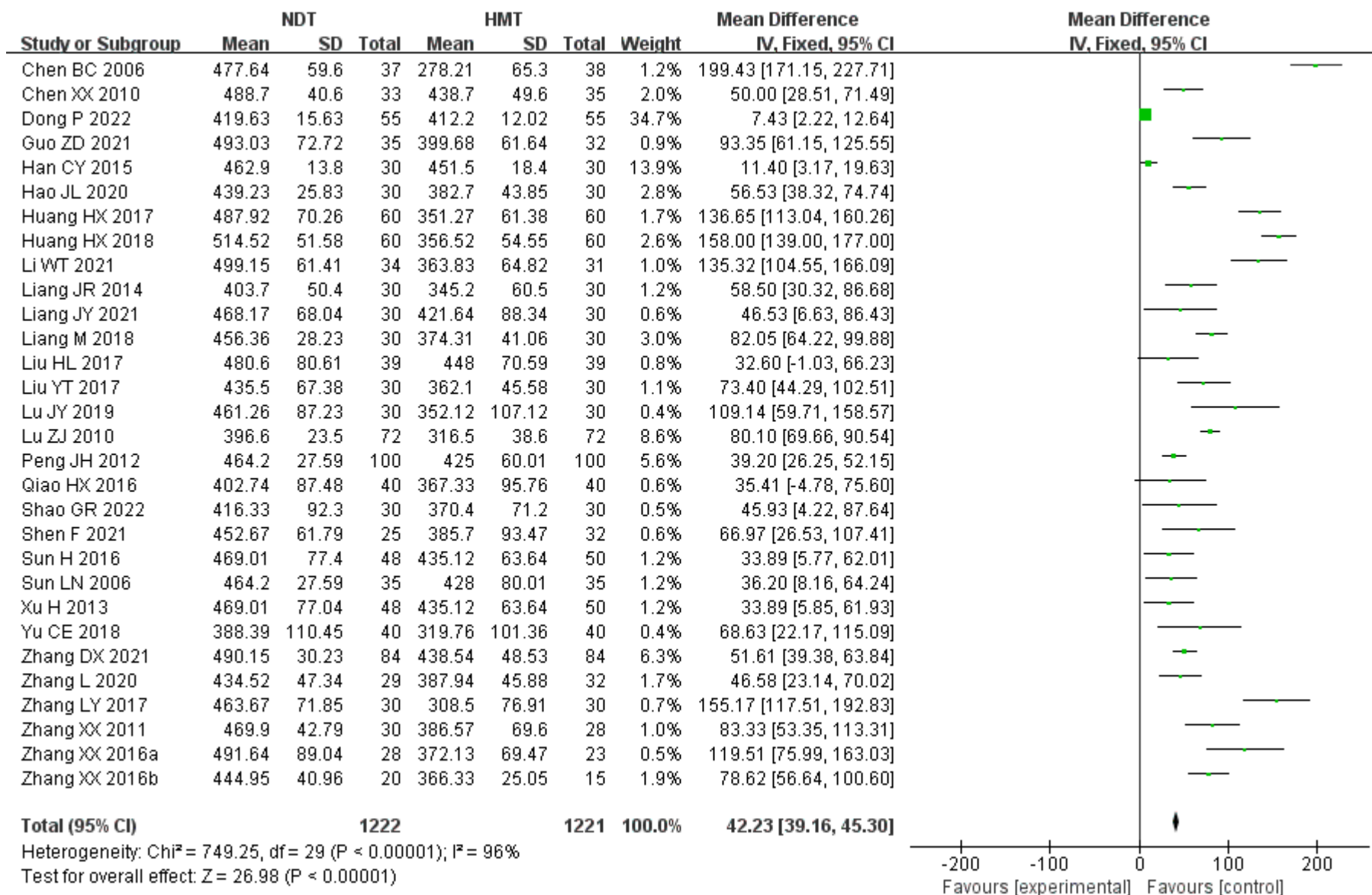

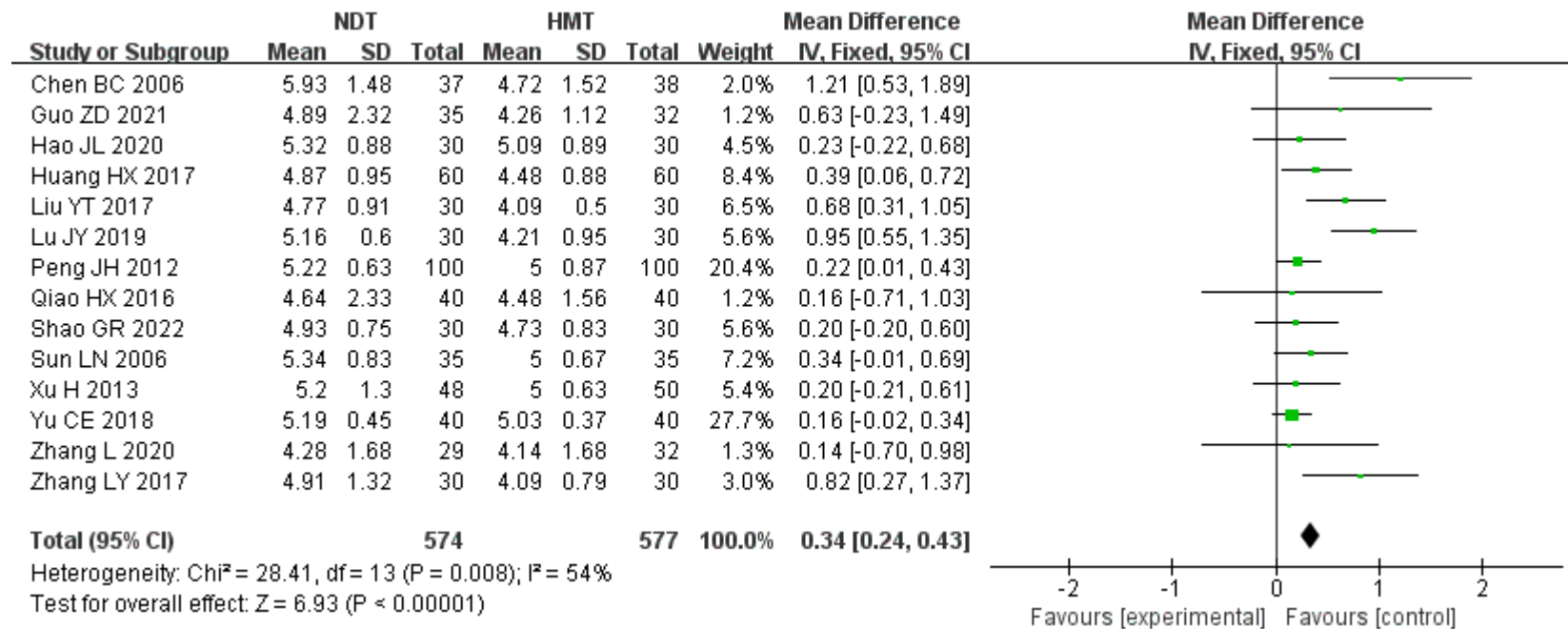

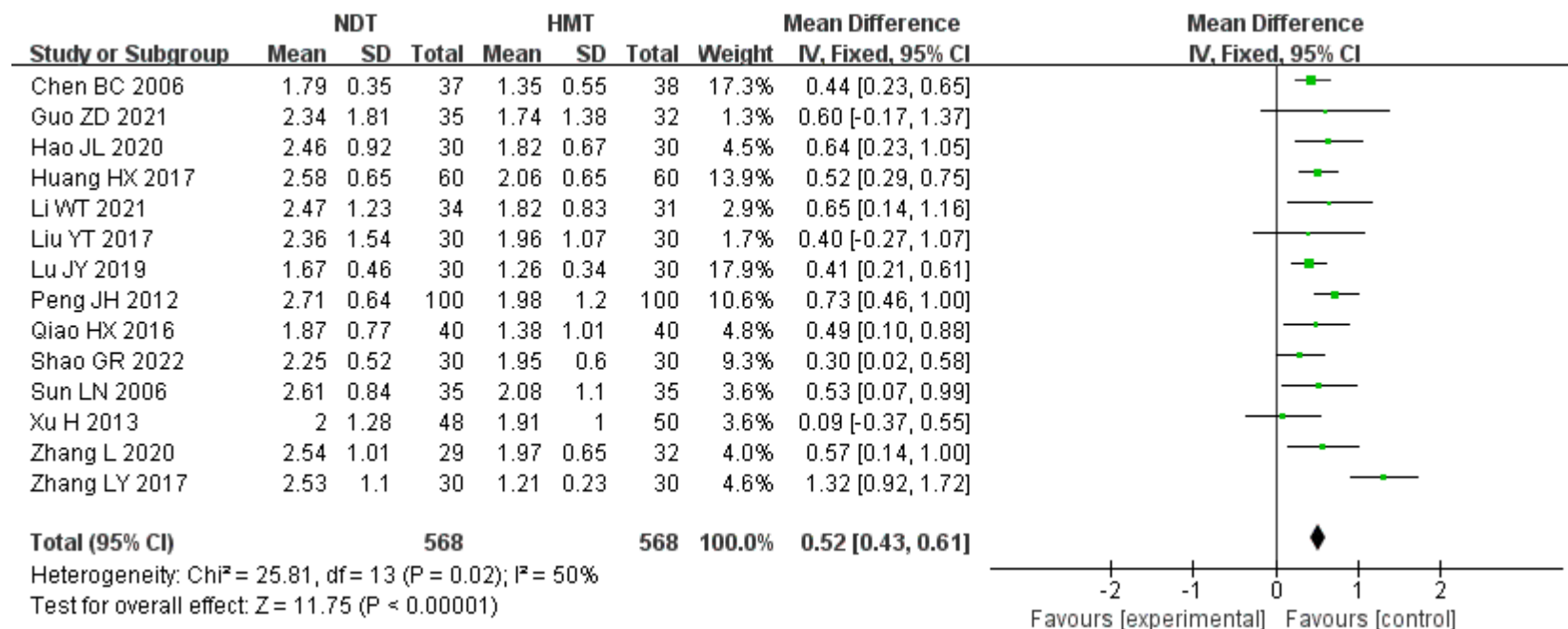

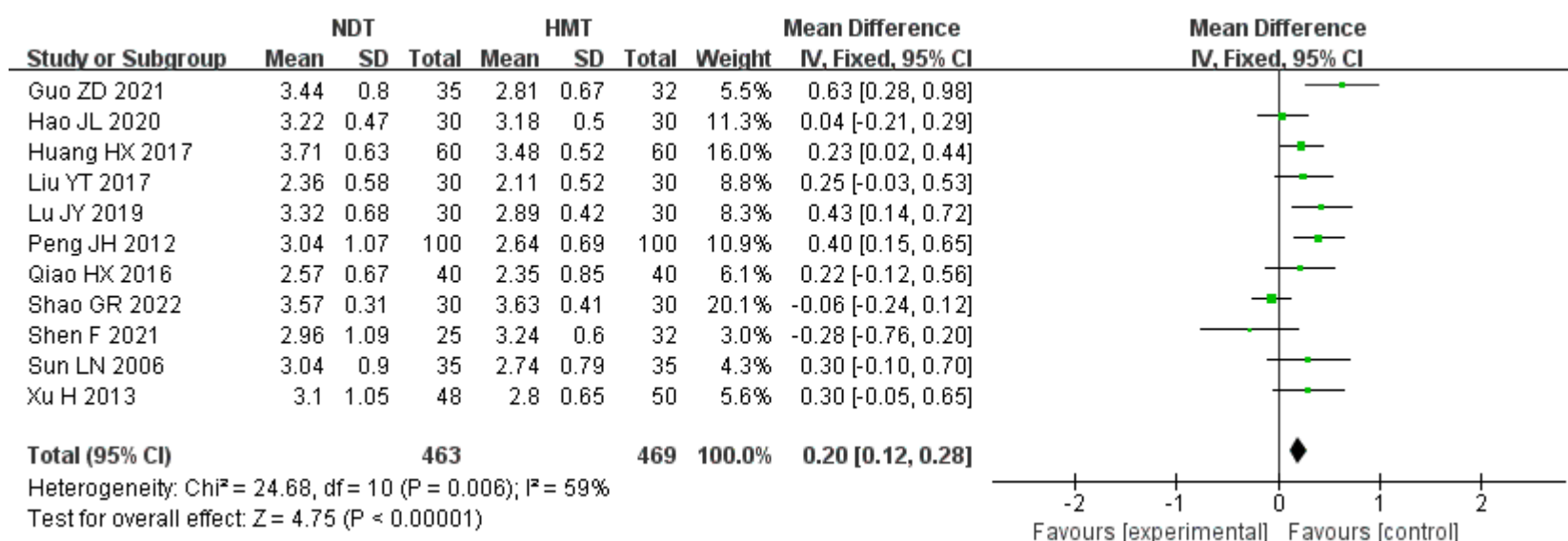

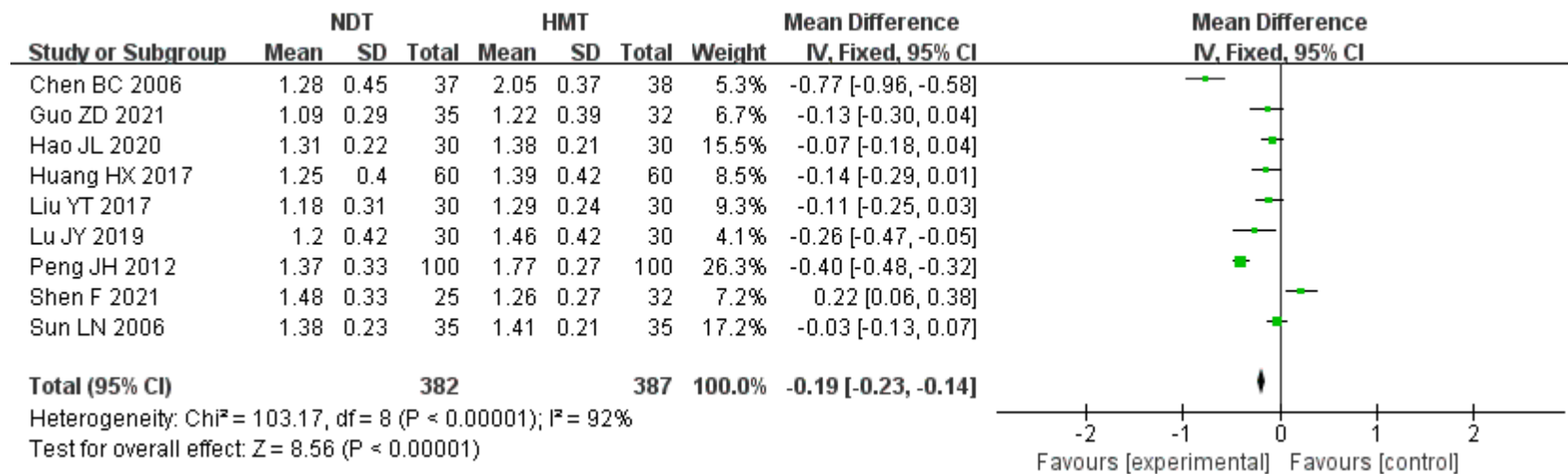

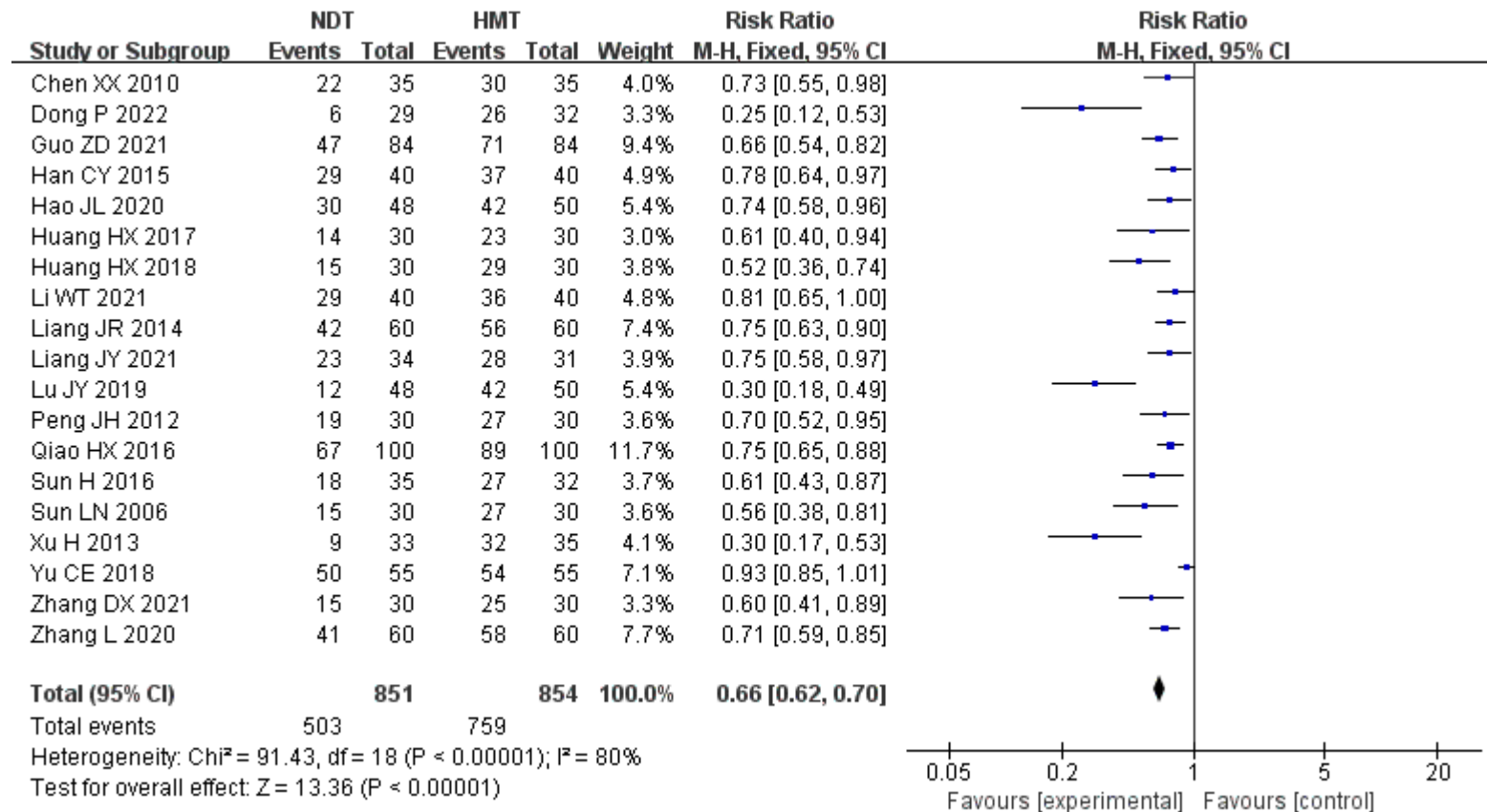

Supplement: Supplementary file 5 [file Supplementaryfile4.pdf]

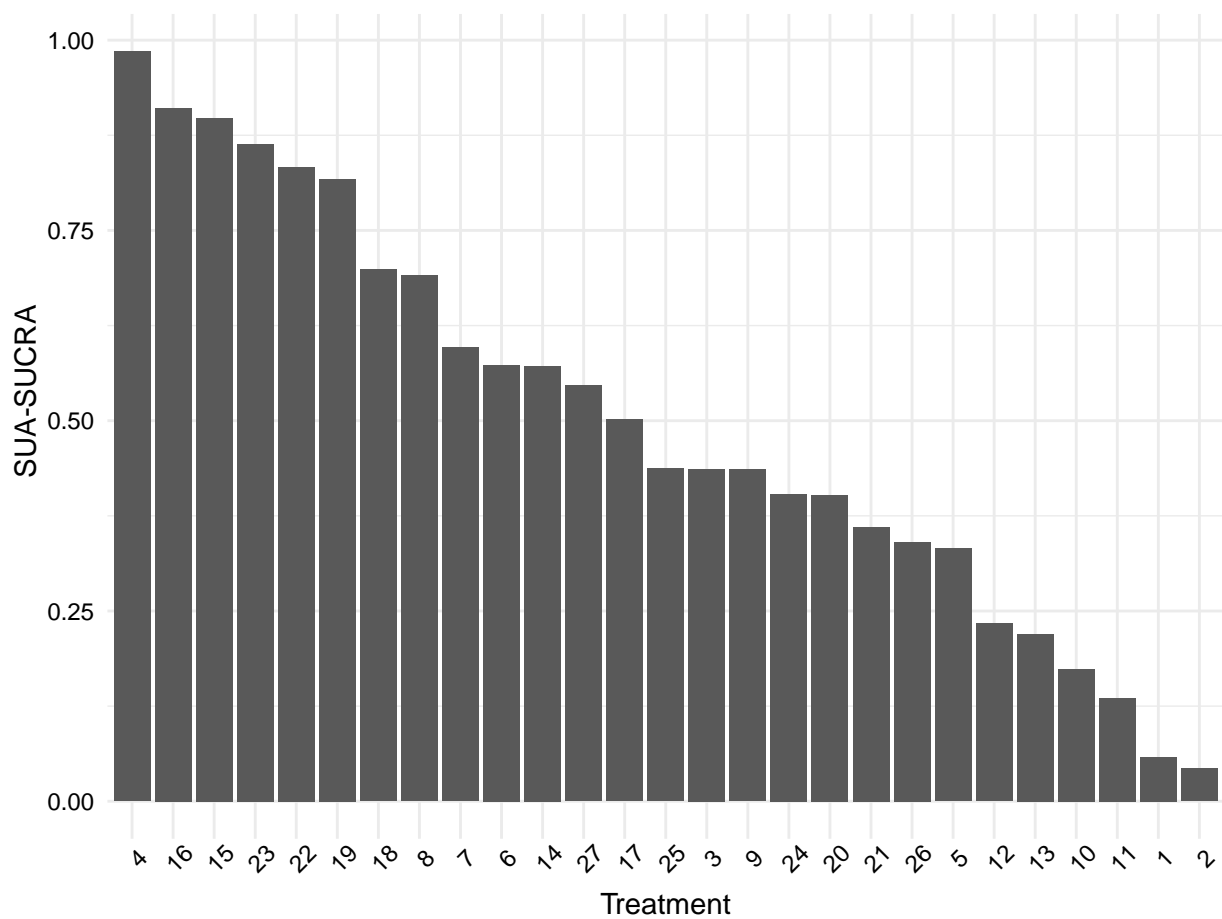

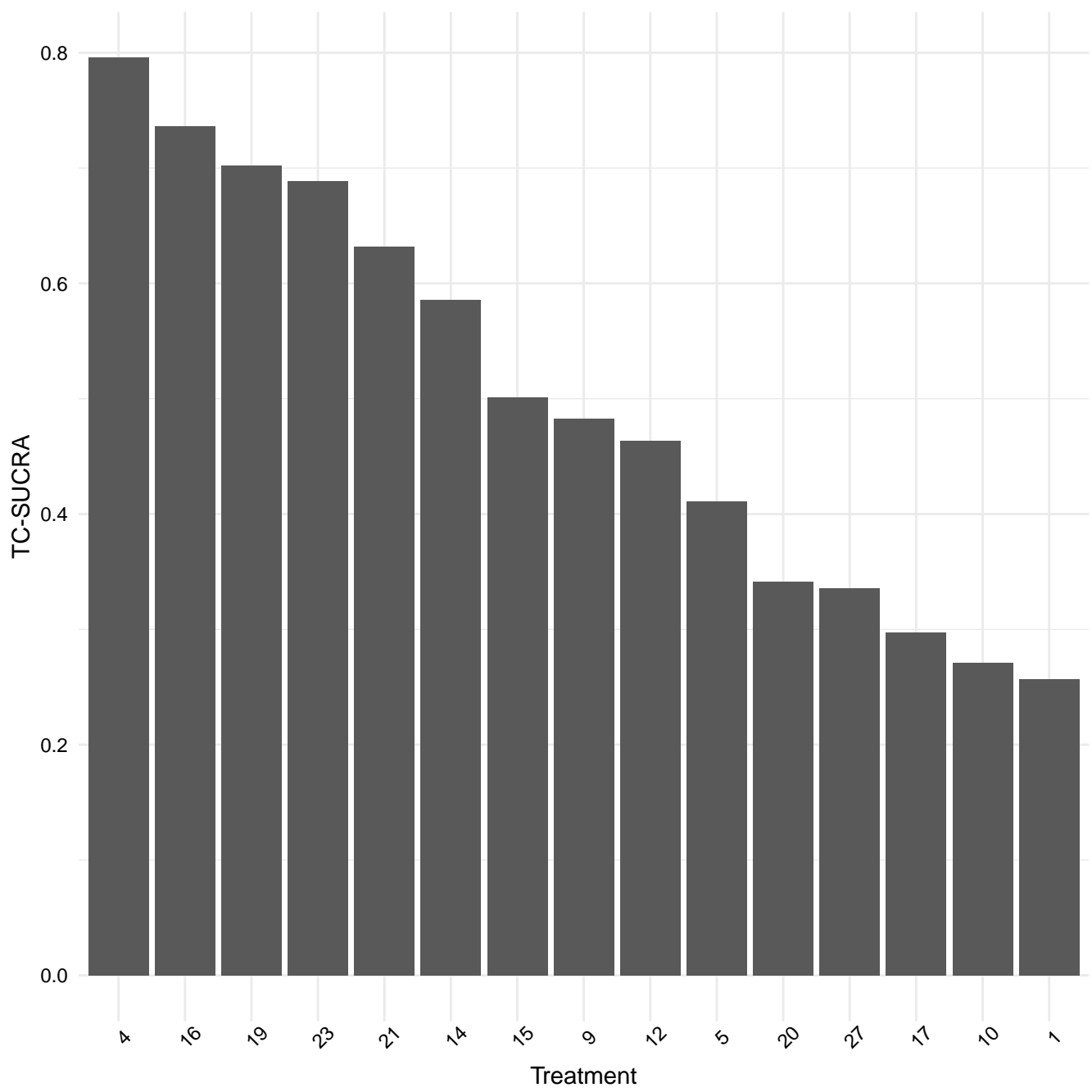

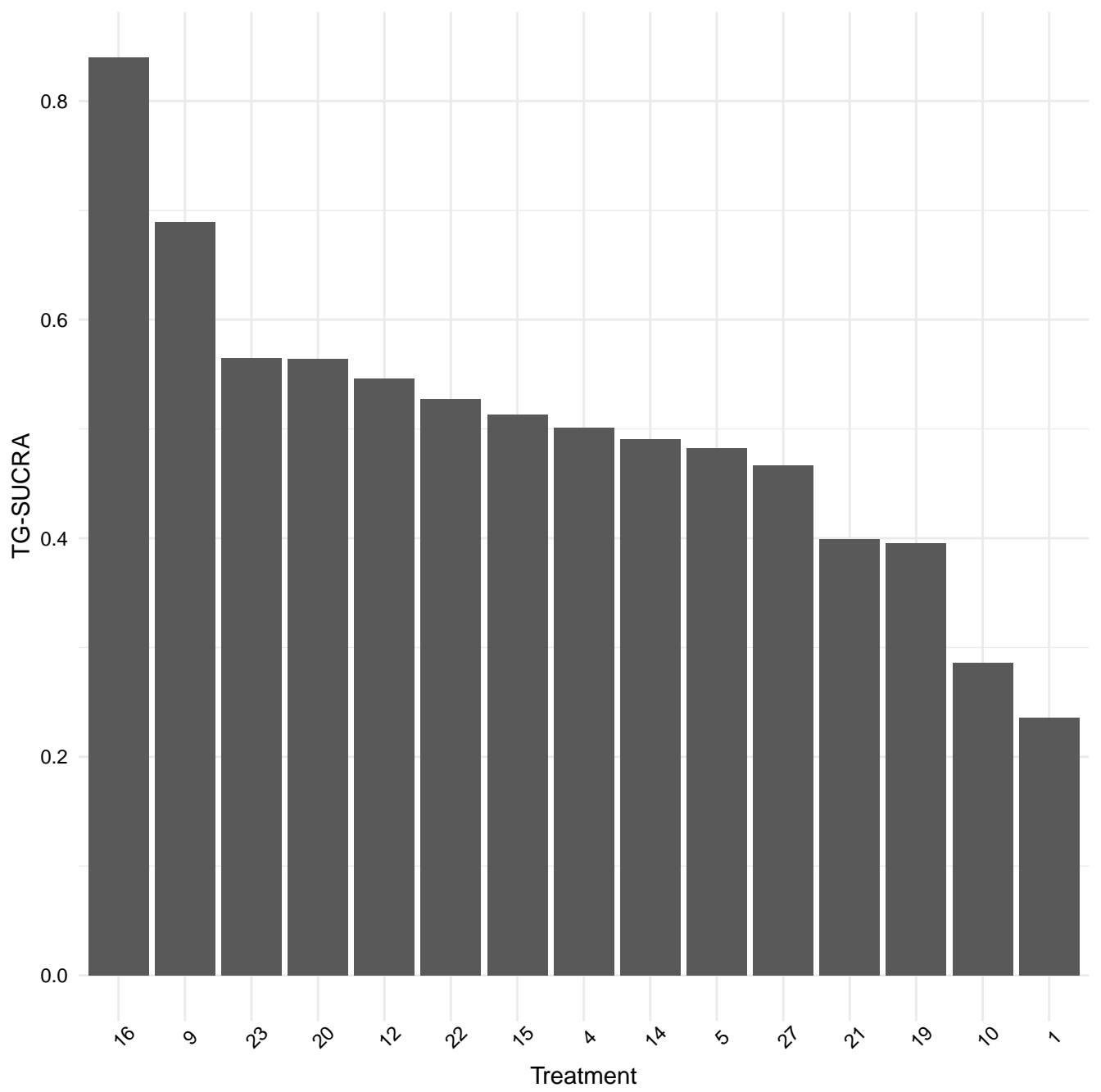

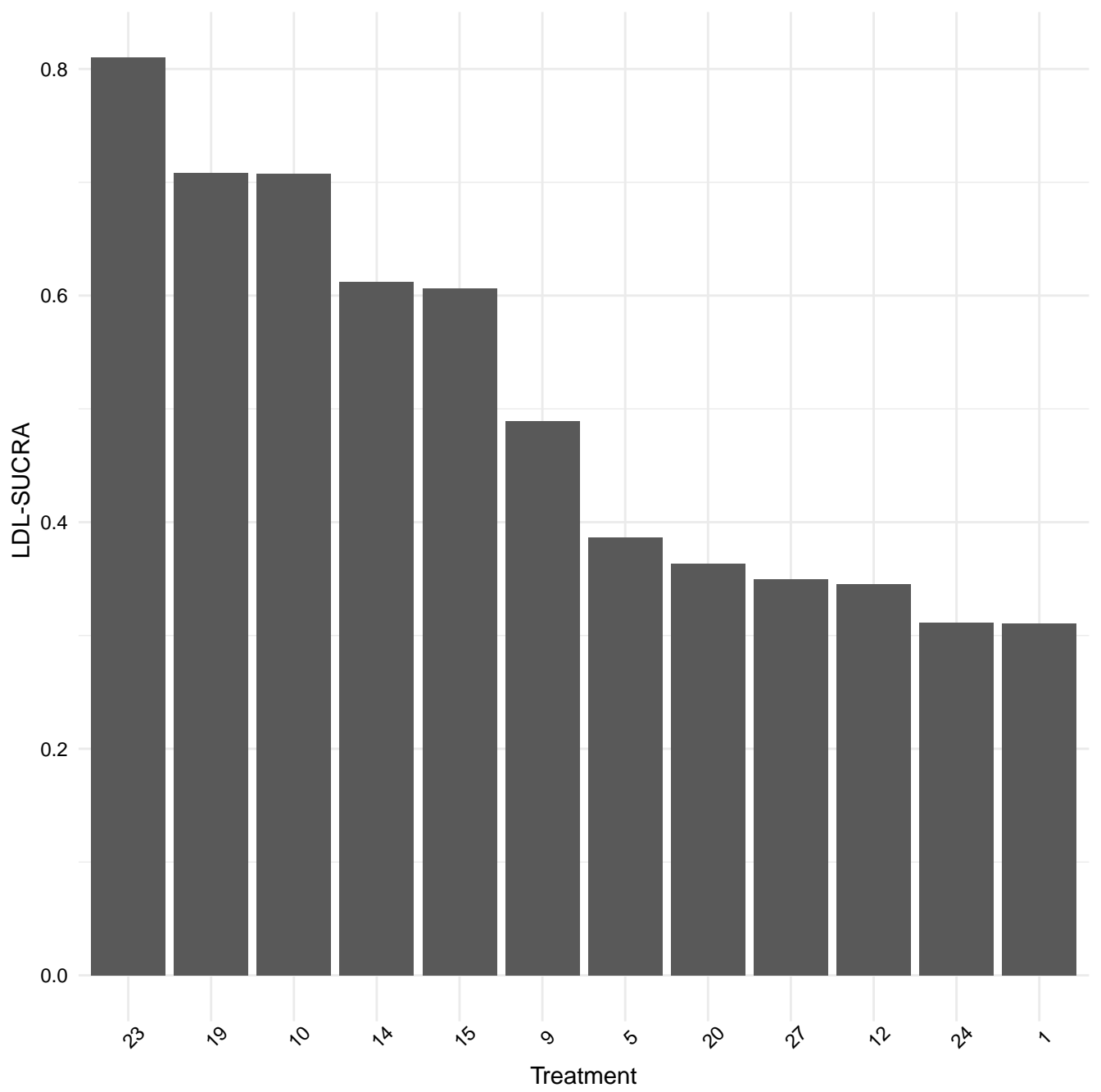

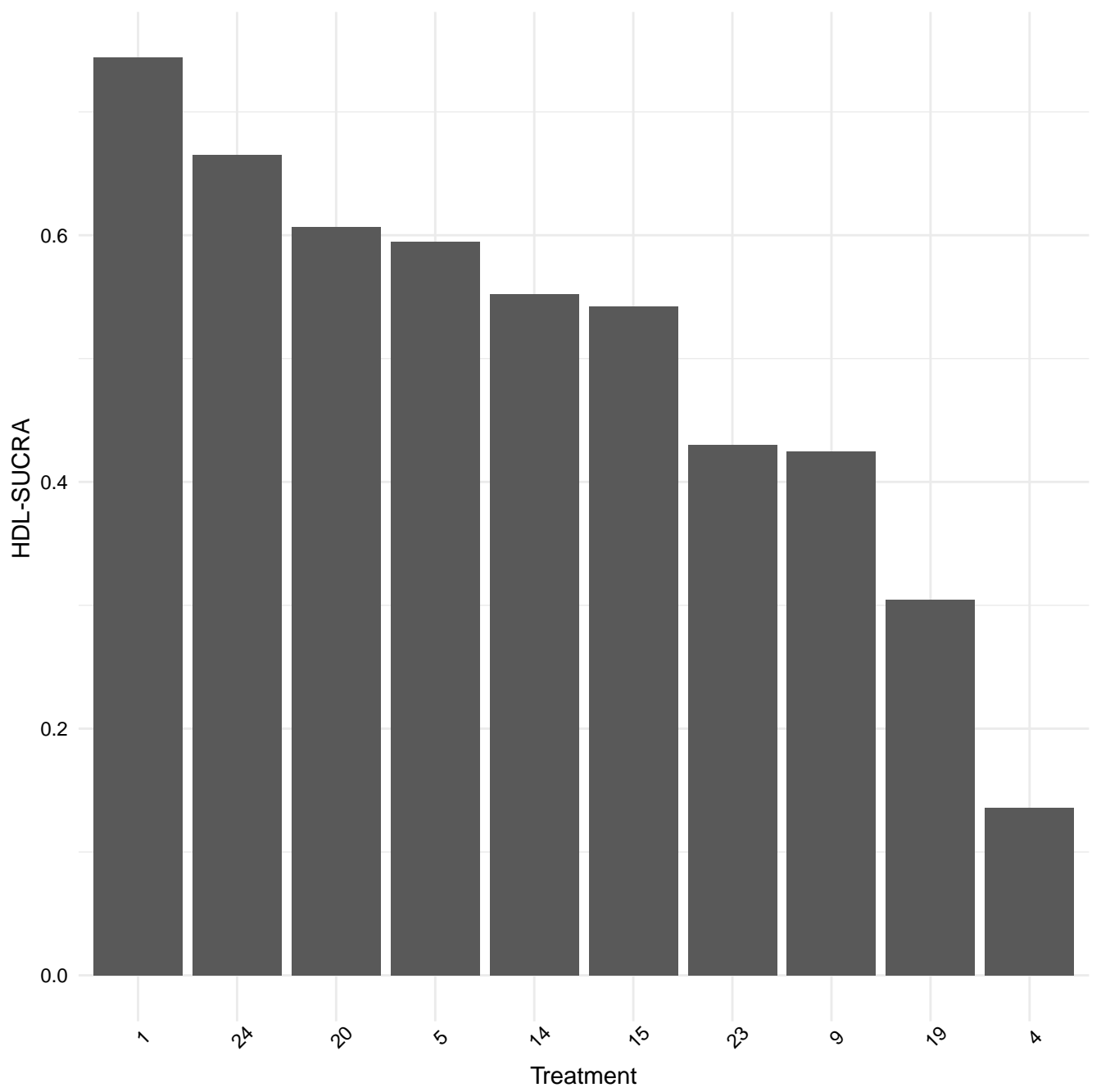

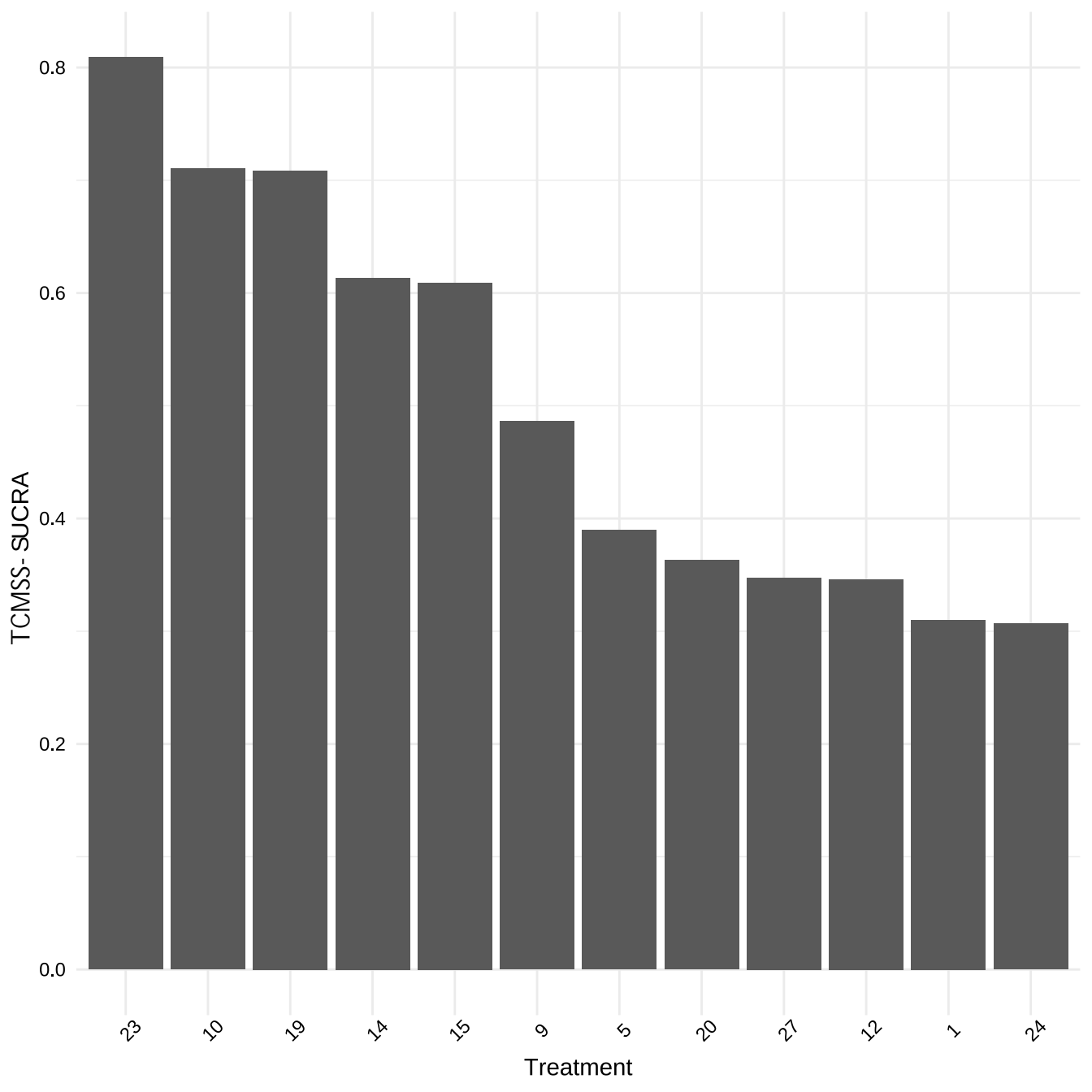

Supplement: Supplementary file 6 [file Supplementaryfile3.pdf]
