# Supplementary material for: Herbal medicine for asymptomatic hyperuricemia: a systematic review and network meta-analysis
Source: Front Pharmacol. 2025 Sep 29;16:1627714. doi: 10.3389/fphar.2025.1627714 (PMC12515837; doi:10.3389/fphar.2025.1627714)
Supplement: Supplementary file 7 [file Supplementaryfile6.pdf]

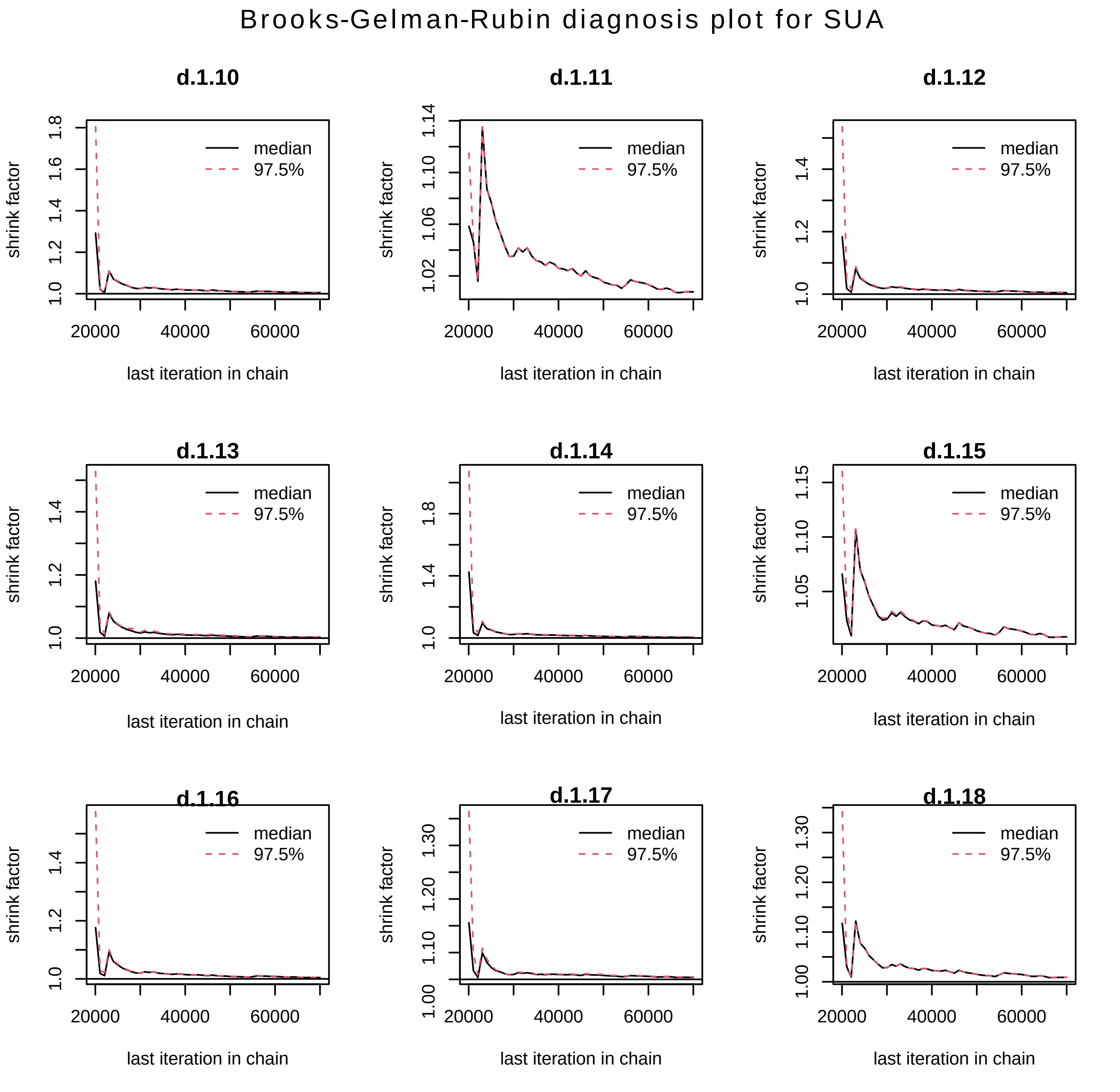
Brooks-Gelman-Rubin diagnosis plot for SUA
d.1.10
d.1.11
d.1.12
d.1.13
d.1.14
d.1.15
d.1.16
d.1.17
d.1.18
median
97.5%
shrink factor
last iteration in chain
20000
40000
60000
1.0
1.2
1.4
1.6
1.8
1.02
1.06
1.10
1.14
1.05
1.15
1.00
1.20
1.30

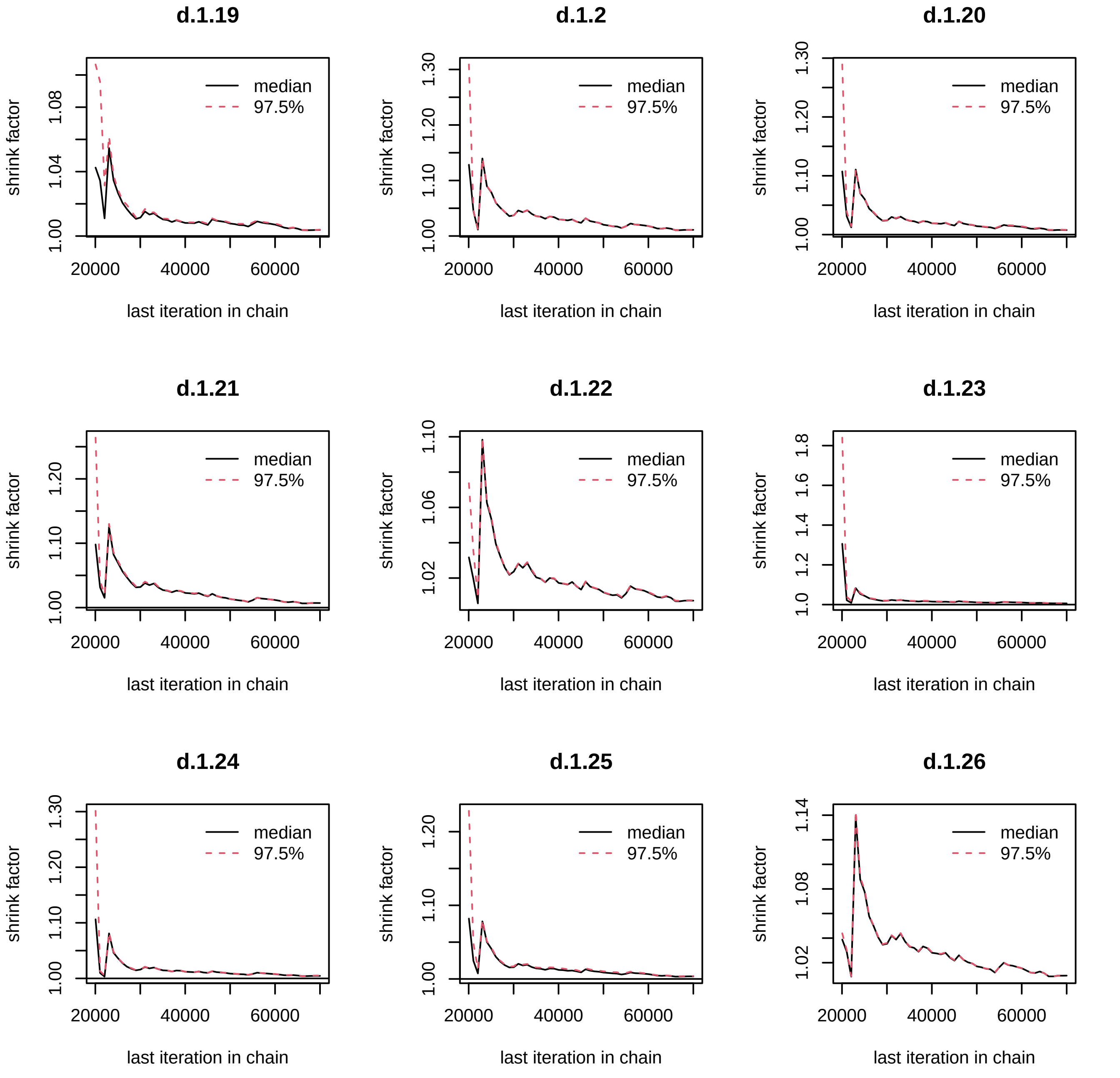
d.1.19
d.1.2
d.1.20
d.1.21
d.1.22
d.1.23
d.1.24
d.1.25
d.1.26
median
97.5%
shrink factor
last iteration in chain
20000
40000
60000
1.00
1.04
1.08
1.10
1.20
1.30
1.02
1.06
1.0
1.2
1.4
1.6
1.8
1.14

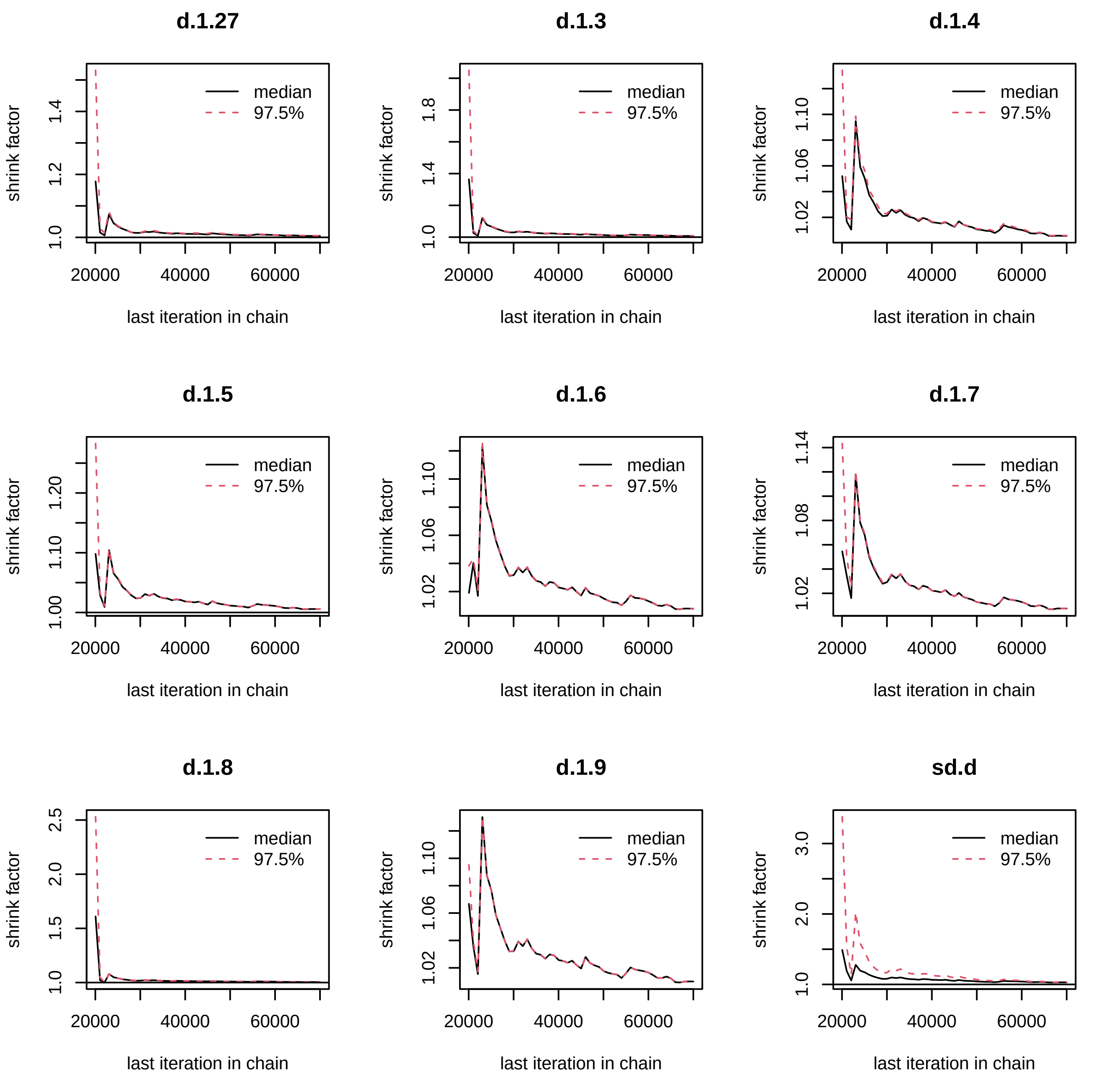
d.1.27
d.1.3
d.1.4
d.1.5
d.1.6
d.1.7
d.1.8
d.1.9
sd.d
median
97.5%
shrink factor
last iteration in chain
20000
40000
60000

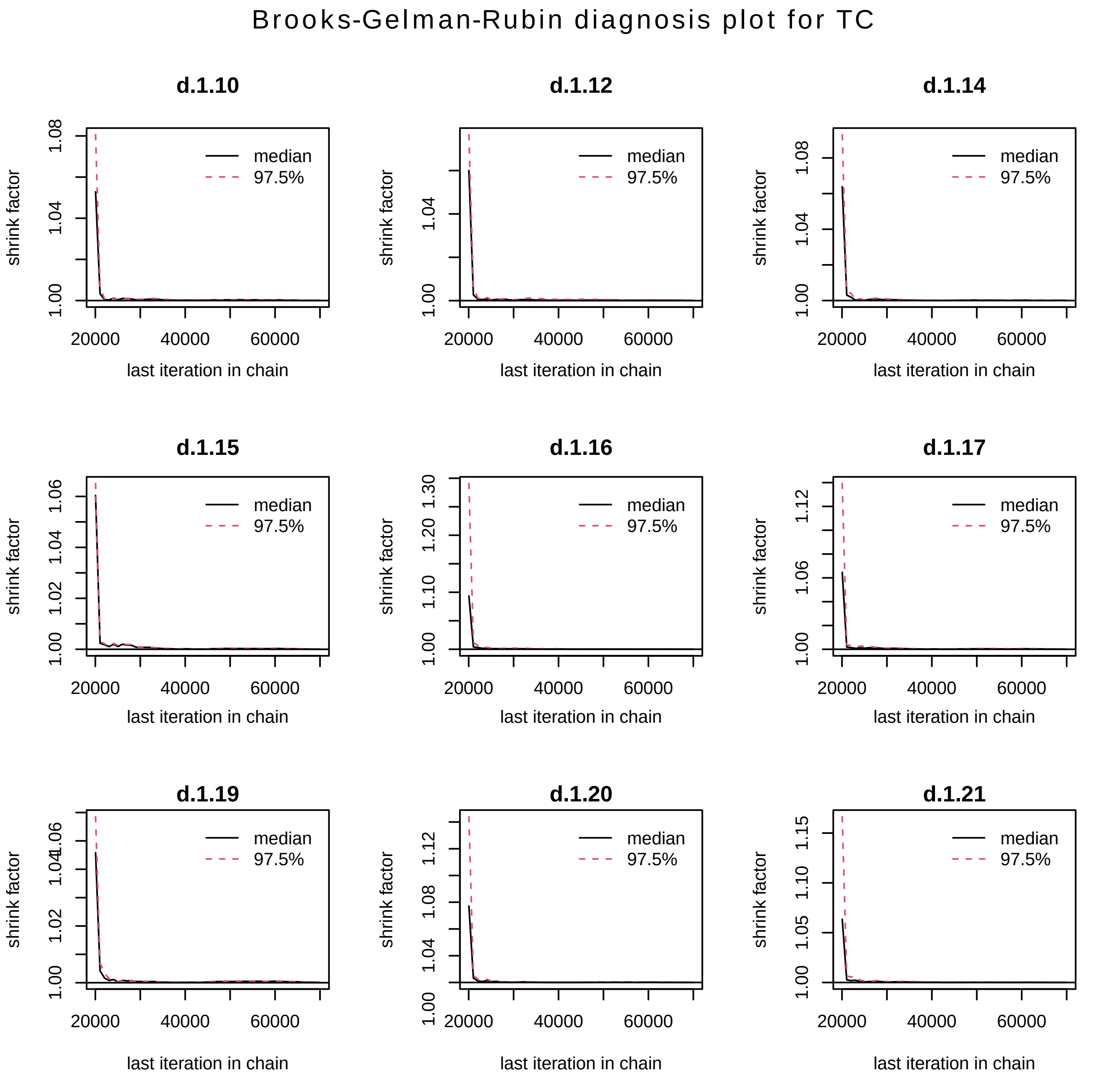
Brooks-Gelman-Rubin diagnosis plot for TC
d.1.10
d.1.12
d.1.14
d.1.15
d.1.16
d.1.17
d.1.19
d.1.20
d.1.21
median
97.5%
shrink factor
last iteration in chain
20000
40000
60000
1.00
1.02
1.04
1.05
1.06
1.08
1.10
1.12
1.15
1.20
1.30

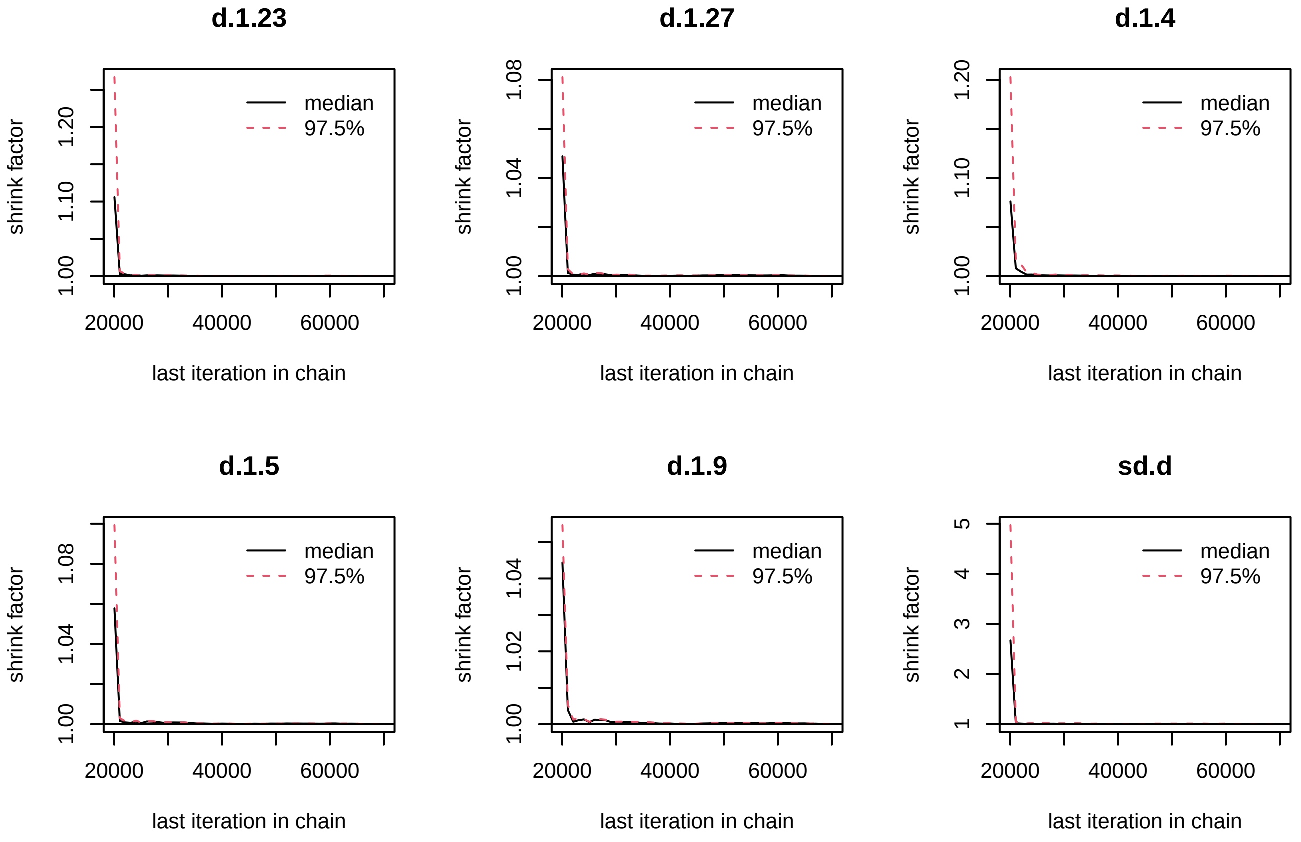
d.1.23
d.1.27
d.1.4
d.1.5
d.1.9
sd.d
median
97.5%
shrink factor
last iteration in chain
20000
40000
60000
1.00
1.10
1.20
1.04
1.08
1.02
1
2
3
4
5

Brooks-Gelman-Rubin diagnosis plot for TG

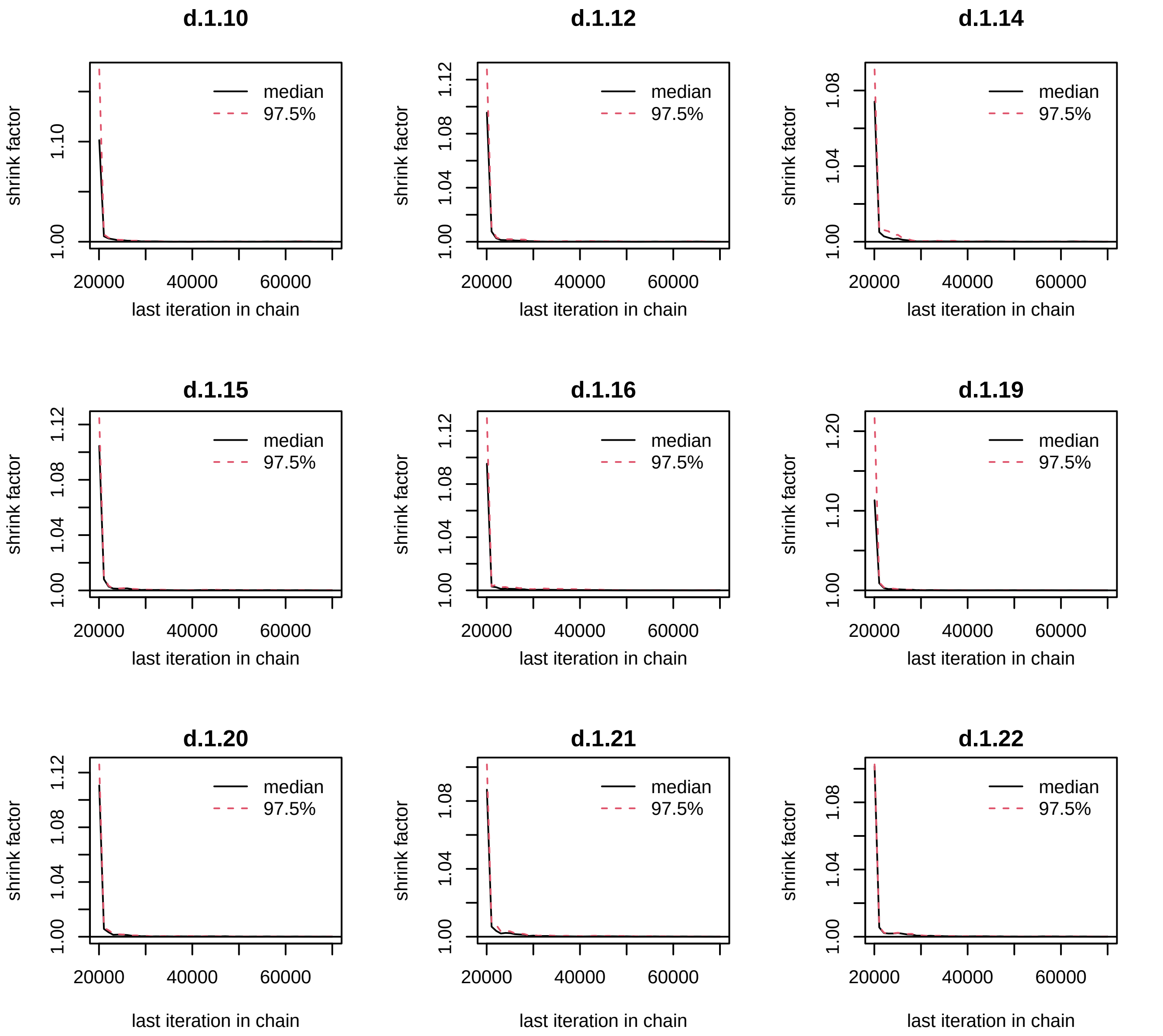

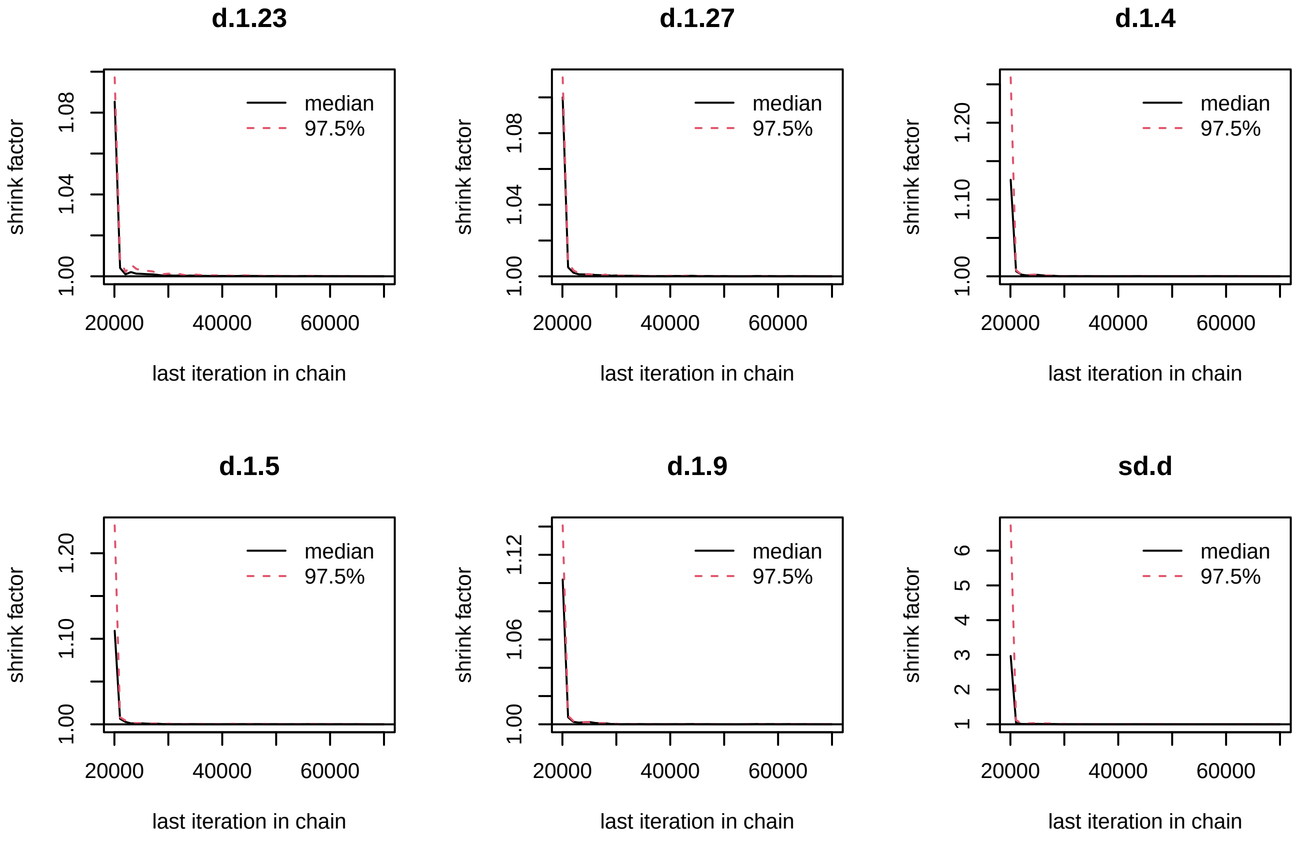
d.1.23
d.1.27
d.1.4
d.1.5
d.1.9
sd.d
shrink factor
last iteration in chain
median
97.5%
20000
40000
60000
1.00
1.04
1.08
1.10
1.20
1.06
1.12
1
2
3
4
5
6

Brooks-Gelman-Rubin diagnosis plot for LDL

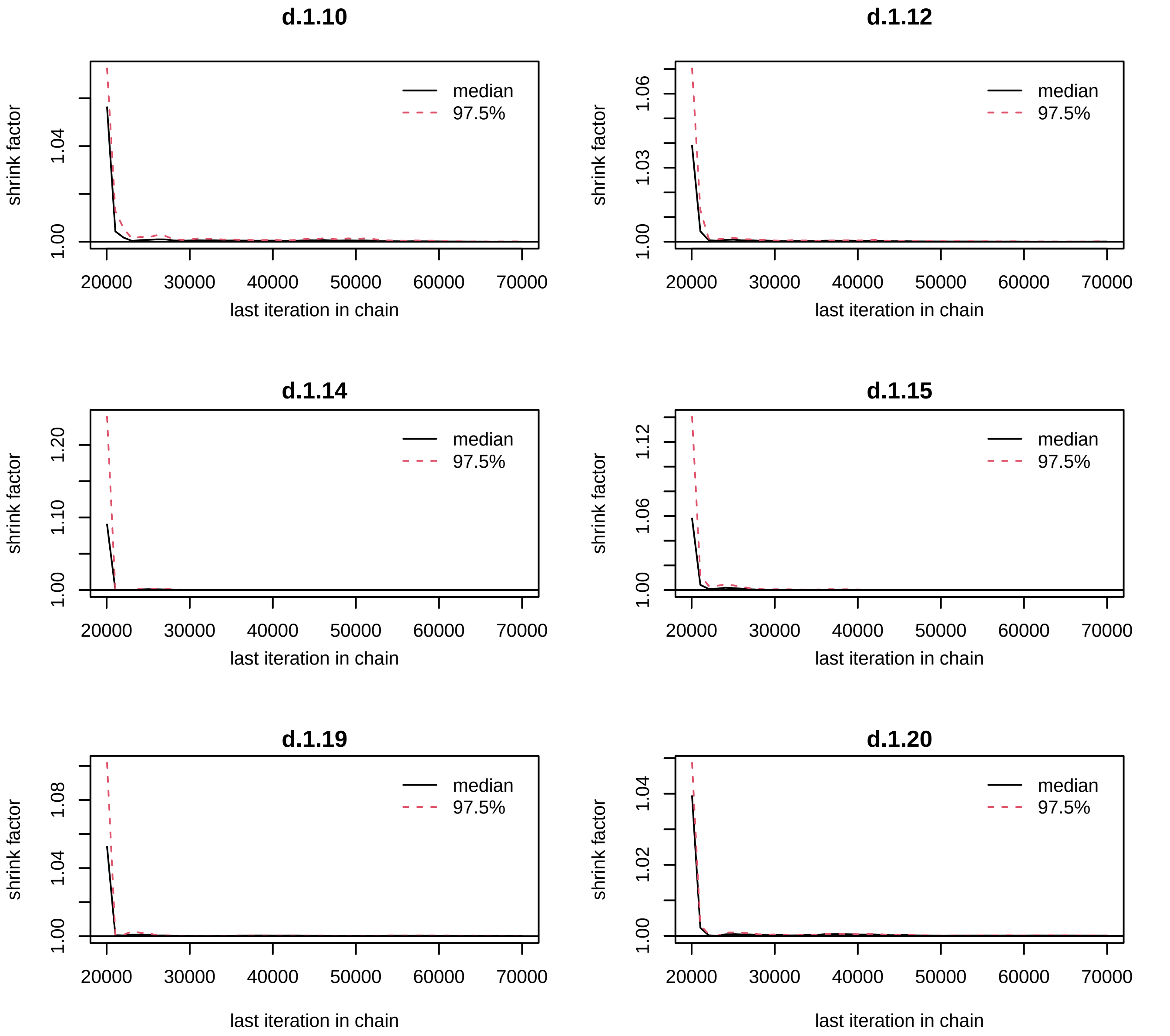

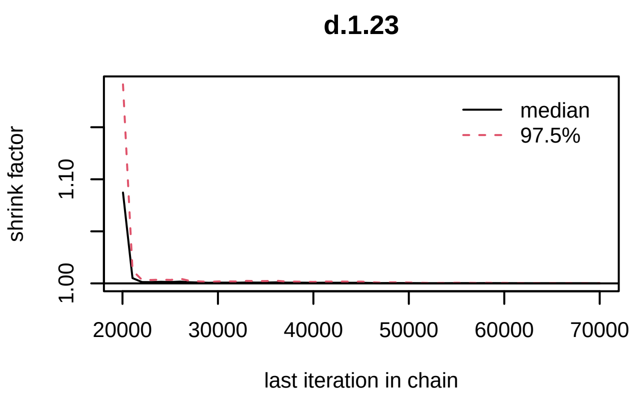
d.1.23
median
97.5%
shrink factor
1.00
1.10
20000
30000
40000
50000
60000
70000
last iteration in chain

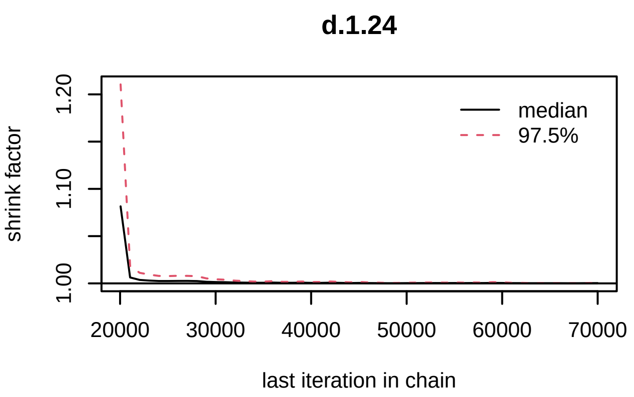
d.1.24
median
97.5%
shrink factor
1.00
1.10
1.20
20000
30000
40000
50000
60000
70000
last iteration in chain

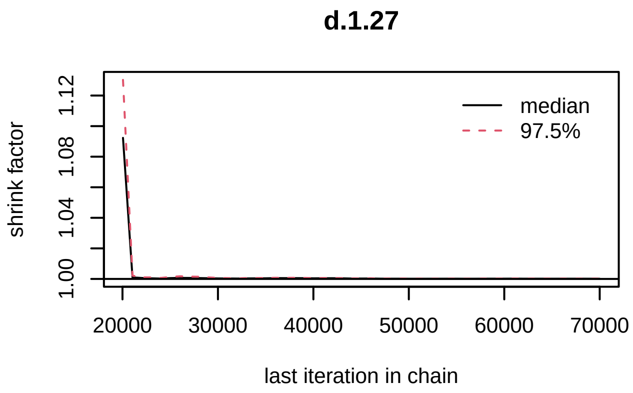
d.1.27
median
97.5%
shrink factor
1.00
1.04
1.08
1.12
20000
30000
40000
50000
60000
70000
last iteration in chain

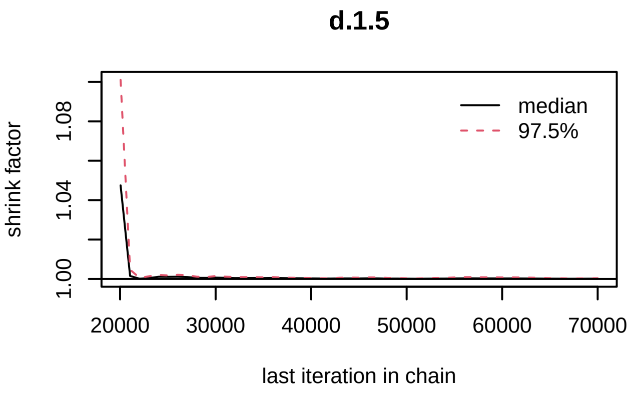
d.1.5
median
97.5%
shrink factor
1.00
1.04
1.08
20000
30000
40000
50000
60000
70000
last iteration in chain

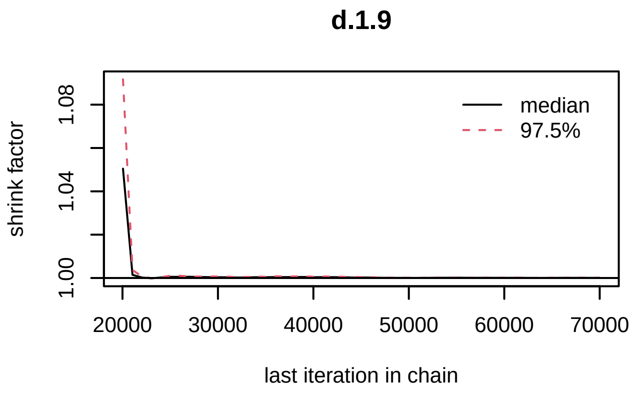
d.1.9
median
97.5%
shrink factor
1.00
1.04
1.08
20000
30000
40000
50000
60000
70000
last iteration in chain

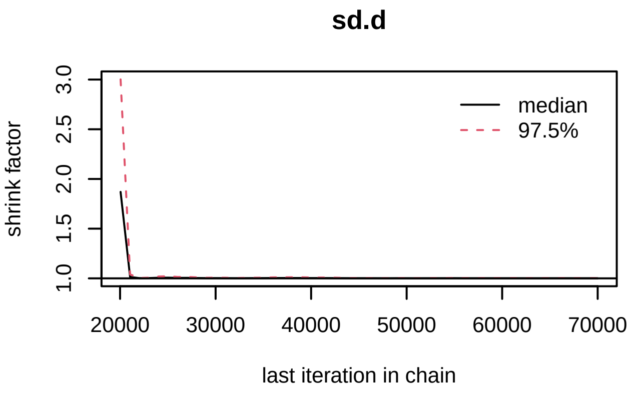
sd.d
median
97.5%
shrink factor
1.0
1.5
2.0
2.5
3.0
20000
30000
40000
50000
60000
70000
last iteration in chain

Brooks-Gelman-Rubin diagnosis plot for HDL

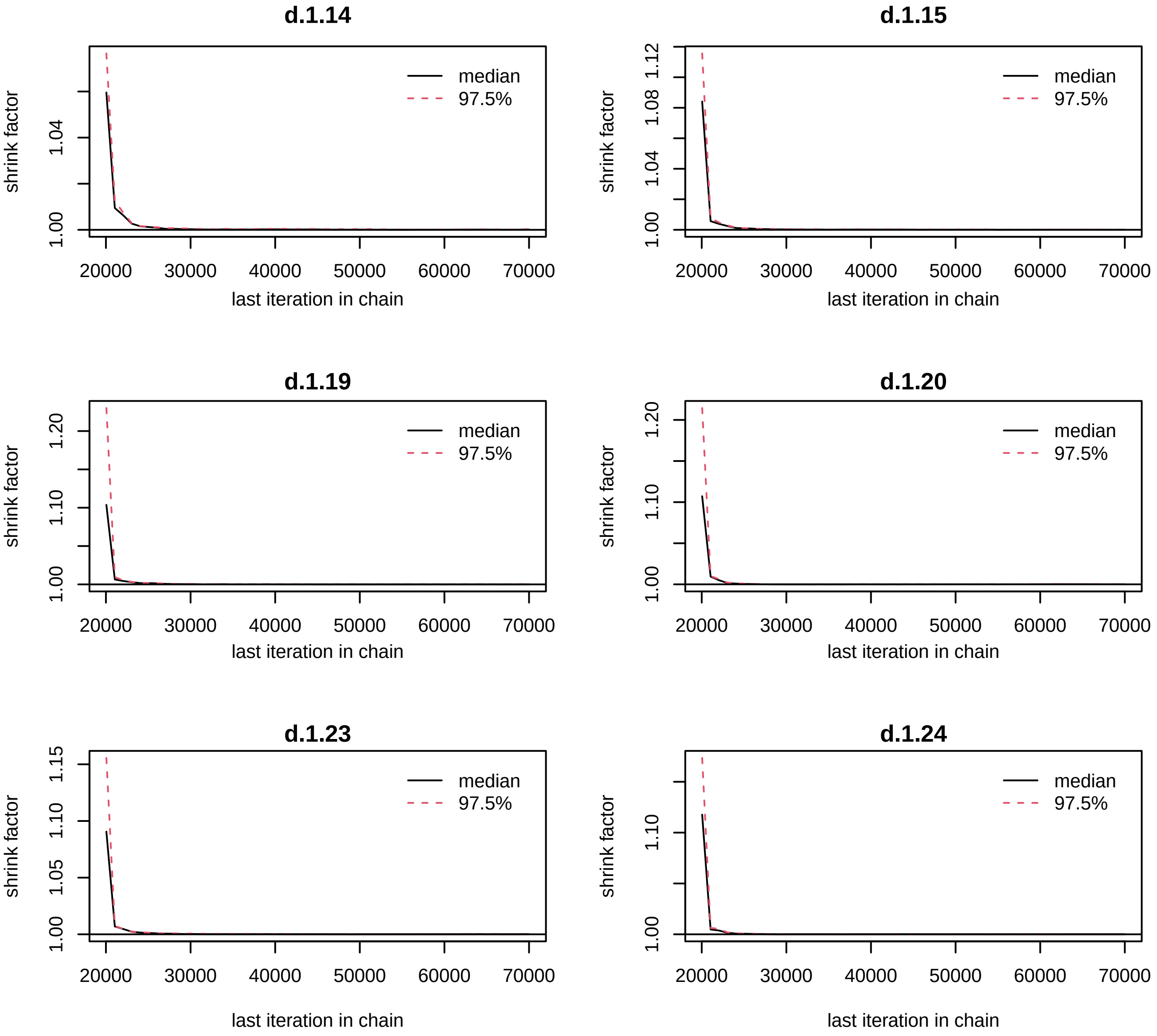

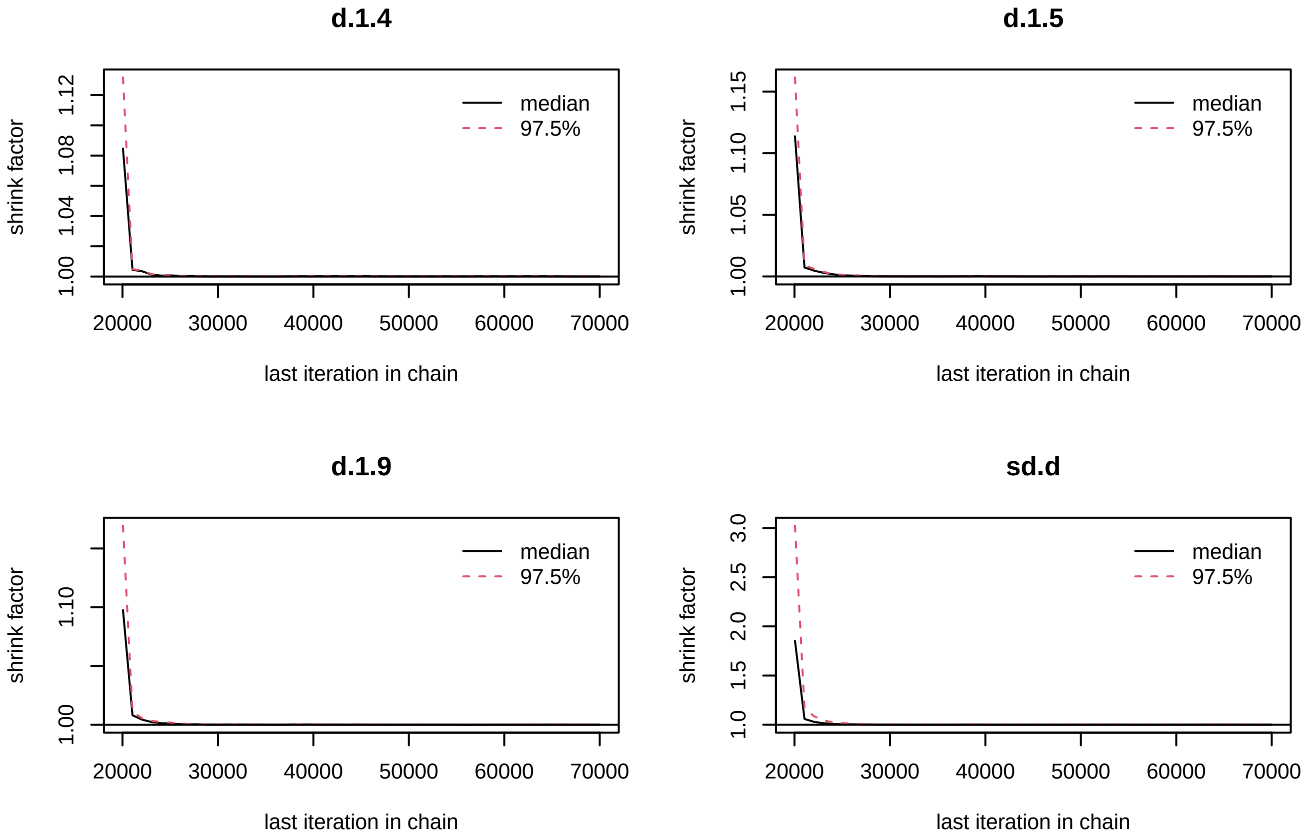

d.1.4
shrink factor
1.00
1.04
1.08
1.12
median
97.5%
20000
30000
40000
50000
60000
70000
last iteration in chain
d.1.5
shrink factor
1.00
1.05
1.10
1.15
median
97.5%
20000
30000
40000
50000
60000
70000
last iteration in chain
d.1.9
shrink factor
1.00
1.10
median
97.5%
20000
30000
40000
50000
60000
70000
last iteration in chain
sd.d
shrink factor
1.0
1.5
2.0
2.5
3.0
median
97.5%
20000
30000
40000
50000
60000
70000
last iteration in chain

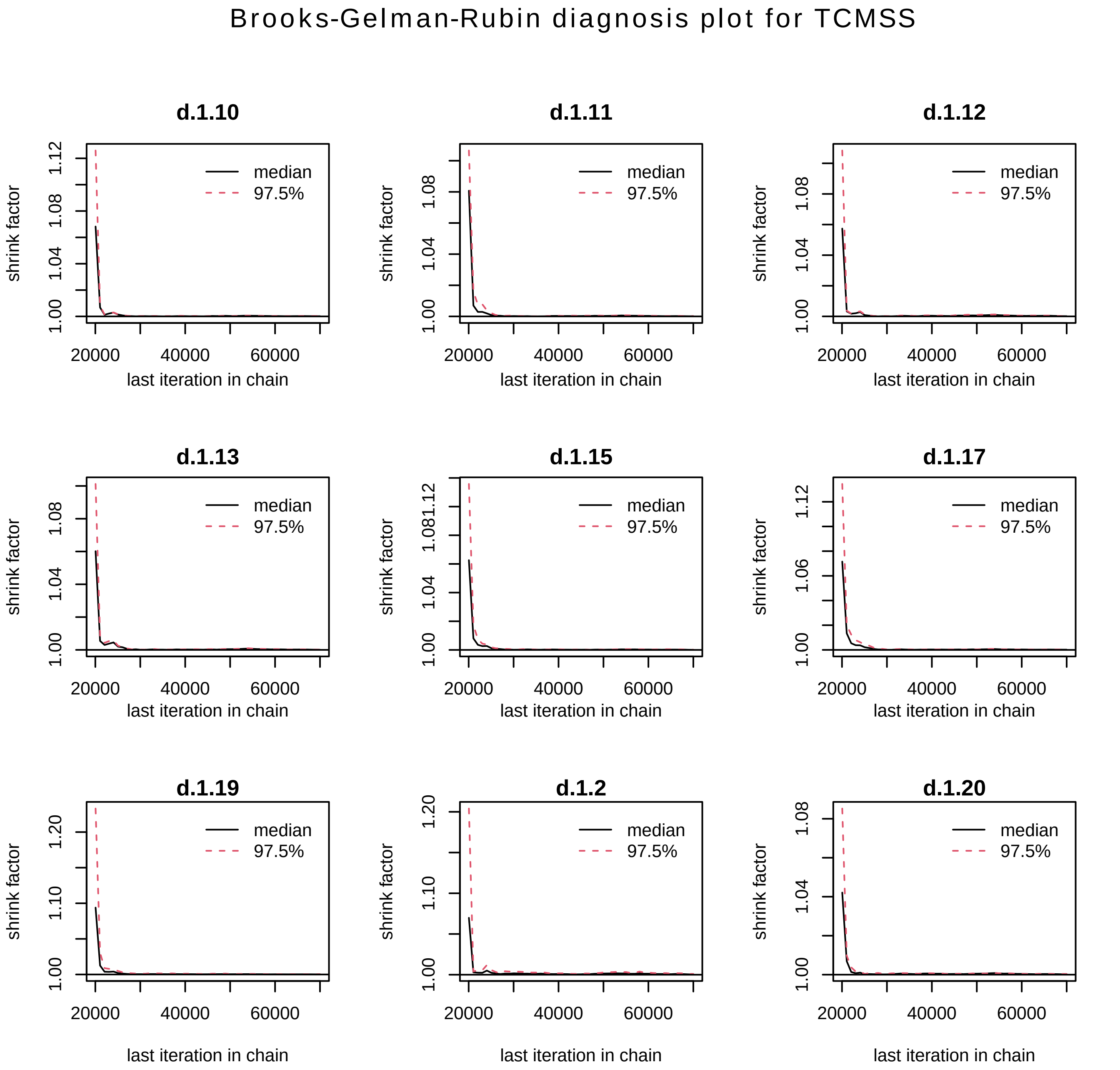
Brooks-Gelman-Rubin diagnosis plot for TCMSS
d.1.10
d.1.11
d.1.12
d.1.13
d.1.15
d.1.17
d.1.19
d.1.2
d.1.20
median
97.5%
shrink factor
last iteration in chain
20000
40000
60000
1.00
1.04
1.08
1.12
1.06
1.10
1.20

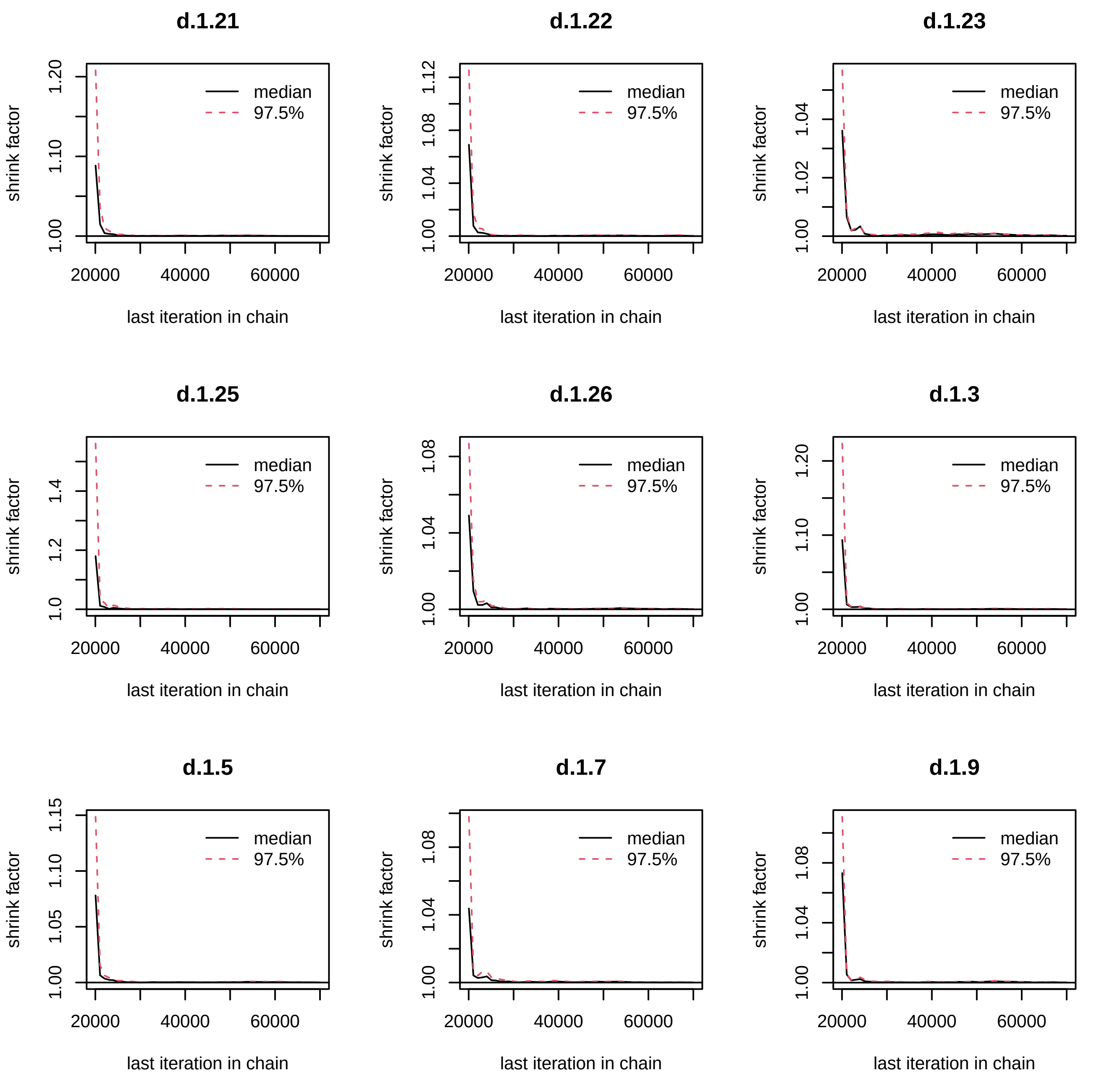
d.1.21
shrink factor
1.00
1.10
1.20
median
97.5%
20000
40000
60000
last iteration in chain
d.1.22
shrink factor
1.00
1.04
1.08
1.12
median
97.5%
20000
40000
60000
last iteration in chain
d.1.23
shrink factor
1.00
1.02
1.04
median
97.5%
20000
40000
60000
last iteration in chain
d.1.25
shrink factor
1.0
1.2
1.4
median
97.5%
20000
40000
60000
last iteration in chain
d.1.26
shrink factor
1.00
1.04
1.08
median
97.5%
20000
40000
60000
last iteration in chain
d.1.3
shrink factor
1.00
1.10
1.20
median
97.5%
20000
40000
60000
last iteration in chain
d.1.5
shrink factor
1.00
1.05
1.10
1.15
median
97.5%
20000
40000
60000
last iteration in chain
d.1.7
shrink factor
1.00
1.04
1.08
median
97.5%
20000
40000
60000
last iteration in chain
d.1.9
shrink factor
1.00
1.04
1.08
median
97.5%
20000
40000
60000
last iteration in chain

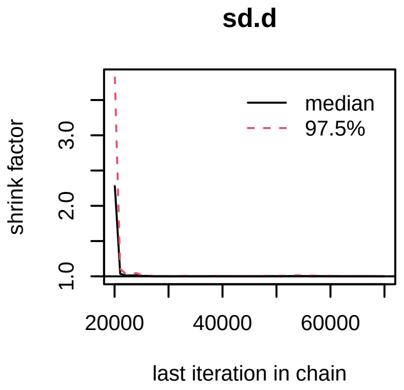
sd.d
median
97.5%
shrink factor
1.0
2.0
3.0
20000
40000
60000
last iteration in chain
